# Effectiveness of Six Improved Cookstoves in Reducing Household Air Pollution and Their Acceptability in Rural Western Kenya

**DOI:** 10.1371/journal.pone.0165529

**Published:** 2016-11-15

**Authors:** Tamara Pilishvili, Jennifer D. Loo, Stephanie Schrag, Debbi Stanistreet, Bryan Christensen, Fuyuen Yip, Ronald Nyagol, Robert Quick, Mike Sage, Nigel Bruce

**Affiliations:** 1 Centers for Disease Control and Prevention, Atlanta, Georgia, United States of America; 2 Department of Public Health and Policy, University of Liverpool, Liverpool, L69 3GB, United Kingdom; 3 Safe Water and AIDS Project, Kisumu, Kenya; Tsinghua University, CHINA

## Abstract

**Background:**

Household air pollution (HAP) from biomass fuel burning is linked to poor health outcomes. Improved biomass cookstoves (ICS) have the potential to improve HAP.

**Objectives:**

A pre-/post- intervention study assessed the impact of six ICS on indoor air quality and acceptability of ICS to local users in rural Western Kenya.

**Methods:**

We measured mean personal and kitchen level concentrations of particulate matter <2.5μm in diameter (PM_2.5,_ μg/m^3^) and carbon monoxide (CO, ppm) during the 48-hour period of each ICS use in 45 households. We compared these levels to those observed with traditional 3-stone fire (TSF) use. We assessed ICS acceptability through interviews and focus groups. We evaluated association of stove type, fuel use, and factors related to cooking practices with mean kitchen PM_2.5_ and CO using multivariable regression.

**Results:**

Stove type, exclusive ICS use (vs. concurrent TSF use), and the amount of fuel used were independently associated with kitchen PM_2.5_ and CO levels. Reductions (95%CI) in mean PM_2.5_ compared to TSF, ranged by ICS from 11.9% (-2.8–24.5) to 42.3% (32.3–50.8). Reductions in kitchen CO compared to TSF, ranged by ICS from -5.8% (-21.9–8.2) to 34.5% (23.2–44.1). Mean kitchen PM_2.5_ ranged from 319μg/m^3^ to 518μg/m^3^ by ICS. Women thought ICS were easy to use, more efficient, produced less smoke, and cooked faster, compared to TSF. Women also reported limitations for each ICS.

**Conclusions:**

We documented reductions in HAP from ICS compared to TSF. The PM_2.5_ levels with ICS use were still considerably higher than WHO indoor air quality guidelines. Achieving maximal potential of ICS requires adherence to more exclusive use and addressing user reported ICS limitations.

## Background

A growing body of evidence suggests a link between household air pollution and poor health outcomes in developing countries.[1–3] Nearly three billion people worldwide rely on solid fuels (wood, animal dung, crop wastes, and charcoal) as their main household fuel source, and in most cases this is burned on open fires or simple stoves with inadequate ventilation. The resulting household air pollution from biomass fuel burning is a potentially modifiable risk factor for childhood acute lower respiratory infections (ALRI). Observational studies and one clinical trial have demonstrated that improved-combustion stoves, improved ventilation, and reduced use of solid fuels would help reduce pneumonia morbidity and mortality in children.[1, 2, 4, 5] Recently published evidence on the relationships between particulate matter <2.5μm in diameter (PM_2.5_) exposure and risk of a range of diseases [6] suggest that reductions in exposure to ≤ 35μg/m^3^, intermediate target of annual average set by WHO, are needed to prevent the majority of attributable cases.[7]

A range of biomass stoves evaluated in a controlled laboratory setting by the US Environmental Protection Agency (EPA) have outperformed the open fire in terms of fuel efficiency, time required to boil water, and emissions of particulate matter and carbon monoxide (CO).[8] However, studies integrating evaluation of acceptability to local users with an assessment of impact of improved cookstoves (ICS) use on indoor air quality are lacking.[9] In addition, few studies measure directly household air pollution in the cooking area along with personal exposure monitoring, qualitative assessment of user perspectives, and can integrate these findings for a number of different makes of ICS. We conducted a study in a household setting in rural Western Kenya to determine whether everyday use of the six ICS, which in a laboratory setting reduce emissions by at least 50% compared to the open fire, would deliver levels of PM_2.5_ and CO associated with substantially reduced health risks. We sought to determine both the acceptability of these stoves to local users as well as their effectiveness in reducing indoor concentrations and personal exposure.

## Methods

### Study design

We conducted a single-arm pre-/post- intervention study to assess acceptability and performance of six ICS in a setting of daily stove use. In order to limit the inter-household variability in household air pollution levels related to individual household practices, size of the household, and ventilation due to house structure, we employed a cross-over design which allowed for the evaluation of up to six ICS within one household.

### Study population

This study was implemented in two rural villages in Nyando Division, Nyanza Province, Western Kenya. Nyando Division has a population of approximately 80,000 people and 15,000 households.

Households with women of childbearing age (15–49 years old) and one or more children aged <5 years were identified in the two participating villages. To detect a significant (**α =** 0.05) 20% reduction in mean PM_2.5_ with the use of ICS in the household compared to TSF (paired sample), assuming 80% power and 40% coefficient of variation, 30 households needed to be enrolled for each ICS. Assuming each household tested 5–6 ICS and 10% attrition, 43 households were randomly selected from a list of eligible households in the two villages. Women who provided written consent to participate in the study were enrolled. Women 15–18 years old who were pregnant, married, or a parent were considered “mature minors” and were able to consent for their own participation in the study. Home visits were made to the enrolled households to conduct interviews to assess acceptability of ICS and to measure personal and kitchen level exposures to indoor air pollutants during the 48-hour baseline and follow-up monitoring periods.

### Data collection

#### Household visits

Each household tested up to six ICS for two weeks per stove with a one-week break in between. We varied the order in which the stoves were tested in each household. Prior to installation of the ICS, the primary cooks in the household completed a brief questionnaire on current stove use, cooking practices, fuel collection and consumption, and socio-demographic information. Women were trained to use each ICS with a standardized training guide. Their traditional stove (TSF) remained in the home but the women were encouraged to use only the ICS for daily cooking. At the end of each 2-week period, we conducted individual interviews to gather information on stove use patterns, ease of use with local cooking pots, perceptions of smoke levels, cleanliness, safety, and taste, acceptability of cooking methods, comfort and ergonomics when preparing local dishes, and general perception of fuel consumption.

#### Study stoves

The ICS included in this study were EcoZoom, Prakti (chimney stove), Envirofit, Philips and Ecochula (both forced draft with rechargeable battery and solar-PV panel), and a locally-made ceramic stove, colloquially known as the rocket stove, with a thermoelectric insert enhancement (Rocket with TECA) (Appendix 1 in S1 File).[10] All ICS selected for the study performed well at the EPA laboratory (#x2265;50% reduction in PM_2.5_ emissions compared to TSF)[8], were centrally manufactured, required no assembly, could be easily transported, were designed to burn wood, and were considered acceptable by local women during pilot cooking tests conducted prior to study initiation. The traditional TSF was employed as the baseline comparison cooking method.

#### In-depth interviews and focus groups

Views on stove characteristics, including efficiency, fuel consumption, health effects, cooking behaviors, and user acceptability were assessed through 262 structured interviews and 11 focus groups. Structured interviews conducted after each two-week period of ICS use assessed acceptability. Focus groups carried out after households had tested four stoves (round 4) and again at the end of the study explored participants’ views on stove functionality, design and acceptability, as well as reasons for multiple stove use (i.e., concurrent use of TSF along with ICS).[11]

#### Kitchen area and personal air sampling

Air pollution monitoring was conducted at baseline (TSF) and for each ICS during the final 48 hours of the two-week intervention period. The air monitoring consisted of kitchen area air sampling and personal area air sampling. Concurrent 48-hour measurements of gravimetric PM_2.5_, and real-time CO were conducted in the kitchen. The instrumentation was placed on the wall at 1.5 meters from the ground (i.e., approximately the height of the breathing zone). A time-integrated gravimetric PM_2.5_ sample was collected using an active pump (Casella, Buffalo, NY, USA) with a BGI Triplex Cyclone (BGI Incorporated, Waltham, MA, USA), and 37 mm Teflon membranes (Pall, Port Washington, NY, USA) (Appendix 2 in S1 File). Gravimetric analysis of the filters was conducted after conditioning in temperature- and humidity-controlled environments for 24 hours. Concomitant real-time CO measurements over a 48-hour period were conducted using a GasBadge Pro (Industrial Scientific, Oakdale, PA, USA), with detection limits between 0–1,500 ppm, set at one-minute intervals; the mean of the measurements taken over the 48-hour sampling period was calculated.

Concomitantly with the indoor measurements, personal CO exposure was monitored in real-time. The GasBadge was worn by the woman and positioned near her breathing zone on her upper chest. The participants were instructed to wear the monitors at all times except when sleeping or bathing, when they placed the monitors next to their bed or bathing area. We assessed compliance to personal monitor use through unscheduled daily visits to participating households during the 48-hour monitoring period. We excluded personal samples collected when GasBadge was reported not to be worn. These exclusions represented <5% of all samples.

#### Improved stove use

We asked women to complete time activity diaries during each 48-hour monitoring period recording the duration of stove use (minutes) for each cooking episode, type of stove used, type of meal prepared, number of people cooked for, and duration of kerosene lamp use (hours). We also employed temperature data loggers to gather objective data on stove use for both the improved stoves and the traditional stove and to complement findings from time activity diaries. The results of stove use monitoring were reported separately.[12] We measured the amount of fuel used during each 48-hour monitoring period. Women participants were asked to collect sufficient fuel to last for 3 days; the collected fuel was weighed at the beginning and at the end of the 48-hour period.

### Data analysis

The cross-over design allows for within household comparisons of the effects of ICS on indoor air quality, and therefore, adjusts for time-independent factors, such as socioeconomic and demographic factors, house structure and ventilation. In addition, we adjusted for the effect of time (rounds of follow up approximately three weeks apart) on CO and PM_2.5_ concentrations.

We estimated geometric mean concentrations for 48-hour gravimetric time-weighted PM_2.5_ concentrations (μg/m3) and kitchen and personal (woman) CO real-time (ppm) obtained within households using each ICS type and for TSF within the corresponding households. We estimated changes in 48-hour mean (and median) kitchen PM_2.5_ and in kitchen and personal (woman) CO for each ICS compared to TSF within each household. The same analysis was repeated, stratifying by multiple stove use (i.e., “stove stacking”) as reported by women using time-activity diaries. The data for PM_2.5_ and CO were not normally distributed; therefore, comparisons employed the paired t-test with log-normal distribution. We also conducted sensitivity analysis using the sign test.

We used linear mixed effect models with log PM_2.5_ or log CO as the dependent variable to evaluate the association of ICS type with kitchen concentrations of PM_2.5_ and CO. The variability due to unexplained “between-household” differences was modeled as a random effect, allowing for “within household” comparisons between follow-up periods with improved stoves and TSF. The analysis was adjusted for time-dependent variables, such as multiple stove use (i.e., “stove stacking”), average duration of cooking events, number of meals prepared, number of people cooked for, and amount of fuel used. The variables significant at α = 0.05 were retained in the final model. We evaluated potential confounding by time-dependent and time-independent variables in the final model. Estimated regression coefficients (and 95% confidence intervals) were exponentiated and subtracted from one to calculate adjusted percent reduction in 48-hour PM_2.5_ and CO concentration for each improved stove type compared to TSF, use of improved stove only compared to multiple stove use, and per unit change for continuous variables.

We used SAS 9.3 software for quantitative data analysis and Dedoose software (SocioCultural Research Consultants 2014) for qualitative data analysis.

Recordings of structured interviews and focus groups were translated by field workers from Luo to English, and subsequently transcribed. A thematic approach to data analysis was taken, drawing on published methods.[13] All interviews were coded and analyzed initially by round to identify any changes to findings over time. Data from each round were coded to the point of saturation for each stove and each theme.[11]

#### Funding source and ethical considerations

Funding for the study was provided by the Centers for Disease Control and Prevention and The Morgan Stanley Foundation. The study was approved by the Institutional Review Boards of the Kenya Medical Research Institute (protocol number 2075) and the Centers for Disease Control and Prevention (protocol number 6155). The Institutional Review Board of the Centers for Disease Control and Prevention provided overall ethical oversight and approved the entire study.

## Results

We identified 58 households meeting the eligibility criteria out of 181 households (total population 840) in the two participating villages. Forty-three households were randomly selected through initial draw, four were deemed ineligible following the initial visit (two did not have age-eligible children, one was planning to relocate during the study period, and one did not have a designated area for cooking) and were not enrolled. The replacement households were selected by randomly drawing from the remaining pool of eligible households. Three households dropped from the study (one each following the second, third, and fourth rounds). Two additional households were subsequently selected from the remaining pool and baseline assessment was repeated for the newly enrolled households. In total, 45 households participated in the study: 7 households received all 6 of the study stoves, 30 received 5 stoves, and the remaining 8 households received 2–4 stoves. Participating women were 17 to 45 years of age (mean (SD) age 28 years (7)); 38 (88%) were married and the remaining 5 (12%) were single mothers or widowed. The majority of women (93%) were comfortable with reading or writing, and 58% had completed at least primary education. Fifteen women (35%) farmed their own land, 9 (21%) owned their own business, 6 (13%) worked as day laborers, and the remaining 13 (30%) ran the household (Table 1).

**Table 1:** Characteristics of participating households.

**Household characteristics (N = 45)**	
**Average number of members (range)**	6.0 (3–10)
**Average number of children <5 (range)**	1.9 (1–3)
**Water source**	
Pump	16 (37%)
Well	10 (23%)
Communal standpipe	7 (16%)
**Collect from river**	10 (23%)
**Access to drinking water**	43 (100%)
**Sanitation**	
Latrine in the yard	31 (72%)
Shower/bath in house	11 (26%)
**Avg. weekly expenditures per household (KSH)**	1381.08 (150–5,000)
**Possessions**	
Radio	26 (60%)
TV	8 (19%)
CD player	3 (7%)
Bicycle	26 (60%)
Motorbike	4 (9%)
Car or truck	1 (2%)
Cell phone	33 (77%)
Access to electric generator	2 (5%)
Cow (one or more)	23 (53%)
Land purchased	13 (30%)

No changes were noted in the average number of daily meals prepared, number of people cooked for, and duration of kerosene lamp use by follow up period and by stove type. Reductions in the average time spent cooking a meal were observed with all ICS compared to TSF, except for the Prakti (Table 2). Significant reductions (p<0.01) in average fuel consumed were found for all ICS.

**Table 2:** Stove use, cooking practices, and fuel consumption during the 48-hour monitoring period by stove type.

	Baseline (TSF), N = 45	Ecochula, N = 36	Envirofit, N = 35	EcoZoom, N = 37	Philips, N = 35	Prakti, N = 39	Rocket with TECA, N = 35
Fuel consumed, average (range, kg)	12.0 (3.1–28.8)	7.5 (2.4–20.5)	9.3 (1.9–17.9)	8.5 (2.0–28.9)	5.3 (0.6–11.9)	9.5 (2.1–41.1)	8.2 (3.4–19.9)
Kerosene lamp use, average (range, hours)	6.1 (2–11)	6.2 (2–12)	6.5 (3–16)	6.1 (3–12)	5.9 (2–10)	6.2 (3–15)	5.8 (2–12)
Time spent cooking a meal, average (range, min)	82 (28–180)	66 (35–128)	68 (28–125)	70 (28–125)	61 (21–127)	84 (37–181)	80 (42–187)
Number of cooking events, average (range)	7 (2–14)	6.1 (1–12)	7.5 (4–14)	7.5 (4–13)	6.6 (4–13)	6.6 (4–15)	6.3 (4–11)
Number of people cooked for, average (range)	5.5 (3–10)	5.4 (2–9)	5.4 (3–10)	5.5 (3–10)	5.5 (2–10)	5.4 (2–9)	5.5 (3–9)
Reported using TSF along with the improved cook stove, N (%)	N/A	13 (36)	10 (29)	10 (27)	12 (34)	18 (46)	13 (37)

### Stove type, multiple stove use, and other factors associated with air quality

Reductions in mean PM_2.5_ ranged from 109μg/m^3^ observed with EcoZoom to 357μg/m^3^ with Philips, with statistically significant reductions observed for four out of six ICS compared to TSF (Table 3). The largest mean reductions in kitchen CO of 3.4 ppm and personal CO (woman) of 1.7 ppm were observed with use of Envirofit and Ecochula, respectively. The largest median reduction in kitchen CO (2.7 ppm) was observed using Philips, with statistically significant reductions in kitchen CO observed only in households using Envirofit, Philips, and Prakti. Using univariate regression analysis, and accounting for correlated response within household by follow up period, stove type and amount of fuel used were significantly associated with mean kitchen PM_2.5_ (Table 4). Each additional hour of kerosene lamp use was associated with 5% increase in mean kitchen PM_2.5_. Univariable analysis of stove type, amount of fuel used, kerosene lamp use, average number of people cooked for, and average duration of cooking episode demonstrated significant associations with mean kitchen CO levels (Table 5).

**Table 3:** Gravimetric PM_2.5_ (μg/m^3^) and kitchen and personal (woman) CO real time (ppm) 48-hour concentration by stove type.

	Gravimetric kitchen PM_2.5_ (μg/m^3^)					CO real-time kitchen (ppm)					CO real-time personal (ppm)				
Stove type	N	Baseline^a^	Follow up^b^	Difference, mean (median)	p-value^c^	N	Baseline^a^	Follow up^b^	Difference, mean (median)	p-value^c^	N	Baseline^a^	Follow up^b^	Difference, mean (median)	p-value^c^
Ecochula	36	621	518	116 (205)	0.2403	34	6.8	5.4	1.7 (1.1)	0.1379	31	2.5	1.0	1.7 (1.2)	**<0.0001**
Envirofit	35	618	398	277 (186)	**0.0044**	34	6.7	4.9	3.4 (2.1)	**0.0041**	30	2.4	1.1	1.3 (1.2)	**0.0001**
EcoZoom	37	609	503	109 (143)	0.1663	37	6.6	6.7	-0.2 (1.0)	0.9136	31	2.2	1.3	0.7 (0.7)	**0.0003**
Philips	35	604	319	357 (294)	**0.0002**	35	6.5	3.8	2.7 (2.7)	**0.0069**	29	2.1	1.1	0.6 (1.0)	**0.0014**
Prakti	39	588	374	118 (280)	**0.0036**	37	6.6	4.5	0.7 (2.3)	**0.0190**	32	2.0	0.9	0.9 (0.8)	**0.0008**
Rocket with TECA	35	571	368	215 (213)	**0.0121**	34	6.0	4.4	2.5 (1.4)	0.0602	31	2.3	1.4	0.8 (0.8)	**0.0289**

^a^ Baseline measurements in a setting of 3-stone fire use (geometric mean)

^b^ Measurements in a setting of improved stove use (geometric mean)

^c^ Paired t-test, assuming lognormal distribution

**Table 4:** Factors associated with 48-hour mean gravimetric PM_2.5_ (μg/m^3^) concentration.

	Univariable analysis		Multivariable model^2^	
Variable	Percent reduction (95%CI) in mean PM_2.5_	p-value	Percent reduction (95%CI) in mean PM_2.5_	p-value
Stove type^1^				
Ecochula	18.0 (5.1, 29.2)	0.1768	11.9 (-2.8, 24.5)	0.4122
Envirofit	35.6 (25.7, 44.2)	0.0024	36.1 (26.1, 44.8)	0.0023
EcoZoom	19.7 (7.6, 30.2)	0.1199	14.9 (1.7, 26.4)	0.2640
Philips	45.2 (36.6, 52.6)	<0.0001	42.3 (32.3, 50.8)	0.0007
Prakti	38.6 (29.5, 46.5)	0.0005	41.1 (31.9, 48.9)	0.0003
Rocket with TECA	31.9 (21.1, 41.3)	0.0099	32.7 (21.5, 42.2)	0.0107
Use of improved stove only (vs. multiple stove use) during the follow up period	12.8 (3.1, 21.6)	0.1976	29.0 (21.2, 31.1)	0.0013
Fuel consumed during the 48-hour monitoring period (kg)	-3.4 (-4.4, -2.5)	0.0003	-3.0 (-4.0, -2.0)	0.0023
Kerosene lamp use, average (hours)	-4.9 (-2.3, -7.6)	0.0583	-	-
Average number of people cooked for	-7.1 (-11.7, -2.7)	0.1063	-	-
Average time spent cooking a meal (min)	-0.3 (-0.5, -0.1)	0.0944	-	-
Number of cooking events	-0.5 (-2.9, 1.8)	0.8182	-	-

^1^Reference category: 3-stone fire; overall p-value for stove type p = 0.0005

^2^Mixed effects model, accounting for correlated response within household by follow up period

**Table 5:** Factors associated with 48-hour mean real-time kitchen CO (ppm) concentration.

	Univariable analysis		Multivariable model^2^	
Variable	Percent reduction (95%CI) in mean CO	p-value	Percent reduction (95%CI) in mean CO	p-value
Stove type^1^				
Ecochula	21.5 (9.1, 32.2)	0.0995	14.1 (-0.2, 26.3)	0.324
Envirofit	27.6 (16.6, 37.2)	0.0237	27.9 (16.7, 37.5)	0.0241
EcoZoom	1.9 (-12.6, 14.6)	0.8875	-5.8 (-21.9, 8.2)	0.6935
Philips	38.5 (28.9, 46.7)	0.0009	34.5 (23.2, 44.1)	0.0082
Prakti	32.3 (22.3, 41.0)	0.0051	33.5 (23.3, 42.4)	0.0047
Rocket with TECA	25.1 (13.2, 35.3)	0.0508	24.6 (12.3, 35.2)	0.0637
Use of improved stove only (vs. multiple stove use) during the follow up period	11.1 (1.3, 19.9)	0.2621	27.5 (19.4, 34.7)	0.0027
Fuel consumed during the 48-hour monitoring period (kg)	-3.2 (-4.1, -2.3)	0.0006	-3.1 (-4.1, -2.1)	0.0021
Kerosene lamp use, average (hours)	-6.8 (-9.5, -4.1)	0.0099	-	-
Average number of people cooked for	-10.7 (-15.5, -6.1)	0.0175	-	-
Average time spent cooking a meal (min)	-0.4 (-0.6, -0.2)	0.0175	-	-
Number of cooking events	-3.3 (-5.7, -0.9)	0.1682	-	-

^1^Reference category: 3-stone fire; overall p-value for stove type p = 0.0051

^2^Mixed effects model, accounting for correlated response within household by follow up period

Although women were discouraged from using TSF during the monitoring period, 27% to 46% of women reported continued use of TSF along with ICS. Among households reporting exclusive use of ICS during the monitoring period (i.e., no stove stacking), overall larger reductions in PM_2.5_ were observed compared to households reporting continued use of a TSF during the monitoring period (Table 6). Among households using only ICS, statistically significant reductions in PM_2.5_ were observed for four ICS, while among stove-stacking households, significant reductions were observed only for two ICS. Among households using only ICS, statistically significant reductions in kitchen CO concentrations were observed for 3 ICS and in personal (woman) CO concentrations for 5 ICS, while among stove-stacking households, significant reductions were observed only for one ICS, although the small sample size limited our ability to assess statistical significance for comparisons among households reporting multiple stove use. The lowest mean PM_2.5_ concentrations (206μg/m^3^), mean kitchen CO (2.4 ppm), and mean personal CO (0.7 ppm) concentrations were observed in households using solely Prakti (i.e., no stove stacking reported).

**Table 6:** Gravimetric PM_2.5_ (μg/m^3^) and kitchen and personal (woman) CO real time (ppm) 48-hour concentration by stove type, stratified by reported multiple stove use.

	Households reporting multiple stove use					Households reporting use of only improved stove				
Stove type	N	Baseline^a^	Follow up^b^	Difference, mean (median, μg/m^3^)	p-value^c^	N	Baseline^a^	Follow up^b^	Difference, mean (median, μg/m3)	p-value^c^
Gravimetric kitchen PM_2.5_ (mg/m^3^), geometric mean										
Ecochula	13	605	549	100 (49.0)	0.6248	23	630	502	125 (392)	0.2950
Envirofit	10	709	527	-5.7 (293)	0.2665	25	585	355	391 (180)	**0.0098**
EcoZoom	10	705	861	-316 (50.5)	0.4044	27	577	412	266 (248)	**0.0435**
Philips	12	750	410	514 (349)	**0.0145**	23	541	281	275 (274)	**0.0043**
Prakti	18	865	751	-33.4 (289)	0.4728	21	422	206	249 (242)	**0.0018**
Rocket with TECA	13	870	402	433 (410)	**0.0042**^**d**^	22	446	349	86 (170)	0.2861^**e**^
CO real-time kitchen (ppm), geometric mean										
Ecochula	12	6.5	5.7	0.3 (0.5)	0.5612	22	6.9	5.2	2.4 (3.3)	0.1798
Envirofit	10	8.9	6.5	1.6 (2.1)	0.0641	24	6.0	4.3	4.1 (2.1)	**0.0211**
EcoZoom	10	6.9	9.6	-3.2(-1.0)	0.1478	27	6.5	5.8	0.9 (1.7)	0.5280
Philips	12	7.9	4.8	4.0 (3.8)	0.1353	23	5.9	3.3	2.1 (2.4)	**0.0292**
Prakti	18	8.9	9.4	-2.0 (0.1)	0.7730	20	5.0	2.4	3.2 (3.7)	**0.0026**
Rocket with TECA	13	8.9	5.2	5.0 (1.6)	**0.0228**	21	4.7	4.0	1.0 (1.1)	0.4398
CO real-time personal (ppm), geometric mean										
Ecochula	10	2.2	0.7	2.2 (1.5)	**0.0063**	21	2.7	1.1	1.4 (1.1)	**<0.0001**
Envirofit	9	2.0	1.1	1.3 (0.3)	0.0941	21	2.6	1.2	1.4 (1.4)	**0.0005**
EcoZoom	8	2.3	1.0	-0.4 (0.6)	0.0973	23	2.1	1.4	1.1 (0.7)	**0.0003**
Philips	10	2.4	1.5	-1.0 (1,0)	0.4510	19	2.0	0.9	1.5 (1.3)	**0.0009**
Prakti	13	2.4	1.4	0.9 (0.6)	0.1293	19	1.8	0.7	0.9 (0.9)	**0.0031**
Rocket with TECA	11	1.8	0.9	0.7 (0.9)	0.1407	20	2.6	1.9	0.8 (0.8)	0.1265

^a^ Baseline measurements in a setting of 3-stone fire use

^b^ Measurements in a setting of improved stove use

^c^ Paired t-test, assuming lognormal distribution

^d^Sign test, p-value = 0.0479

^e^Sign test, p-value = 0.0509

Stove type, use of only an improved stove (vs. stove stacking), and the amount of fuel used were the only independent predictors of 48-hour mean PM_2.5_ (μg/m^3^) in multivariable analysis. Percent reductions in mean PM_2.5_ compared to TSF, adjusting for multiple stove use, and amount of fuel used, ranged from 11.9% for households using Ecochula to 42.3% for Philips (Table 4). Use of an improved stove only vs. stove stacking was associated with a 29% reduction in mean PM_2.5_, while each additional kilogram of fuel consumed was associated with 3.0% increase in mean PM_2.5_

Stove type, exclusive use of an improved stove (vs. stove stacking), and amount of fuel used were the only independent predictors of 48-hour mean kitchen CO levels in multivariable analysis (Table 5). Percent changes in kitchen CO compared to TSF, adjusting for multiple stove use and amount of fuel used, ranged from 5.8% increase for households using EcoZoom to 34.5% reduction for Philips. Use of the improved stove only vs. stove stacking was associated with 28% reduction in mean CO. Each additional kilogram of fuel consumed was associated with 3.1% increase in mean CO, adjusting for multiple stove use and stove type.

### Factors associated with multiple stove use

Given that multiple stove use was an important and potentially modifiable predictor of mean kitchen PM_2.5_ concentration, we evaluated factors associated with multiple stove use using a multivariate linear regression model. Number of people cooked for, the average length of each cooking episode, number of meals prepared during the monitoring period, stove type used, age of cook, and socioeconomic status were examined and found not to be associated with multiple stove use.

### Qualitative findings

Analysis of information collected through structured interviews and focus groups indicated that the women liked ICS and found the stoves easy to use compared to the traditional TSF. Overall, women viewed ICS as more efficient, easier to light and retain heat, producing less smoke, and cooking faster. However, women did note that some of the ICS were not well-suited for cooking traditional dishes (EcoZoom and Prakti), had small combustion chambers that filled quickly with ash (EcoZoom), were slow to cook local food (Prakti and Rocket with TECA), or were difficult to use or light (Ecochula and EcoZoom).

During the final focus groups, women were asked to rank their 1^st^ and 2^nd^ choice ICS. Points were allocated per ranking: 2 points for 1^st^ choice and 1 point for 2^nd^ choice. There were clear preferences for specific ICS with Philips fan stove ranked as first and Ecochula as (last) sixth (Table 7). The Philips was associated with the largest percent reductions in mean PM_2.5_ and CO, the largest fuel savings (56% less fuel consumed compared to TSF), though this stove was not associated with the least amount of stove stacking (Table 2).

**Table 7:** Main qualitative and quantitative findings by stove type.

Source of information	Reduction in 48-hr kitchen measurement, difference (% change^1^)		Fuel consumption	Time activity diary		Qualitative interviews and FGDs			
Stove type	PM_2.5_ (μg/m^3^)	CO (ppm)	Fuel used, kg (% reduction)	ICS use during monitoring N (%)	Multiple stove use (%)	Overall stove rank order (range 1–6)^2^	Stove characteristics (rank order) women liked	Stove characteristic women disliked	Selected quotes illustrating usability/acceptability
TSF	-	-	12.0	-	-	-			
Ecochula	116 (11.9)	1.7 (14.1) ^3^	7.5 (38%)^3^	33 (92%)	36	6	Fuel efficiency (2), Cooking speed (2), Suitable for cooking traditional foods (3),Visually appealing (3)	Requires pulling out of stove to add fuel; cooks food unevenly; concerns around durability and maintenance	*“I like it because*.* *.* *.*it consumes less fuel*, *…uses charcoal*, *…can also use cow dung and …I don’t need to adjust the flame and it doesn’t give me hard time of adjusting the flame …and it cooks food so well*.*”;“It is hard because I use a wok when cooking ugali …you were cooking and the fuel gets finished is when you want to pull it out … have served ugali which is not well cooked*.*”*
Envirofit	277 (36.1)^3^	3.4 (27.9) ^3^	9.3 (23%)^3^	35 (100%)	29	3	Suitable for cooking traditional food (2), Even heat without flare ups (3), Cooking speed (3), Cooking pots fit well (3)	Small burning chamber; requires constant supervision	“*…is good for me because I put firewood and that stand holds for me the fuel and it burns so well … even if you put a lot of fuel …and it cooks faster then …and I can carry it and cook with it in the compound or in the other house…*.”; “*The problem I saw …the pot rest was so small and also the combustion chamber so that if you have a big family you cannot cook with it*.”; “*I cannot do anything until I am done using it*, *after I am done…is when I go and do my work outside”*
EcoZoom	109 (14.9) ^3^	-0.2 (-5.8)	8.5 (29%)^3^	37 (100%)	27	4	Even heat without flare ups (1), Fuel efficiency (3)	Mixed views on cooking speed;-some women note not good for cooking local dishes; small burning chamber; difficult to teach others to use; difficulties with pot stability and heat adjustment	*“…stove was good*, *I cooked ugali… it was easy to use*, *the fire was lighting well and it could reach at the bottom of the cooking pan*”, “*Cooking with it was difficult*, *when you place the cooking pot on the top it shakes*, *when you adjust the fire sometimes it goes off…”*
Philips	357 (42.3) ^3^	2.7 (34.5) ^3^	5.3 (56%)^3^	33 (92%)	34	1	Comfortable (1);-Cooking speed (1); Fuel efficiency (1); Reduces smoke (2); Visually appealing (2); Cooking pots fit well (2)	Not good for dishes that require slow cooking; requires small pieces of wood; requires constant supervision; small or instable pot rest; concerns about durability, maintenance, burns	“*I like it because it cooks so well*.* *.* *.*lighting it is also easy such that even if you teach a child she can light it*. *…I can cook very fast …it also consumes less fuel*. *During harvesting season I can use the maize cob as fuel and I just keep the firewood*.”, “*The problem it has is … the charging knob gets spoiled so there is no way you can repair it … and there is no way you can use it when it is not charged*. *…when the battery get spoiled*, *there is no way you can get that battery…”*
Prakti	118 (41.1) ^3^	0.7 (33.5) ^3^	9.5 (21%)^3^	39 (100%)	46	5	Reduces smoke (1); Visually appealing (1); Even heat without flare ups (2); Two burners (can cook and warm food at the same time)	Slow cooking speed; hard to cook traditional dishes; small pot rest; concerns of burns from chimney	“*I like it because there isn’t smoke produced in the house since its chimney is directed outside and so when I am cooking I put food on this side and on the other side I put another thing and … you can cook two things at the same time so fast and also it doesn’t consume a lot of fuel”*, *“…the two pot rests were made …that if you place the bigger pot on one side then the rest cannot fit the other side …but when you use [only] one side the smoke now comes in the house*.* *.* *.*”*
Rocket with TECA	215 (32.7) ^3^	2.5 (24.6) ^3^	8.2 (32%)^3^	35 (100%)	37	2	Suitable for cooking traditional food (1); Cooking pots fit well (1); Comfortable (2)	Mixed opinions on ease of use; slow cooking speed; concerns about TECA fan (durability, maintenance)	“*…you don’t need to hold pot*. *It is stable and the pot does not move from place to place*”, “*I was told that when you put the firewood then the machine would fan the fire*. *I waited …but I did not see it fanning the fire*. *When I pushed the firewood*, *I also had some fear that it might touch the metals inside the stove*. *So it was really hard for me to use*.”

^1^Estimated using multivariable mixed effects model, accounting for correlated response within household by follow up period

^2^During six focus groups, 39 women ranked their 1^st^ and 2^nd^ stove choice. Points were allocated per ranking (2 points for 1^st^ choice and 1 point for 2^nd^ choice)

^3^ Statistically significant, p<0.05

Women reported they liked Philips because of its cooking speed (cooks fastest), ease of use, portability, reduction in indoor smoke and fuel consumption. The concerns women expressed about Philips included the need to prepare small pieces of fuel, the need for constant supervision to maintain fire, instability of cooking pots, and the stove durability and availability of parts to maintain functionality (solar charger, battery). The study stove ranked the lowest by users (Ecochula) was associated with the lowest percent reduction in PM_2.5_ and the second to lowest reduction in CO, though it ranked second in fuel savings.

## Discussion

Results of our study evaluating six improved biomass stoves in rural Western Kenyan households demonstrated that in a setting of everyday use these stoves reduce indoor air pollutants and are acceptable to local women. To our knowledge, this is the first study to evaluate several improved stoves in the same set of households, to simultaneously measure the impact of short term stove use on personal and kitchen levels of PM_2.5_ and CO, and to assess the acceptability of these stoves to users through structured interviews and focus group discussions.

The baseline levels of kitchen PM_2.5_ observed in our study households in Kenya are comparable to those reported in studies in Mexico[14] and India[15] but higher than in Guatemala[16], and are more than 20 times higher than WHO guideline values.[7] While modest reductions in levels of PM_2.5_ were observed for all study stoves compared to the traditional TSF, only four of six stoves generated statistically significant reductions. Studies evaluating the effects of improved cookstove introductions in Guatemala[16, 17] and Mexico[14] demonstrated significant reductions in kitchen PM_2.5_ and of larger magnitude compared to reductions observed in our study, while the study in India did not show significant reductions. An earlier study in the villages of the same district in Kenya found that the households using the locally made upesi jiko stove observed 13% lower kitchen PM_2.5_ levels than households using a TSF, however this difference was not statistically significant despite reports by study participants of visible smoke reductions in households using upesi jikos (CDC unpublished data). Despite achieving percent reductions in mean kitchen 48-hour PM_2.5_ levels of a larger magnitude in our study (ranging from 18% to 45%), none of the ICS achieved the WHO guideline level for annual average kitchen PM_2.5_ of 10μg/m^3^, nor the intermediate target of 35μg/m^3^.[7]

Carbon monoxide is simpler to measure in field settings than particulate matter; the use of relatively inexpensive and lightweight devices allowed us to measure kitchen and personal levels simultaneously. The results of kitchen level CO measurements show reductions in mean 48-hour CO associated with the use of ICS, and these findings are consistent with the reductions observed in kitchen PM_2.5_ by stove type and with the use of improved stoves exclusively. Use of CO as a marker for PM_2.5_ has been suggested in previous studies. However, even though a moderately strong relationship between kitchen CO and PM_2.5_ was demonstrated in our study population, this relationship may not be extrapolated to other settings with different cooking behaviors, fuel and stove types. Interpretation of personal CO results is further complicated by women’s behaviors. Most participating women reported having duties other than cooking for their households which required them to leave the house for extended periods of time. We were not able to assess whether these behaviors changed between the monitoring periods. In addition, assessment of adherence to personal CO monitor use was based on self-report and periodic visits made during the monitoring period by the field officers. Nevertheless, our results show significant reductions in levels of personal CO for women during use of all ICS as compared to the baseline.

All ICS in this study were first evaluated in a controlled laboratory setting by USEPA and demonstrated >50% reduction in PM_2.5_ emissions compared to TSF.[8] Several factors likely limited the reduction observed in kitchen PM_2.5_ and CO during everyday use. Traditional TSF was used during the monitoring period along with the improved stove in 27% to 46% of households, depending on the ICS type evaluated. In our study, the largest reductions in kitchen mean 48-hour PM_2.5_ and CO were observed among households using ICS only and exclusive use of ICS was an independent predictor of and was associated with an almost 30% reduction in mean kitchen levels of both PM_2.5_ and CO. Continued use of traditional stoves alongside an improved stove has been reported in other studies.[14, 16] Among women’s explanations for multiple stove use are convenience of having an additional stove, preference of TSF for certain local dishes or for accommodating large pots, or special family/community occasions requiring additional cooking capacity. One of our study stoves (Prakti) had a second burner, and women reported to like this characteristic that allowed them to “…*cook two things at the same time so fast…*” However, women also reported that the two burners on this stove were not functionally equivalent, and households with this stove also reported using the TSF more often during the monitoring period. While we were not able to identify any modifiable predictors of multiple stove use, qualitative data suggest that addressing stove design limitations, such as having stoves with two functional burners, ability to accommodate large pots, and capacity to simmer food slowly will help meet cooking needs of users. Qualitative data suggest that women may view ICS as an additional household tool used for cooking rather than a replacement stove, and future studies should take this into account when selecting an acceptable intervention.

Kerosene lamps likely contributed to high levels of kitchen PM_2.5_ observed in our study households. Study participants reported using kerosene lamps on average 6 hours per day indoors. The duration of lamp use did not vary by follow up period or by stove type used and was not an independent predictor of kitchen PM_2.5_ or CO level. We were not able to measure the contribution of the kerosene lamp to 48-hour mean kitchen PM_2.5_ directly, nor were we able to adjust the analysis for the type of kerosene lamp used in each household. However, duration of kerosene lamp use was positively associated with kitchen levels of PM_2.5_ and CO. Studies have demonstrated that use of the crudest “simple-wick” kerosene lamps contributes to indoor levels of PM_2.5_ that are an order of magnitude greater than WHO air quality guidelines.[18, 19] Increasing availability of light emitting diode (LED) or solar powered lamps can help reduce contribution from kerosene lamps to indoor pollution.

Our study was not statistically powered to make direct comparisons among the improved stoves in their effectiveness to reduce indoor air pollution. However, during in-depth interviews and focus group discussions, women’s stove preferences clearly emerged. We outlined women’s ranking of, and views on, the stoves including a number of stove characteristics that the women valued as well as those that made the stoves less popular. Although we are not able to directly link women’s preferences toward improved stoves to actual stove use and performance, it may be reasonable to assume that certain stove characteristics viewed by users as favorable are likely to improve the adherence to stove use. Consequently, we could expect that the stoves women ranked the highest overall and in terms of certain characteristics will be used exclusively more often and will achieve the highest PM_2.5_ reductions. While our data supports part of this assumption, given that the stove ranked the highest overall and based on several characteristics (Phillips) was also the one associated with the largest reductions in kitchen PM_2.5_ and CO, the same Phillips stove did not have the highest level of exclusive use. Likewise, while the stove ranked the lowest (Ecochula) was associated with the lowest percent reductions in PM_2.5_, this stove was not associated with the highest proportion of multiple stove use. We should also note that stoves ranked as second or third were also associated with similar reductions in PM_2.5_, and this ranking does not necessarily imply that women disliked the stoves as compared to the TSF but rather demonstrates how they ranked the stoves relative to each other. A number of factors we identified related to stove preferences that may impact on stove use concur with the literature on barriers and facilitators to scaling up of improved cookstoves.[20] Many of these factors could be taken into account and addressed by the stove manufacturers.

Exposure-response analysis from the randomized controlled trial in Guatemala suggests that achieving exposure reduction needed for prevention of child pneumonia may require use of clean fuels or biomass stoves with cleaner combustion. [5] Recently developed integrated exposure-response functions for five disease outcomes suggests that for the ALRI outcome the shape of the curve is steeper at lower levels of PM_2.5_ and flattens out at levels higher than 300 μg/ml.[6] Therefore, the relatively modest reductions in kitchen PM_2.5_ observed in our study, would translate into small reduction in estimated relative risk for the ICS compared to TSF given high levels of exposure observed at baseline. At lower baseline levels of exposure, a similar magnitude of reduction in PM_2.5_ is expected to result in larger effect on ALRI risk. Based on this model prediction, exposure has to reach the level at or below the WHO intermediate target level of 35μg/m^3^ for PM_2.5_ to lead to substantial ALRI risk reduction. These findings demonstrate the need for more effective solid fuel interventions, clean fuels, and more exclusive use of these, to reduce high baseline indoor levels further and lead to lower personal exposures and a larger health impact.

The traditional TSFs are easy to assemble and could have been built and taken apart anytime during the monitoring period. In this analysis, we used time-activity diaries as a source of stove use data, and women may have underreported TSF use which would have underestimated the measured impact of improved stoves. Even though we collected data on stove use using temperature data loggers, in about 25% of the study days these measurements were missing due to the operational constraints or malfunctioning of temperature data loggers.[12] As a result, stove use data collected through temperature data loggers in this analysis would have limited our sample size. In addition, the short term follow up with each improved stove does not allow for continuous education on stove use over time, which may lead to a greater familiarity with and in turn adherence to stove use. Introduction of improved stoves into households requires a significant behavioral change for women, as it often involves changing the cooking position, chopping wood into smaller pieces, and the need for closer monitoring of the cooking process to ensure continuous combustion. The impact of the stoves on indoor air quality may improve with longer use of acceptable stoves or may worsen if the stoves are no longer used or lose functionality due to required maintenance. Therefore, longer-term impact of improved stoves, for example over a 12 month period, should also be evaluated.

We limited the influence of household level factors that could be related to stove use or household air pollution by conducting measurements at baseline and after installation of each improved stove within the same households. Although changes in daily activities could still have influenced the findings, the behaviors measured during each follow up period (e.g., number of people cooked for, average time spent cooking a meal, number of cooking events) did not differ by follow up period.

The results of this study have implications for future health impact studies seeking to identify an effective and acceptable intervention that can demonstrate health benefits. Evaluation of stove acceptability by local users is essential during the design phase as well as prior to use in intervention trials or large scale dissemination. All the study stoves performed better in a controlled laboratory setting, and our field evaluation demonstrated that women’s cooking patterns and behaviors clearly influence ICS performance. Unless the stoves meet the needs and priorities of target users, biomass stoves are unlikely to make an impact on household air pollution. When designing ICS to improve household air pollution, the stove manufacturers should take into account the needs and preferences of users. In addition, more rigorous communication on proper stove use and education on health benefits of improved air quality to influence behavior change and promote adherence to stove use can help maximize benefits of ICS. A more thorough evaluation of other potential sources of indoor air pollution in households (e.g., kerosene lamps) is also needed. Future studies should consider a package of interventions, such as multiple improved stoves or improved stoves with multiple burners and clean sources of lighting to improve indoor air quality.

This study documents the reductions of household air pollution from several improved biomass stoves compared to levels observed in a setting of traditional TSF in rural Kenyan homes. Achieving clean biomass requires understanding and influencing a complex mix of factors such as stove design, performance in the field, users’ needs and preferences, fuel type used and moisture content, household ventilation, and other sources of household air pollution. We have demonstrated that several biomass stoves have the potential to improve indoor air quality but achieving their maximal potential requires adherence to more exclusive use, as well as elimination of other sources of household air pollution, principally kerosene lamps. The levels observed in a setting of improved stove use in our study, however, are still considerably higher than indoor air quality standards and consequently risk reductions for a range of child and adult health outcomes are limited. We were unable to demonstrate a link between stove acceptability to stove use and performance but have identified stove characteristics women liked and, therefore, likely promoted use of the improved stove. Although the improved stoves were largely acceptable to local women, all six stoves were reported to have some limitations or concerns, and addressing these could lead to more exclusive and sustained use. Further research is needed on stove use patterns and local user preferences to determine whether useful additional benefits to health can be achieved through the better use of biomass stoves and improvements in the technology. Even if further such benefits can be obtained, this study does suggest that clean fuels will be required in order to meet WHO air quality guidelines for PM_2.5_ in homes. In poor rural populations such as this one, it is challenging to ensure affordable and secure supply of clean fuels; policy makers should therefore consider addressing both enhancing solid fuel technology and support for its best use, as well as working to make clean fuels available.

## Supporting Information

S1 File Appendices 1 and 2. (DOCX)

S2 File Data collection instruments. (DOCX)

S3 File Time activity diary. (PDF)

S4 File Data tables. (PDF)

S5 File IRB approval ERC. (PDF)

S6 File IRB approval CDC. (DOCX)
