# Supplementary material for: Effectiveness of Six Improved Cookstoves in Reducing Household Air Pollution and Their Acceptability in Rural Western Kenya
Source: PLoS One. 2016 Nov 15;11(11):e0165529. doi: 10.1371/journal.pone.0165529 (PMC5112915; doi:10.1371/journal.pone.0165529)
Supplement: S1 File — (DOCX) [file pone.0165529.s001.docx]

**Appendix 1.** Seven cookstoves assessed in the study

3 stone fire (TCS) Eco Chula EcoZoom Envirofit Philips Pratki RTI TECA


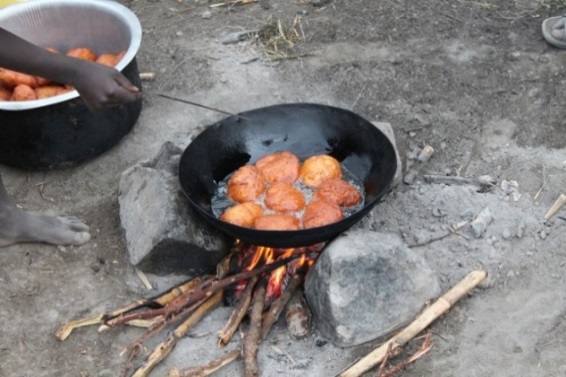

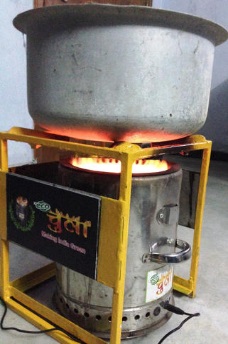

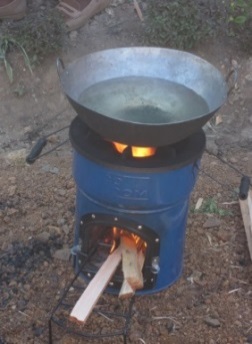

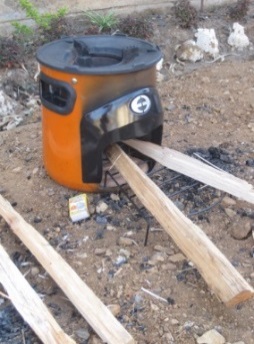

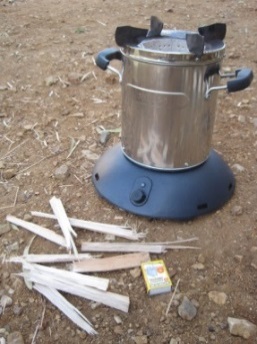

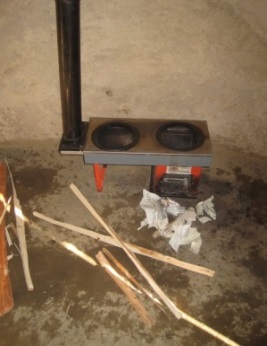

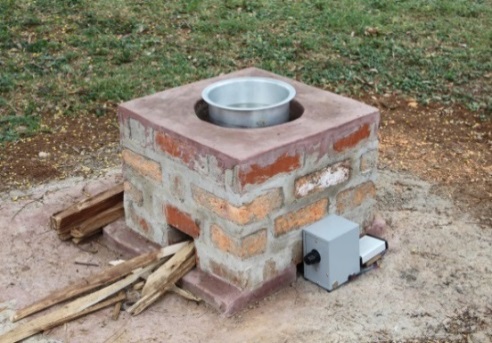


**Appendix 2.** Quality Assurance

**Instrument calibration**

For the gravimetric PM_2.5,_ the target flow rate was 1.5 L/min. Pre- and post-calibrations were made by either a rotameter (AALBORG, Orangeburg, NY, USA) or a Dry Cal DC-Lite (Bios International, Butler, NJ, USA) in the field. The GasBadge was calibrated in Berkeley Air’s laboratory (Berkeley Air Monitoring Group, Berkeley, CA, US) with 20 ppm CO span gas and zeroed in the field prior to each deployment.

**Filter weighing**

At the beginning of each filter weighing session, a 100 mg calibration weight and three lab blanks were weighed.​​ ​Zeros were checked after every mass measurement. After every tenth sample, the balance’s reproducibility​ ​​was checked by reweighing the first filter in the previous batch of 10. All filters​ ​​were weighed twice.​ If​ ​​the first and second mass measurements differed by >​5 µg (<1% of filters), filters​​ ​were weighed a third time.​ At the end of the session, the three lab blanks were re-weighed to assess drift.​ Field blanks (approximately 5% of the total number of samples) were collected and used for quality assurance.
