# Supplementary material for: Effectiveness of Six Improved Cookstoves in Reducing Household Air Pollution and Their Acceptability in Rural Western Kenya
Source: PLoS One. 2016 Nov 15;11(11):e0165529. doi: 10.1371/journal.pone.0165529 (PMC5112915; doi:10.1371/journal.pone.0165529)
Supplement: S2 File — (DOCX) [file pone.0165529.s002.docx]

**EVALUATION OF ACCEPTABILITY AND SUSTAINABILITY of improved stoves AND THEIR IMPACT ON INDOOR air quality and CHILD HeALTH in rural western Kenya**

|  |  |
| --- | --- |

General background, biomarker, and stove-related air pollution exposure assessment [English version]

Baseline Questionnaire

(To be administered prior to the 48-hr air sampling)

| **A: IDENTIFICATION INFORMATION** | | |
| --- | --- | --- |
| **A1.1** | Study household ID number |  |
| **A2.1** | Neighbourhood |  |
| **A3.1** | Name [ID] of Interviewer (NOT INTERVIEWEE) |  |
| **A4.1** | Date of interview (DD/MM/YYYY) | __ __/ __ __ / __ __ __ **DATE**__ |
| **A5.1** | Start time of interview (Use 24 hour clock) | _**TIME**_ __ / __ __ |

| **SETTING UP EQUIPMENT FOR PERSONAL MONITORING**  Explain that we will set up kitchen monitor and the monitor for the woman, and the tube on the child, after asking the questions. |
| --- |

| **B: FAMILY INFORMATION** | | | |
| --- | --- | --- | --- |
| **B1** | How old are you (years)? | | **NUM** |
| **B2** | Marital status: Are you ….. | 1 = Married  2 = Single mother  3 = Separated  4 = Divorced  5 = Widowed | **NUM** |
| **B3** | How many people (in the age groups below) usually live in this house? | |  |
| **B3.1** | Males | Less than 5 years: |  |
| **B3.2** |  | 5-14 years |  |
| **B3.3** |  | 15 years and older |  |
| **B3.4** | Females (including yourself) | Less than 5 years: |  |
| **B3.5** |  | 5-14 years |  |
| **B3.6** |  | 15 years and older |  |
| **B4** | How many people in total usually live in this house? [Check] | |  |
| **B5.1** | What is the age of your youngest child? | Years |  |
| **B5.2** |  | Months |  |
| **B6** | What is the sex of the youngest child? | 1 = Male  2 =Female |  |

| **C: SOCIO-ECONOMIC CIRCUMSTANCES OF THE HOUSEHOLD** | | | | | | | | | |
| --- | --- | --- | --- | --- | --- | --- | --- | --- | --- |
| **EDUCATION** | | | | | | | | | |
| **C1.1** | If you attended school, for how many years did you attend?  *(if did not attend school, enter ‘0’ (zero))* | | | | | | | |  |
| **C1.2** | What level of education did you reach? | | | | | 1 = No formal education  2 = Primary  3 = Secondary  4 = Higher | | |  |
| **C2.1** | Are you comfortable with reading? | | | | | 1 = Yes 2 = No | | |  |
| **C2.2** | Are you comfortable with writing? | | | | | 1 = Yes 2 = No | | |  |
| **C3.1** | If your husband attended school, for how many years did he attend?  *(if he did not or not applicable, put '0'(zero) and go to C6.1)* | | | | |  | | |  |
| **C3.2** | What level of education did your husband reach? | | | | | 1 = No formal education  2 = Primary  3 = Secondary  4 = Higher | | |  |
| **C4.1** | Is your husband comfortable with reading? | | | | | 1 = Yes 2 = No | | |  |
| **C4.2** | Is your husband comfortable with writing? | | | | | 1 = Yes 2 = No | | |  |
| **EMPLOYMENT** | | | | | | | | | |
|  | **1** | Farms his/her own land (whether that land is owned or rented) | **6** | | Craftsperson (tailor, carpenter, seamstress etc.) | | | | |
|  | **2** | Day labourer (farming another person’s land, house-building etc. ) | **7** | | Runs the household / Cares for family | | | | |
|  | **3** | Government employee (doctor, nurse, police, teacher etc. ) | **8** | | Retired | | | | |
|  | **4** | Employee in a business (Factory worker, works in a shop) | **9** | | Other type of job | | | | |
|  | **5** | Has own business (owns a shop etc.) | **10** | | Currently unemployed | | | | |
| **C5.1** | What is your husband’s main occupation / job? *Use list above* | | | | | | First |  | |
| **C5.2** | If your husband has a second job, what is it? | | | | | | Second |  | |
| **C5.3** | If his occupation is not listed, please describe here | | | **English translation here please** | | | | | |
| **C6.1** | What is your main occupation or job? *Use list above* | | | | | | First |  | |
| **C6.2** | If you have another occupation, what is it? | | | | | | Second |  | |
| **C6.3** | If your secondary occupation is not listed, please describe here | | | **English translation here please** | | | | | |

| **WATER AND SANITATION** | | | | | | | | | | | | | |
| --- | --- | --- | --- | --- | --- | --- | --- | --- | --- | --- | --- | --- | --- |
| **C7.1** | Do you have access to drinking water in your house? | | | 1 = Yes  2 = No | | | | | | |  | | |
| **C7.2** | Where do you obtain most of your water for your household needs? | | | 1 = Piped in home  2 = Pump (deep well)  3 = Well (pit with bucket)  4 = Communal standpipe  5 = Collect from river | | | | | | |  | | |
| **C8** | Do you have a septic tank toilet inside the house or a latrine in the yard? | | | 1= Inside your house  2 = In the yard  3= None | | | | | | |  | | |
| **POSSESSIONS & INCOME** | | | | | | | | | | | | | |
| C9 | Do you own/have any of the following? | |  | | | | | | | | | | |
| **C9.1** |  |  | Motor-bike | | | | | | 1 = Yes 2= No | | |  | |
| **C9.2** |  |  | Refrigerator | | | | | | 1 = Yes 2= No | | |  | |
| **C9.3** |  |  | Electricity connection | | | | | | 1 = Yes 2= No | | |  | |
| **C9.4** |  |  | Access to electricity generator | | | | | | 1 = Yes 2= No | | |  | |
| **C9.5** |  |  | Radio | | | | | | 1 = Yes 2= No | | |  | |
| **C9.6** |  |  | Hi-Fi / CD-player | | | | | | 1 = Yes 2= No | | |  | |
| **C9.7** |  |  | TV | | | | | | 1 = Yes 2= No | | |  | |
| **C9.8** |  |  | Bicycle | | | | | | 1 = Yes 2= No | | |  | |
| **C9.9** |  |  | Car/truck | | | | | | 1 = Yes 2= No | | |  | |
| **C9.10** |  |  | Cell phone | | | | | | 1 = Yes 2= No | | |  | |
| **C9.11** |  |  | Shower / bath within house | | | | | | 1 = Yes 2= No | | |  | |
| **C9.12** |  |  | Cow | | | | | | 1 = Yes 2= No | | |  | |
| **C10** | Would you mind us asking a question about your family’s weekly income | | | | | | | | 1 = Yes (go to D1)  2 = No | | |  | |
| **C11** | About how much money do you have available for household purchases each week? | | | | | | | |  | | | | |
| **C12** | Do you feel that this amount of money is … | | | | | 1 = Enough to buy everything needed  2 = Not quite enough  3 = Much too little | | | | | |  | |
| TOBACCO SMOKING | | | | | | | | | | | | | |
| **C13.1** | | Do you smoke cigarettes, or have you ever smoked cigarettes for one year or more? | | | | | 1 = Yes  2 = No (Go to C14) | | | | | |  |
| **C13.2** | | Have you:  1 = continued to smoke (that is, a current smoker)?  2 = given up smoking altogether, within the last 4 weeks?  3 = have stopped completely for more than 4 weeks? | | | | | | | | | | |  |
| **C13.3** | | How many **cigarettes** do (did) you smoke each day, on average? | | | | | | | | | | |  |
| **C14** | | Does anyone else in your household smoke cigarettes? | | | | | 1 = Yes  2 = No (Go to Question L12.1) | | | | | |  |
| **C14.2** | | If Yes, do they smoke in the house, or only outside the house? | | | 1 = Inside (at least sometimes)  2 = Outside only (Go to Question L12.1) | | | | | | | |  |
| **C14.3** | | How many people smoke cigarettes inside the house? | | | | | | | | Number of people | | |  |
| **C14.4** | | About how many cigarettes in total are smoked each day **in your house** by other people? | | | | | | Number of cigarettes per day | | | | |  |

| **D: FUELS USED FOR COOKING** | | | | | | | | | | | | |
| --- | --- | --- | --- | --- | --- | --- | --- | --- | --- | --- | --- | --- |
| **FUELS LIST** | | | | | | | | | | | | |
|  | | **1** | Ethanol | **2** | Kerosene | | | | **3** | | Bottled gas (LPG) | |
|  |  | **4** | Wood | **5** | Agri-Residues | | | | **6** | | Charcoal | |
|  |  | **7** | Sawdust | **8** | Animal Dung | | | | **9** | | Electricity | |
|  |  | **10** | Local generator | **11** | Battery | | | | **12** | | Candles | |
|  |  | **13** | Other |  | | | | | | | | |
| D1 | What is your **main** cooking fuel … *(please use the list above)*  …..in the wet season  …..in the dry season | | | | | | | | |  | | |
| **D1.1** |  |  |  |  |  |  |  |  |  | Wet | |  |
| **D1.2** |  |  |  |  |  |  |  |  |  | Dry | |  |
| **D1.3** | If any non-listed **main fuel** (wet season), describe here: | | | | | | |  | | | | |
| **D1.4** | If any non-listed **main fuel** (dry season), state here: | | | | | | |  | | | | |
| **D2** | What is your **secondary** cooking fuel, if used…  …..in the wet season  …..in the dry season | | | | | | | | |  | | |
| **D2.1** |  |  |  |  |  |  |  |  |  | Wet | |  |
| **D2.2** |  |  |  |  |  |  |  |  |  | Dry | |  |
| **D2.3** | If any non-listed **secondary** fuel (wet season), state here: | | | | | **English translations here please** | | | | | | |
| **D2.4** | If any non-listed secondary fuel (dry season), state here: | | | | |  | | | | | | |
| **D2.5** | What proportion of the total fuel is the secondary fuel? | | | | | | 1= about half  2 = about quarter  3 = Very small amount | | | | |  |
|  | ***IN THIS NEXT SECTION, CHECK WHICH FUELS THE INTERVIEWEE USES, AND ASK QUESTIONS ONLY ABOUT THOSE FUELS*** | | | | | | | | | | | |

|  | **CHARCOAL USERS ONLY** | | | | |
| --- | --- | --- | --- | --- | --- |
| **D3.1** | **CHARCOAL USERS**  **Complete this if interviewee answered ‘6’ to any of these questions D1.1 D1.2**  **D2.1 D2.2**  *You may need to weigh one or two bags of charcoal if the woman does not know their weight* | Confirm: does the respondent use any charcoal for cooking? | | 1 = Yes  2 = No |  |
| **D3.2** |  | Do you buy small or large bags of charcoal? | | 1 = Small  2 = Large (Go to D3.5)  3 = Both |  |
| **D3.3** |  | **In the wet season** | About how many **small** bags of charcoal do you need to buy each week for the stove in the wet season? | |  |
| **D3.4** |  |  | How much does **one** **small** bag weigh? | |  |
| **D3.5** |  |  | How much does **one** **small** bag cost? | |  |
| **D3.6** |  |  | About how many **large** bags of charcoal do you need to buy each week for the stove in the wet season? | |  |
| **D3.7** |  |  | How much does **one large** bag weigh? | |  |
| **D3.8** |  |  | How much does **one large** bag cost? | |  |
| **D3.9** |  |  | About how much in total do you spend on charcoal each week in the wet season? | |  |
| **D3.10** |  | **In the dry season** | About how many **small** bags of charcoal do you need to buy each week for the stove in the dry season?  *Only ask if small bags are used* | |  |
| **D3.11** |  |  | About how many **large** bags of charcoal do you need to buy each week for the stove in the dry season?  *Only ask if large bags are used* | |  |
| **D3.12** |  |  | About how much in total do you spend on charcoal each week in the dry season? | |  |

|  | **WOOD & RESIDUE USERS ONLY** | | | | | | | | | | |
| --- | --- | --- | --- | --- | --- | --- | --- | --- | --- | --- | --- |
| **D4.1** | **WOOD & RESIDUE USERS**  **Complete this if interviewee answered ‘4’ or ‘5’ or ‘7’ to any of these questions D1.1**  **D1.2**  **D2.1**  **D2.2**  **WOOD & RESIDUE USERS**  **continued**  **WOOD & RESIDUE USERS**  **continued** | **Confirm: does the respondent use any wood or residues for cooking?** | | | | | 1 = Yes  2 = No | | | |  |
| **D4.2** |  | **In the wet season** | Usually, **in the wet season**, how much of the wood or residues you use is bought? | | | | 1 = All bought  2 = Most bought  3 = About half bought  4 = Most collected  5 = All collected (Go to D4.7) | | | |  |
| **D4.3** |  |  | If bought: amount bought per week **in the wet season** | | Number of bundles of wood | | | | | |  |
| **D4.4** |  |  |  |  | Weight of bundle bought wood (kg) | | | | | |  |
| **D4.5** |  |  |  |  | Average cost per week for all the bought wood or residues | | | | | |  |
|  |  |  | ***Ask only if any wood or residues are gathered*** | | | | | | | |  |
| **D4.6** |  |  | Number of bundles of wood or residues gathered per week **in the wet season** | | | | | | | |  |
| **D4.7** |  |  | Weight of one bundle of gathered wood or residue | | | | | | | |  |
| **D4.8** |  |  | Who collects the wood or residues? | | Yourself | | | | | 1 = Yes 2= No |  |
| **D4.9** |  |  |  |  | Husband | | | | | 1 = Yes 2= No |  |
| **D4.10** |  |  |  |  | Female children | | | | | 1 = Yes 2= No |  |
| **D4.11** |  |  |  |  | Male children | | | | | 1 = Yes 2= No |  |
| **D4.12** |  |  | How long does each trip take, on average (hours) | | | | | | | |  |
| **D4.13** |  |  | How many adults go out on each trip, on average | | | | | | | |  |
| **D4.14** |  |  | How many children go out on each trip, on average | | | | | | | |  |
| **D4.15** |  | **In the dry season**  **Dry season contd** | Usually, **in the dry season**, how much of the wood or residues you use is bought? | | | | | 1 = All bought  2 = Most bought  3 = About half bought  4 = Most collected  5 = All collected *(go to D4.22* | | |  |
| **D4.16** |  |  | If bought: amount bought per week **in the dry season** | | | Number of bundles of wood | | | | |  |
| **D4.17** |  |  |  |  |  | Weight of bundle bought wood (kg) | | | | |  |
| **D4.18** |  |  |  |  |  | Average cost per week for all the bought wood or residues | | | | |  |
|  |  |  | ***Ask only if any wood or residues are gathered:*** | | | | | | | |  |
| **D4.19** |  |  | Number of bundles wood/residues gathered per week **in the dry season** | | | | | | | | **NUM** |
| **D4.20** |  |  | Weight bundle gathered wood or residues (kg) | | | | | | | |  |
| **D4.21** |  |  | Who collects the wood or residues? | Yourself | | | | | 1 = Yes 2= No | |  |
| **D4.22** |  |  |  | Husband | | | | | 1 = Yes 2= No | |  |
| **D4.23** |  |  |  | Female children | | | | | 1 = Yes 2= No | |  |
| **D4.24** |  |  |  | Male children | | | | | 1 = Yes 2= No | |  |
| **D4.25** |  |  | How long does each trip take, on average (hours) | | | | | | | |  |
| **D4.26** |  |  | How many adults go out on each trip, on average | | | | | | | |  |
| **D4.27** |  |  | How many children go out on each trip, on average | | | | | | | |  |

|  | **BOTTLED GAS (LPG) USERS ONLY** | | | | |
| --- | --- | --- | --- | --- | --- |
| **D5.1** | **LPG USERS**  **Complete this if interviewee answered ‘3’ to any of these questions D1.1 D1.2**  **D2.1 D2.2** | Confirm: does the respondent use any LPG for cooking? | | 1 = Yes  2 = No |  |
| **D5.2** |  | What size bottles do you buy? (size in kg) | | |  |
| **D5.3** |  | How much does one bottle cost? | | |  |
| **D5.4** |  | In the **wet season** | How long does one bottle last? (days) | |  |
| **D5.5** |  | In the **dry season** | How long does one bottle last? (days) | |  |

|  | **KEROSENE USERS ONLY** | | | | |
| --- | --- | --- | --- | --- | --- |
|  | *Find out how it is bought, and work out number of mls of kerosene (eg if it is in a bottle, find out how much liquid can be contained in the bottle and find out the number of bottles used each week)* | | | | |
| **D6.1** | **KEROSENE USERS**  **Complete this if interviewee answered ‘2’ to any of these questions D1.1, D1.2**  **D2.1, D2.2** | **Confirm: does the respondent use any Kerosene for cooking?** | | 1 = Yes  2 = No |  |
| **D6.2** |  | In the **wet season** | How much kerosene do you buy each week? (mls) | | ml |
| **D6.3** |  |  | About how much do you spend each week on kerosene | |  |
| **D6.4** |  | In the dry season | How much kerosene do you buy each week? (mls) | | ml |
| **D6.5** |  |  | About how much do you spend each week on kerosene | |  |

|  | **ELECTRICITY USERS ONLY** | | | | |
| --- | --- | --- | --- | --- | --- |
| **D7.1** | **ELECTRICITY USERS**  **Complete this if interviewee answered ‘9’ or ‘10’ to any of these questions D1.1, D1.2**  **D2.1, D2.2** | **Confirm: does the respondent use any electricity for cooking?** | | 1 = Yes  2 = No |  |
| **D7.2** |  | How do you pay for your electricity | 1 = fixed price per day  2 = price per unit (eg kilowatt-hr)  3 = Other | |  |
| **D7.3** |  | What is the price of electricity per unit? *Insert units for both price and type of unit (eg Ar / kw-hour)* | | |  |
| **D7.4** |  | In the **wet season** | How much do you pay for your electricity per week | |  |
| **D7.5** |  | In the **dry season** | How much do you pay for your electricity per week | |  |

| **E: FUEL PREFERENCES** | | | | |
| --- | --- | --- | --- | --- |
| *Please ask the following questions starting with:*  ***‘I would like to ask about what you like, and do not like, about the fuels you use for cooking’*** | | | | |
|  | | | | **English translation in this column please** |
| **E1.1** | First, what do you like about the fuel(s) – main and secondary?  *Check for fuels for both wet and dry seasons* | Main fuel(s) likes | |  |
| **E1.2** |  | Secondary fuel(s) likes | |  |
| **E2.1** | What do you not like about the fuel(s) – main and secondary?  *Check for fuels for both wet and dry seasons* | Main fuel(s) dislikes | |  |
| **E2.2** |  | Secondary fuel(s)dislikes | |  |
| *Please ask the following questions starting with:*  ***‘If you had the opportunity to use other types of fuel, which types would you choose and why?’***  *Fill in as many of these boxes as required* | | | | |
|  |  | | | **English translations in this column please** |
| **E3.1** | Type of fuel you would like to use | |  |  |
| **E3.2** | Why is (*insert fuel name from E3.1 here*) better? | |  |  |
| **E3.3** | What is the reason you not use it already? | |  |  |
|  | *Ask a few times to ensure the respondent has told you everything she wishes to, and write in the replies in these boxes* ***if needed****.* | | | |
| **E3.4** | Is there any other fuel you would like to use? | |  |  |
| **E3.5** | Why is (*insert fuel name from E3.5 here*) better? | |  |  |
| **E3.6** | What is the reason you not use it already? | |  |  |
| **E3.7** | Is there any other fuel you would like to use? | |  |  |
| **E3.8** | Why is (*insert fuel name from E3.8 here*) better? | |  |  |
| **E3.9** | What is the reason you not use it already? | |  |  |

| **F: FUELS USED FOR LIGHTING** | | | | | | |
| --- | --- | --- | --- | --- | --- | --- |
|  | 1 | Ethanol | 2 | Kerosene | 3 | Bottled gas (LPG) |
|  | 4 | Wood | 5 | Agri-Residues | 6 | Charcoal |
|  | 7 | Sawdust | 8 | Animal Dung | 9 | Mains electricity |
|  | 10 | Local generator | 11 | Battery | 12 | Candles |
|  | 13 | Other | 14 | None |  |  |
| **F1** | What is your main lighting fuel? *(Use list above)* | | | | |  |
| **F2** | What is your secondary lighting fuel? [If none = 14] | | | | |  |
| **F3** | About how much, in total, do you spend on lighting per week?  *If the same fuel is used for cooking and lighting, it may not be possible to split out the lighting cost. In which case enter zero* | | | | |  |

| **STOVES** | | | | |
| --- | --- | --- | --- | --- |
|  | **1** | Traditional (3-stone) fire | **2** | Improved biomass stove  Specify: |
|  | **3** | Traditional metal charcoal stove | **4** | Improved charcoal stove with ceramic liner |
|  | **5** | Ethanol stove | **6** | LPG stove |
|  | **7** | Kerosene wick stove | **8** | Kerosene pressure stove |
|  | **9** | Electric stove | **10** | Other |
|  | **11** | None |  |  |

| **G: Stove use** | | | | | |
| --- | --- | --- | --- | --- | --- |
| **G1** | How many different types of stove do you use most days? | | | |  |
| *Ask this question and fill in as many of these as required*  **‘For what purpose is each stove used?’** | | | | | |
|  | | Stove type – *use numbers* | The stove is used for…. | **English translations in this column please** | |
| **G2.1** | Stove 1 |  |  |  | |
| **G2.2** | Stove 2 |  |  |  | |
| **G2.3** | Stove 3 |  |  |  | |
| **G2.4** | Stove 4 |  |  |  | |

| **STOVES USED FOR COOKING** | | | | | | | | | |
| --- | --- | --- | --- | --- | --- | --- | --- | --- | --- |
| **MAIN COOKING STOVE** | | | | | | | | | |
| **G3.1** | What type is your main cooking stove? [use codes as listed above] | | | | |  | | | |
| **G3.2** | If non-listed stove, please describe | | **English translations here please** | | | | | | |
| **G3.3** | How many pots can be used on this stove at any one time? | | | | |  | | | |
| **G3.4** | If you paid for this stove, about how much did it cost? (Enter ‘0’ (zero) if three-stone fire or home-made) | | | | |  | | | |
| **G3.5** | If you paid for this stove, about how long (months) do you think it will be before you have to replace the stove or the liner?  *If people tell you in years, multiply by 12 and insert the answer* | | | | | | | |  |
| **G3.6** | Has this stove needed repair? | | | 1 = Yes  2 = No (go to G3.9) | | | | |  |
| **G3.7** | How many times have you had to have it repaired? (put ‘0’ if not repaired) | | | | | | | |  |
| **G3.8** | About how much (total) did repair cost?  *[Enter ‘0’ (zero) if no cost and add up the costs if more than one repair]* | | | | | | | |  |
| **G3.9** | Condition of stove  *Discuss with interviewee and examine the stove, and decide what on how you would classify stove* | **1** = good condition  **2** = Fair condition (Stove works properly but there are: Bent, loose, or broken parts; Some corrosion, Damaged grate, Pot or stove wobbles)  **3** = Poor condition (Stove is broken, does not work properly, bad corrosion, leakage of fuel, damaged grate, multiple cracks in lining, pieces missing) | | | | | | |  |
| **G4.1** | What times of day do you usually have this main stove alight?  *[Use 24 hour clock: hh:mm]* | **First** time lit | | | | |  | | |
| **G4.2** |  | Time goes out/turned off after first period used | | | | |  | | |
| **G4.3** |  | **Second** time lit | | | | |  | | |
| **G4.4** |  | Time goes out/turned off after second period used | | | | |  | | |
| **G4.5** |  | **Third** time lit | | | | |  | | |
| **G4.6** |  | Time goes out/turned off after third period used | | | | |  | | |
| **SECONDARY COOKING STOVE**  *Only ask these questions if the person has more than one stove – otherwise go to G6.1* | | | | | | | | | |
| **G5.1** | If you have a secondary cooking stove, what type is it? [Enter code = 11 if no secondary stove, and move to question G6.1] | | | | | | | **NUM** | |
| **G5.2** | If non-listed secondary stove, please describe | | | | **English translations here please** | | | | |
| **G5.3** | How many pots can be used on this stove at any one time? | | | | | | |  | |
| **G5.4** | If you paid for this stove, about how much did it cost? (Enter ‘0’ (zero) if three-stone fire or home-made) | | | | | | |  | |
| **G5.5** | If you paid for this stove, about how long (months) do you think it will be before you have to replace the stove or the liner?  *If people tell you in years, multiply by 12 and insert the answer* | | | | | | |  | |
| **G5.6** | Has this stove needed repair? | | | 1 = Yes  2 = No (go to G5.9) | | | |  | |
| **G5.7** | How many times have you had to having it repaired? | | | | | | |  | |
| **G5.8** | About how much (total) did repair cost?  *[Enter ‘0’ (zero) if no cost and add up the costs if more than one repair]* | | | | | | |  | |
| **G5.9** | Condition of stove | 1 = good condition  2 = Fair condition (Stove works properly but there are: Bent, loose, or broken parts; Some corrosion, Damaged grate, Pot or stove wobbles)  3 = Poor condition (Stove is broken, does not work properly, bad corrosion, leakage of fuel, damaged grate, multiple cracks in lining, pieces missing) | | | | | |  | |

| **LOCATION OF CHILDREN WHEN COOKING IS TAKING PLACE** | | | | | | |
| --- | --- | --- | --- | --- | --- | --- |
| **G6.1** | When you are cooking, where usually is your **youngest child**?  *[this is the child about whom information is being collected in this study]* | | 1 = With you in the kitchen carried on your back  2 = With you in the kitchen but not on your back  3 = In another room of the house  4 = Elsewhere | | |  |
|  | | | | | **English translation here please** | |
| **G6.2** | If the child is elsewhere, please describe and with whom: | Where does the child stay? | |  |  | |
| **G6.3** |  | With whom does the child stay? | |  |  | |

| **H: USE OF STOVE FOR SPACE HEATING (if required)** | | | | | | |
| --- | --- | --- | --- | --- | --- | --- |
| **1 = Traditional (3-stone) fire** | | | **2 = Improved biomass stove**  **Specify:** | | | |
| **3 = Traditional metal charcoal stove** | | | **4 = Improved charcoal stove with ceramic liner** | | | |
| **5 = Ethanol stove** | | | **6 = LPG stove** | | | |
| **7 = Kerosene wick stove** | | | **8 = Kerosene pressure stove** | | | |
| **9 = Electric stove** | | | **10 = Other** | | | |
| **11 = None** | | | **12 = Room heater (not used for cooking)** | | | |
| **H1** | Do you ever use your stove for warmth in your house? | | | | 1 = Yes  2 = No (go to Question J1) |  |
| **H2.1** | If Yes, which stove type do you use for warmth? [Use codes from table above] | | | | |  |
| **H2.2** | If it is a non-listed stove, please describe | | | **English translation in here please** | | |
| **H3** | If Yes, how do you use the stove for heating | 1 = only while cooking  2 = during day time additional to cooking times  3 = during night time additional to cooking times  4 = during day time and night time additional to cooking times | | | |  |
| **H4.1** | For about how many months of the year do you use the stove for warming the house in this way? | | | | During wet season (months) |  |
| **H4.2** |  |  |  |  | During dry season (months) |  |

| **J: USE OF FUEL FOR ENTERPRISE AND SMALL BUSINESS** | | | | | | | | | | | | |
| --- | --- | --- | --- | --- | --- | --- | --- | --- | --- | --- | --- | --- |
|  | | 1 | Ethanol | 2 | Kerosene | | | | 3 | Bottled gas (LPG) | |  |
|  |  | 4 | Wood | 5 | Agri-Residues | | | | 6 | Charcoal | |  |
|  |  | 7 | Sawdust | 8 | Animal Dung | | | | 9 | Mains electricity | |  |
|  |  | 10 | Local generator | 11 | Battery | | | | 12 | Candles | |  |
|  |  | 13 | Other | 14 | None | | | |  |  | |  |
| **J1** | Do you use fuel in your own house for an enterprise or small business? | | | | | | | 1 = Yes  2 = No (go to Question K1) | | |  |  |
| **J2.1** | If Yes, which fuel do you use for enterprise/small business? [Use codes from table above] | | | | | | |  | | | |  |
| **J2.2** | If non-listed fuel, please describe | | | | | **English translation in here please** | | | | | |  |
| **J3** | If Yes, for how many days each week do you usually use fuel in this way? | | | | | | | Days per week | | |  |  |
| **J4** | About what fraction of all the fuel you use at home is for enterprise or small business? | | | | | | | 1 = None or very little  2 = Quarter  3 = Half  4 = Three-quarters  5 = Almost all | | |  |  |
| **J5** | What is your enterprise or business? | | | | | | | 1 = Food for sale  2 = Drink for sale  3 = Other (describe below) | | |  |  |
| **J6** | If it is a non-listed enterprise, please describe | | | | | | **English translation in here please** | | | | |  |

| **K: USUAL FOOD COOKED and NUMBER OF PEOPLE FOR WHOM FOOD IS COOKED** | | | | | | | |
| --- | --- | --- | --- | --- | --- | --- | --- |
| **1 = Ugali** | | **2 = Fish or shellfish** | | | | **3 = Meat** | |
| **4 = Other meat** | | **5 = Vegetables** | | | | **6 = Beans** | |
| **7 = Greens** | | **8 = Potatoes (Yams, Irish)** | | | | **9 = Maize** | |
| **10 = Chipatis** | | **11 = Eggs** | | | | **12 = Porridge** | |
| **13 = Rice** | | **14 = Beverages (tea, coffee etc.)** | | | | **15 = Other** | |
| **K1.1** | **What foods /drinks do you usually cook each day in the wet season?**  **[Use code number from table above]** | | | | **Food 1** | |  |
| **K1.2** |  |  |  |  | **Food 2** | |  |
| **K1.3** |  |  |  |  | **Food 3** | |  |
| **K1.4** |  |  |  |  | **Food 4** | |  |
| **K2.1** | **What foods / drinks do you usually cook each day in the dry season?**  **[Use code number from table above]** | | | | **Food 1** | |  |
| **K2.2** |  |  |  |  | **Food 2** | |  |
| **K2.3** |  |  |  |  | **Food 3** | |  |
| **K2.4** |  |  |  |  | **Food 4** | |  |
| **K2.5** | **If it is a non-listed food, please describe** | | | **English translation in here please** | | | |
| **K3.1** | **How many people do you usually cook for in the household – including yourself?**  ***[Do not include cooking done for other people as a business activity]*** | | **Male children less than 15 years** | | | |  |
| **K3.2** |  |  | **Female children less than 15 years** | | | |  |
| **K3.3** |  |  | **Male adults 15 years and over** | | | |  |
| **K3.4** |  |  | **Female adults 15 years and over** | | | |  |

**Thank you for answering those questions about your household. Now, I would like to talk to you a bit more about how you cook and what do you think about your current stove. We really appreciate your time. Your thoughts and ideas about cooking with your current stove are very important to us. The people who design the new stoves may be able to improve these stoves with your suggestions. There are no right or wrong answers to any of these questions, so please feel comfortable to speak freely.**

| **L. QUALITATIVE INTERVIEW** |
| --- |
| 1. **I would like for you to tell me about how you normally cook. Please tell me what you think about the stove that you currently use for cooking.** *Probe: if they don’t talk about these issues, probe with the questions below.* |
| - How long have you been cooking on this stove? - How long does it normally take you to cook a dish like ugali on your stove? - How easy or hard is it for you to use your current stove? Why? - How much time does it take you to light your stove? How easy or hard is it for you to light your stove? Why? - How easy or hard is it for you to keep the fire going in your stove? Why? - Are you able to adjust the flame in your stove for cooking? How easy or hard is it for you to adjust the flame for cooking? Tell me more. - Are you able to add fuel to the stove while cooking? How easy or hard is it to deal with the fuel while cooking? Tell me more. - Does anyone else in your house cook on your stove? - Have you ever taught anyone else how to cook on your stove? Could you tell me about how you taught them to use your stove? - Do you know anyone else that uses a different type of stove to cook than the stove that you have? What do you know about their stove? What do you think of their stove? - Have you ever tried to use another type of stove than the one that you have? - Is there another stove that you use for cooking? *If so, repeat question and probes for alternate stove.* |
| 1. **I would like to talk about the style, look, and shape of the cookstove. Please tell me what you think about when you look at your cookstove** *(This is about physical aspects of the stove)*   *Probe: If they don’t talk about these issues, probe with the questions below.* |
| - Did you like or dislike the shape/style of your current stove? Why? - How does this shape/style of cookstove fit in your house? - Please tell me about any concerns you have about the style, material or the different parts of this cookstove?   -How about the height, size, shape? How about the space where you have to put the fuel?   - What would you change about this stove? - What do your family and friends think about your cookstove? |
| 1. **I want you to think about some of the health issues you may have experienced with your stove. Did you experience any of the following symptoms? (***Ask the checklist***) Did you experience any of the following symptoms with the new stove? (***Ask current stove first and check all the answers. Once you have completed the checklist ask the following question.)* |
| \| **Current Cookstove** \| **yes** \| **no** \| \| --- \| --- \| --- \| \| Eyes water, burn, cry tears \|  \|  \| \| Cough, phlegm \|  \|  \| \| Runny nose \|  \|  \| \| Chest tightness, pain \|  \|  \| \| Breathing problems \|  \|  \| \| Likely to get burned \|  \|  \| |
| 1. **What do you think about the smoke from your current stove?**   *Probe: (if they don’t talk about these issues, probe with the questions below.)* |
| - Tell me about the smoke from your stove. Do you notice any changes in your health when you are cooking? - How do you feel that the amount of fuel this stove uses? - What are your concerns about smoke in your household? |
| 1. **Money is hard to come by for many people. How much would you be willing to pay for this cookstove?**   *Probe: (if they don’t talk about these issues, probe with the questions below.)* |
| - How would you and your husband make the decision to spend money on a new cookstove? - What would be important for you to know when convincing your husband to spend money on a new cookstove? (Money saved on fuel? Time saved? Better or easier cooking for you? etc.) - Where would the money come from to purchase a new cookstove? - What would you have to give up in order to purchase a new cookstove? |
| 1. **Overall, how acceptable is the community to cooking on this type of stove? Is this a stove that your community would spend money on and cook with?** *Probe: if they don’t talk about these issues, probe with the questions below.* |
| - What would be the barriers to your community purchasing and cooking on this stove? - What would be the motivators to get people in your community to purchase and cook on this stove? - How would you promote this stove to your family and friends? |
| 1. **Is there anything else you would like to tell us about your experience with this cookstove?** |
|  |

PLEASE ADMINISTER CONJOINT QUESTIONNAIRE NOW.

| **M: Completion of Day 1 interview** | | | | | |
| --- | --- | --- | --- | --- | --- |
| **M1** | Interview end time (use 24-hr clock) | | |  | |
| **SET UP PERSONAL MONITOR FOR WOMAN AND INFANT AND ENSURE THAT IT IS COMFORTABLE TO WEAR AND THE WOMAN IS CLEAR ABOUT IT** | | | | | |
| **M2** | Start time for setting up personal monitors | | | |  |
| **M3** | Confirm arrangement to return the following day at approximately the same time | 1 = Yes  2 = Woman does not wish to continue in the study  3 = Happy to take part, but unavailable at time requested (describe plan for return in K3) | | |  |
| **M4** | If woman is not available for the return visit at the time requested, plan other arrangements  *Discuss with supervisor if needed.* | When is she available and what has been arranged? | **English translation in here please** | | |
| **Thank you very much for your time and assistance.** | | | | | |

**NOTES TO INTERVIEWER:**

**Before leaving, re-confirm to the woman that she and the child should never part with the badge and the tube. Ask if she understood properly what has to be done with these at night, when washing (herself and child) and when going out of the house.**

**Encourage her to go on with her usual occupation, and not to do anything different from usual.**

## General background, biomarker, and stove-related air pollution exposure assessment [Dholuo version]

Baseline Questionnaire

(To be administered prior to the 48-hr air sampling)

| **A: IDENTIFICATION INFORMATION/YANGRUOK** | | |
| --- | --- | --- |
| **A1.1** | **Study household ID number/Namba ot mar nonro** |  |
| **A2.1** | **Neighbourhood/Gweng** |  |
| **A3.1** | **Name [ID] of Interviewer/Nying/namba jachiw penjo (NOT INTERVIEWEE/OK NGAMA IPENJO)** |  |
| **A4.1** | **Date of interview/Tarik mar penjo (DD/MM/YYYY)** | **__ __/ __ __ / __ __ __ DATE/TARIK__** |
| **A5.1** | **Start time of interview/Saa ma ichako penjo (Use 24 hour clock/Tii gi saa 24)** | **_TIME/SAA_ __ / __ __** |

| **SETTING UP EQUIPMENT FOR PERSONAL MONITORING/KETO RAMENY MAR LUWO NGATO**  **Explain that we will set up kitchen monitor and the monitor for the woman, and the tube on the child, after asking the questions/ler ni wabiro keto rameny iro e jikon togi kuom miyo, kendo mpira kuom nyathi bang penjo.** |
| --- |

| **B: FAMILY INFORMATION/WECHE JO OT** | | | |
| --- | --- | --- | --- |
| **B1** | **How old are you/in jahigni adi (years/Higni)?** | | **NUM** |
| **B2** | **Marital status/Chal mar kend: Are you/Bende …..** | **1 = Married/okendi**  **2 = Single mother/Miyo maok okendi**  **3 = Separated/Iweyo dichuo/dhako**  **4 = Divorced/Iwecho e nyim serikal**  **5 = Widowed/Chwori/chiegi otho** | **NUM** |
| **B3** | **How many people (in the age groups below) usually live in this house/Ji adi kuom higa majadak e ot ka?** | |  |
| **B3.1** | **Males/Jowuoyi** | **Less than 5 years/Bwo higni abich:** |  |
| **B3.2** |  | **5-14 years/Higni 5-14** |  |
| **B3.3** |  | **15 years and older/Mokalo higni 15** |  |
| **B3.4** | **Females/Nyiri (including yourself/Ka oriwore kodi)** | **Less than 5 years//Bwo higni abich:** |  |
| **B3.5** |  | **5-14 years/Higni 5-14** |  |
| **B3.6** |  | **15 years and older/Mokalo higni 15** |  |
| **B4** | **How many people in total usually live in this house/Ji adi duto majadak e ot ka? [Check/Ngi]** | |  |
| **B5.1** | **What is the age of your youngest child/Nyathini matin jahigni adi?** | **Years/Higni** |  |
| **B5.2** |  | **Months/Dweche** |  |
| **B6** | **What is the sex of the youngest child?/Nyathi matin mogik en wuoyi koso nyako?** | **1 = Male/Wuoyi**  **2 =Female/Nyako** |  |

| **C: SOCIO-ECONOMIC CIRCUMSTANCES OF THE HOUSEHOLD** | | | | | | | | | | |
| --- | --- | --- | --- | --- | --- | --- | --- | --- | --- | --- |
| **EDUCATION/CHAL GI SOMO MAR JOOT** | | | | | | | | | | |
| **C1.1** | **If you attended school, for how many years did you attend/Kane idhi school, ne idhi kuom higni adi?**  ***(if did not attend school, enter ‘0’/Kane ok idhi school, ndik ‘0’ (zero/Nono))*** | | | | | | | | |  |
| **C1.2** | **What level of education did you reach/Ichopo e okang mane mar somo?** | | | | | **1 = No formal education/Ok adhi skul**  **2 = Primary/Primari**  **3 = Secondary/Sekondari**  **4 = Higher/Haya** | | | |  |
| **C2.1** | **Are you comfortable with reading/Bende in thuolo gi somo?** | | | | | **1 = Yes/Ee 2 = No/Ooyo** | | | |  |
| **C2.2** | **Are you comfortable with writing/Bende in thuolo gi ndiko?** | | | | | **1 = Yes/Ee 2 = No/Ooyo** | | | |  |
| **C3.1** | **If your husband attended school, for how many years did he attend/Ka jaodi ne odhi skul, ne odhi kuom higni adi?**  ***(if he did not or not applicable, put '0'/Kane ok odhi kata ok oluore, to ket ‘0’(zero/Nono) and go to C6.1/ae to idhi e penjo mar C6.1 )*** | | | | |  | | | |  |
| **C3.2** | **What level of education did your husband reach/Jaodi ochopo e okang mane mar somo?** | | | | | **1 = No formal education/Ok adhi skul**  **2 = Primary/Primari**  **3 = Secondary/Sekondari**  **4 = Higher/Haya** | | | |  |
| **C4.1** | **Is your husband comfortable with reading/Bende jaodi ni thuolo gi somo?** | | | | | **1 = Yes/Ee 2 = No/Ooyo** | | | |  |
| **C4.2** | **Is your husband comfortable with writing/Bende jaodi ni thuolo gi ndiko?** | | | | | **1 = Yes/Ee 2 = No/Ooyo** | | | |  |
| **EMPLOYMENT/TICH** | | | | | | | | | | |
|  | **1** | **Farms his/her own land (whether that land is owned or rented)/Puro puothe owuon (Kaen puothe kata ma ochulo kuom kinde)** | **6** | | **Craftsperson (tailor, carpenter, seamstress etc.)/Fundi(Fundi charan, fundi bao,jatwang law)** | | | | | |
|  | **2** | **Day labourer (farming another person’s land, house-building etc. ) /Jatij lwedo (Puro puoth ngama chielo, jagedo)** | **7** | | **Runs the household / Cares for family/Ngiyo kata rito joode** | | | | | |
|  | **3** | **Government employee (doctor, nurse, police, teacher etc. )/Jatij serikal(Laktar, nas, polis, japuonj)** | **8** | | **Retired/Oseritaya** | | | | | |
|  | **4** | **Employee in a business (Factory worker, works in a shop)/Jatich e (kambi, duka)** | **9** | | **Other type of job/Tich machielo** | | | | | |
|  | **5** | **Has own business (owns a shop etc.)/Nigi ohande owuon(Nigi duka)** | **10** | | **Currently unemployed/Onge tich gi esani** | | | | | |
| **C5.1** | **What is your husband’s main occupation / job? *Use list above*/Tij jaodi maduong en ango? Tii gi listi man malo** | | | | | | **First/Mokuongo** | |  | |
| **C5.2** | **If your husband has a second job, what is it?/ka jaodi nigi tich machielo, en mane?** | | | | | | **Second/Mar ariyo** | |  | |
| **C5.3** | **If his occupation is not listed, please describe here/ka tich maotiyo ok ondik malo, yie iler** | | | **English translation here please** | | | | | | |
| **C6.1** | **What is your main occupation or job? *Use list above***  **Tiji maduong en ango? Tii gi listi man malo** | | | | | | **First/Mokuongo** |  | | |
| **C6.2** | **If you have another occupation, what is it? /ka in gi tich machielo, en mane?** | | | | | | **Second /Mar ariyo** |  | | |
| **C6.3** | **If your secondary occupation is not listed, please describe here/ka tich maitiyo ok ondik malo, yie iler** | | | **English translation here please** | | | | | | |

| **WATER AND SANITATION/PI GI LER** | | | | | | | | | | | | | |
| --- | --- | --- | --- | --- | --- | --- | --- | --- | --- | --- | --- | --- | --- |
| **C7.1** | **Do you have access to drinking water in your house?/Bende uyudo pi modho e odi?** | | | **1 = Yes/Ee**  **2 = No/Ooyo** | | | | | | |  | | |
| **C7.2** | **Where do you obtain most of your water for your household needs?/Ugolo thoth pi ma utiyogo e odi kanye?** | | | **1 = Piped in home/Freji ni e dala**  **2 = Pump (deep well)/Goyo pi kisima**  **3 = Well (pit with bucket)/Tuomo pi kisima gi ndoo**  **4 = Communal standpipe//Freji mar ji duto**  **5 = Collect from river/Omo e aora** | | | | | | |  | | |
| **C8** | **Do you have a septic tank toilet inside the house or a latrine in the yard?/Bende in gi choo e ot koso in gi choo bur maoko** | | | **1= Inside your house/E ot**  **2 = In the yard/E laro**  **3= None/Onge** | | | | | | |  | | |
| **POSSESSIONS & INCOME/NWENGO&YUTO** | | | | | | | | | | | | | |
| **C9** | **Do you own/have any of the following?/Bende in gi achiel kuom ma oluoregi** | |  | | | | | | | | | | |
| **C9.1** |  |  | **Motor-bike/Apiko** | | | | | | **1 = Yes/Ee 2= No/Ooyo** | | |  | |
| **C9.2** |  |  | **Refrigerator/Aguch pee** | | | | | | **1 = Yes/Ee 2= No/Ooyo** | | |  | |
| **C9.3** |  |  | **Electricity connection/Sitima** | | | | | | **1 = Yes/Ee 2= No/Ooyo** | | |  | |
| **C9.4** |  |  | **Access to electricity generator/Tiyo gi generator** | | | | | | **1 = Yes/Ee 2= No/Ooyo** | | |  | |
| **C9.5** |  |  | **Radio** | | | | | | **1 = Yes/Ee 2= No/Ooyo** | | |  | |
| **C9.6** |  |  | **Hi-Fi / CD-player** | | | | | | **1 = Yes/Ee 2= No/Ooyo** | | |  | |
| **C9.7** |  |  | **TV** | | | | | | **1 = Yes/Ee 2= No/Ooyo** | | |  | |
| **C9.8** |  |  | **Bicycle/Ndiga** | | | | | | **1 = Yes/Ee 2= No/Ooyo** | | |  | |
| **C9.9** |  |  | **Car/truck/Mtoka/ lori** | | | | | | **1 = Yes/Ee 2= No/Ooyo** | | |  | |
| **C9.10** |  |  | **Cell phone/Sime mar lwedo** | | | | | | **1 = Yes/Ee 2= No/Ooyo** | | |  | |
| **C9.11** |  |  | **Shower / bath within house/Od luok e ot** | | | | | | **1 = Yes/Ee 2= No/Ooyo** | | |  | |
| **C9.12** |  |  | **Cow/Dhiang** | | | | | | **1 = Yes/Ee 2= No/Ooyo** | | |  | |
| **C10** | **Would you mind us asking a question about your family’s weekly income/Bende inyalo yienwa penjo kuom yuto joodi e juma?** | | | | | | | | **1 = Yes/Ee (go to D1/Dhi e penjo mar D1)**  **2 = No/Ooyo** | | |  | |
| **C11** | **About how much money do you have available for household purchases each week?/Pesa marom nade maingo mar nyiewo gi ot juma ka juma?** | | | | | | | |  | | | | |
| **C12** | **Do you feel that this amount of money is …/Bende ineno ni pesani oromo** | | | | | **1 = Enough to buy everything needed/Oromo nyiewo gimoro amora ma dwarore**  **2 = Not quite enough/Ok oromo ruok**  **3 = Much too little/Tin ahinya** | | | | | |  | |
| **TOBACCO SMOKING** | | | | | | | | | | | | | |
| **C13.1** | | **Do you smoke cigarettes, or have you ever smoked cigarettes for one year or more?/bende imadho ndawa, kata bende isemadho ndawa kuom higa achiel kata mokalo?** | | | | | **1 = Yes/Ee**  **2 = No/Ooyo (Go to C14/Di e penjo mar C14)** | | | | | |  |
| **C13.2** | | **Have you/bende ise:**  **1 = continued to smoke (that is, a current smoker)?/dhi nyime gi madho ndawa(Mano, jamadh ndawa)**  **2 = given up smoking altogether, within the last 4 weeks?/weyo madho ndawa, e jumbe ariyo mokalo?**  **3 = have stopped completely for more than 4 weeks?/weyo madho ndawa chuth kuom ndalo mokalo jumbe 4?** | | | | | | | | | | |  |
| **C13.3** | | **How many cigarettes do (did) you smoke each day, on average?/ne imadho ndawa adi odiochieng ka odiochieng?** | | | | | | | | | | |  |
| **C14** | | **Does anyone else in your household smoke cigarettes?/Bende ngama chielo e odu madho ndawa?** | | | | | **1 = Yes/Ee**  **2 = No/Ooyo (Go to Question L12.1/Dhi e penjo mar L12.1)** | | | | | |  |
| **C14.2** | | **If Yes, do they smoke in the house, or only outside the house?/Ka ee, gimadho e ot, koso mana oko kende?** | | | **1 = Inside (at least sometimes)/E ot (Seche moko)**  **2 = Outside only (Go to Question L12.1)/Oko(/Dhi e penjo mar L12.1)** | | | | | | | |  |
| **C14.3** | | **How many people smoke cigarettes inside the house?/Ji adi ma madho ndawa e ot** | | | | | | | | **Number of people/kwan ji** | | |  |
| **C14.4** | | **About how many cigarettes in total are smoked each day in your house by other people?/Gin kar ndawa adi duto ma imadho pile pile e odi gi joma moko?** | | | | | | **Number of cigarettes per day/kwan ndawa odiochieng ka odiochieng** | | | | |  |

| **D: FUELS USED FOR COOKING/GIK MA ITIYOGO E KELO MACH** | | | | | | | | | | | | |
| --- | --- | --- | --- | --- | --- | --- | --- | --- | --- | --- | --- | --- |
| **FUELS LIST/LISTI MAR MA ITIYOGO E KELO MACH** | | | | | | | | | | | | |
|  | | **1** | **Ethanol** | **2** | **Kerosene/mafuta** | | | | **3** | | **Bottled gas (LPG)/Gas** | |
|  |  | **4** | **Wood/Yien** | **5** | **Agri-Residues/Yuk puodho** | | | | **6** | | **Charcoal/Makaa** | |
|  |  | **7** | **Sawdust/Randa** | **8** | **Animal Dung/owuoyo** | | | | **9** | | **Electricity/Sitima** | |
|  |  | **10** | **Local generator** | **11** | **Battery** | | | | **12** | | **Candles/Misuma** | |
|  |  | **13** | **Other/machielo** |  | | | | | | | | |
| **D1** | **What is your main cooking fuel … *(please use the list above)/Mach mane ma ijatedogo ahinya …(Tii gi listi man malo)***  **…..in the wet season/Kinde ngich**  **…..in the dry season/Kinde ma piny otwo** | | | | | | | | |  | | |
| **D1.1** |  |  |  |  |  |  |  |  |  | **Wet/Ngich** | |  |
| **D1.2** |  |  |  |  |  |  |  |  |  | **Dry/Otwo** | |  |
| **D1.3** | **If any non-listed main fuel (wet season), describe here/ka kit mach ma ojatedgo ahinya ok ondiki (Kinde ngich), ler ka:** | | | | | | |  | | | | |
| **D1.4** | **If any non-listed main fuel (dry season), state here/ka kit mach ma ojatedgo ahinya ok ondiki (Kinde ma piny otwo), ler ka:** | | | | | | |  | | | | |
| **D2** | **What is your secondary cooking fuel, if used…/En mach mane machielo ma itiyogo, kaponi itiyogo**  **…..in the wet season/Kinde ngich**  **…..in the dry season/Kinde ma piny otwo** | | | | | | | | |  | | |
| **D2.1** |  |  |  |  |  |  |  |  |  | **Wet/Ngich** | |  |
| **D2.2** |  |  |  |  |  |  |  |  |  | **Dry/Otwo** | |  |
| **D2.3** | **If any non-listed secondary fuel (wet season), state here ka kit mach ma ojatedgo ahinya ok ondiki (Kinde ngich), ler ka:** | | | | | **English translations here please** | | | | | | |
| **D2.4** | **If any non-listed secondary fuel (dry season), state here ka kit mach ma ojatedgo ahinya ok ondiki (Kinde ma piny otwo), ler ka:** | | | | |  | | | | | | |
| **D2.5** | **What proportion of the total fuel is the secondary fuel?/ Mach machielo ma itedogo duto rom nade?** | | | | | | **1= about half/Madirom nus**  **2 = about quarter/Madirom robo**  **3 = Very small amount/Matin ahinya** | | | | |  |
|  | ***IN THIS NEXT SECTION, CHECK WHICH FUELS THE INTERVIEWEE USES, AND ASK QUESTIONS ONLY ABOUT THOSE FUELS/E KIDIENYNI, NGI KIT GI TEDO MA NGAMA IMIYO PENJO TIYOGO KENDO IPENJ PENJO MANA KUOM GIK MAKELO MACHGI*** | | | | | | | | | | | |

|  | **CHARCOAL USERS ONLY/JOMA TIYO GI MAKA KENDE** | | | | |
| --- | --- | --- | --- | --- | --- |
| **D3.1** | **CHARCOAL USERS/JOMA TIYO GI MAKA**  **Complete this if interviewee answered ‘6’ to any of these questions D1.1 D1.2**  **D2.1 D2.2/Ndik ma ka jaduok penjo ne oduoko ni ‘6’ ewi achiel kuom penjo ma pinygi D1.1 D1.2**  **D2.1 D2.2**  ***You may need to weigh one or two bags of charcoal if the woman does not know their weight/Inyalo dwaro pimo achiel kata ariyo kuom gunde maka ka miyo ok ongeyo ratil margi*** | **Confirm: does the respondent use any charcoal for cooking?/Non: bende jaduok penjo tiyo maka kuom tedo** | | **1 = Yes/Ee**  **2 = No/Ooyo** |  |
| **D3.2** |  | **Do you buy small or large bags of charcoal?/ Inyiewo gunde maka matindo koso madongo?** | | **1 = Small/Matindo**  **2 = Large (Go to D3.5)/madongo(Dhi e penjo mar D3.5)**  **3 = Both** |  |
| **D3.3** |  | **In the wet season** | **About how many small bags of charcoal do you need to buy each week for the stove in the wet season? /Gin gunde maka matindo marom nade ma onego ingiew e juma ka juma e kinde ngich?** | |  |
| **D3.4** |  |  | **How much does one small bag weigh?/Gunia achiel matin en ratil adi?** | |  |
| **D3.5** |  |  | **How much does one small bag cost? /Gunia achiel matin en pesa adi?** | |  |
| **D3.6** |  |  | **About how many large bags of charcoal do you need to buy each week for the stove in the wet season?/Gin gunde maka madongo marom nade ma onego ingiew e juma ka juma e kinde ngich?** | |  |
| **D3.7** |  |  | **How much does one large bag weigh? /Gunia achiel maduong en ratil adi?** | |  |
| **D3.8** |  |  | **How much does one large bag cost? /Gunia achiel maduong en pesa adi?** | |  |
| **D3.9** |  |  | **About how much in total do you spend on charcoal each week in the wet season?/En pesa adi duto ma itiyogo kuom maka juma ka juma e kinde ngich?** | |  |
| **D3.10** |  | **In the dry season/Kinde ma piny otwo** | **About how many small bags of charcoal do you need to buy each week for the stove in the dry season? /Gin gunde maka matindo marom nade ma onego ingiew e juma ka juma e kinde ma piny otwo?**  ***Only ask if small bags are used/Penj mana ka gunde matindo otiigo*** | |  |
| **D3.11** |  |  | **About how many large bags of charcoal do you need to buy each week for the stove in the dry season? /Gin gunde maka madongo marom nade ma onego ingiew e juma ka juma e kinde ngich?**  ***Only ask if large bags are used/Penj mana ka gunde madongo otiigo*** | |  |
| **D3.12** |  |  | **About how much in total do you spend on charcoal each week in the dry season? /En pesa adi duto ma itiyogo kuom maka juma ka juma e kinde ma piny otwo?** | |  |

|  | **WOOD & RESIDUE USERS ONLY/JOMA TIYO GI YIEN GI YUGI KENDE** | | | | | | | | | | |
| --- | --- | --- | --- | --- | --- | --- | --- | --- | --- | --- | --- |
| **D4.1** | **WOOD & RESIDUE USERS/JOMA TIYO GI YIEN GI YUGI KENDE**  **Complete this if interviewee answered ‘4’ or ‘5’ or ‘7’ to any of these questions D1.1**  **D1.2**  **D2.1**  **D2.2/Ndik ma ka jaduok penjo ne oduoko ni ‘4’ kata ‘5’ kata ‘7’ ewi achiel kuom penjo ma pinygi D1.1 D1.2**  **D2.1 D2.2**  **WOOD & RESIDUE USERS**  **continued**  **WOOD & RESIDUE USERS**  **continued** | **Confirm: does the respondent use any wood or residues for cooking?/Non: bende jaduok penjo tiyogi yien kata yugi e tedo?** | | | | | **1 = Yes/Ee**  **2 = No/Ooyo** | | | |  |
| **D4.2** |  | **In the wet season/E kinde ngich** | **Usually, in the wet season, how much of the wood or residues you use is bought? /E kinde ngich, en yien kat ayugi marom nade ma itiyogo ma inyiewo?** | | | | **1 = All bought/Onyiew te**  **2 = Most bought/Onyiew thothne**  **3 = About half bought/Onyiew nus**  **4 = Most collected/Ochok thothne**  **5 = All collected (Go to D4.7) /Ochok te(Dhi e penjo mar D4.7)** | | | |  |
| **D4.3** |  |  | **If bought: amount bought per week in the wet season/Ka onyiew: kar ma onyiew e juma e kinde ngich** | | **Number of bundles of wood/Kwan yien ma otwe** | | | | | |  |
| **D4.4** |  |  |  |  | **Weight of bundle bought wood (kg)/Ratil mar yien ma onyiew** | | | | | |  |
| **D4.5** |  |  |  |  | **Average cost per week for all the bought wood or residues/Kwan pesa ma itiyogo kuom yien e juma ka juma** | | | | | |  |
|  |  |  | ***Ask only if any wood or residues are gathered/Penj mana ka yugi ochoki*** | | | | | | | |  |
| **D4.6** |  |  | **Number of bundles of wood or residues gathered per week in the wet season/Kwan yien ma otwe ma otigo juma ka juma e kinde ngich** | | | | | | | |  |
| **D4.7** |  |  | **Weight of one bundle of gathered wood or residue/Ratin yien achiel ma otwe kata yugi** | | | | | | | |  |
| **D4.8** |  |  | **Who collects the wood or residues?/Ngama moto kata choko yugi?** | | **Yourself/An awuon** | | | | | **1 = Yes/Ee 2= No/Ooyo** |  |
| **D4.9** |  |  |  |  | **Husband/Jaoda** | | | | | **1 = Yes/Ee 2= No/Ooyo** |  |
| **D4.10** |  |  |  |  | **Female children/Nyithindo manyiri** | | | | | **1 = Yes/Ee 2= No/Ooyo** |  |
| **D4.11** |  |  |  |  | **Male children/Nyithindo machwo** | | | | | **1 = Yes/Ee 2= No/Ooyo** |  |
| **D4.12** |  |  | **How long does each trip take, on average (hours/Nyithindo machwo** | | | | | | | |  |
| **D4.13** |  |  | **How many adults go out on each trip, on average/Joma dongo adi madhi moto** | | | | | | | |  |
| **D4.14** |  |  | **How many children go out on each trip, on average/Nyithindo adi madhi moto** | | | | | | | |  |
| **D4.15** |  | **In the dry season**  **Dry season contd** | **Usually, in the dry season, how much of the wood or residues you use is bought? /E kinde ma piny lit, en yien kat ayugi marom nade ma itiyogo ma inyiewo?** | | | | | **1 = All bought/Onyiew te**  **2 = Most bought/Onyiew thothne**  **3 = About half bought/Onyiew nus**  **4 = Most collected/Ochok thothne**  **5 = All collected *(go to D4.22*/Ochok te(Dhi e penjo mar D4.22)** | | |  |
| **D4.16** |  |  | **If bought: amount bought per week in the dry season Ka onyiew: kar ma onyiew e juma e kinde ma piny liet** | | | **Number of bundles of wood/Kwan yien ma otwe** | | | | |  |
| **D4.17** |  |  |  |  |  | **Weight of bundle bought wood (kg) /Ratil mar yien ma onyiew** | | | | |  |
| **D4.18** |  |  |  |  |  | **Average cost per week for all the bought wood or residues/Kwan pesa ma itiyogo kuom yien e juma ka juma** | | | | |  |
|  |  |  | ***Ask only if any wood or residues are gathered: /Penj mana ka yugi ochoki:*** | | | | | | | |  |
| **D4.19** |  |  | **Number of bundles wood/residues gathered per week in the dry season/Kwan yien ma otwe ma otigo juma ka juma e kinde ma piny liet** | | | | | | | | **NUM** |
| **D4.20** |  |  | **Weight bundle gathered wood or residues (kg) /Ratin yien achiel ma otwe kata yugi** | | | | | | | |  |
| **D4.21** |  |  | **Who collects the wood or residues? ?/Ngama moto kata choko yugi?** | **Yourself/An awuon** | | | | | **1 = Yes/Ee 2= No/Ooyo** | |  |
| **D4.22** |  |  |  | **Husband/Jaoda** | | | | | **1 = Yes/Ee 2= No/Ooyo** | |  |
| **D4.23** |  |  |  | **Female children/Nyithindo manyiri** | | | | | **1 = Yes/Ee 2= No/Ooyo** | |  |
| **D4.24** |  |  |  | **Male children/Nyithindo machwo** | | | | | **1 = Yes/Ee 2= No/Ooyo** | |  |
| **D4.25** |  |  | **How long does each trip take, on average (hours) /Nyithindo machwo** | | | | | | | |  |
| **D4.26** |  |  | **How many adults go out on each trip, on average/Joma dongo adi madhi moto** | | | | | | | |  |
| **D4.27** |  |  | **How many children go out on each trip, on average/Nyithindo adi madhi moto** | | | | | | | |  |

|  | **BOTTLED GAS (LPG) USERS ONLY/JOMA TIYO GI GAS KENDE** | | | | |
| --- | --- | --- | --- | --- | --- |
| **D5.1** | **LPG USERS/JOMA TIYO GI GAS**  **Complete this if interviewee answered ‘3’ to any of these questions D1.1 D1.2**  **D2.1 D2.2/Ndik ma ka jaduok penjo ne oduoko ni ‘3’ ewi achiel kuom penjo ma pinygi D1.1 D1.2**  **D2.1 D2.2** | **Confirm: does the respondent use any LPG for cooking?/Non:Bende jaduok penjo tiyo gi gas moro amora mar LPG e tedo?** | | **1 = Yes/Ee**  **2 = No/Ooyo** |  |
| **D5.2** |  | **What size bottles do you buy? (size in kg)/Gin chupni mage ma ija nyiewo? (Kuom ratil)** | | |  |
| **D5.3** |  | **How much does one bottle cost?/Chupa achiel mar gas en pesa adi?** | | |  |
| **D5.4** |  | **In the wet season/E kinde ngich** | **How long does one bottle last? (days)/Chupa achiel mar gas kaawo ndalo aadi?89Ndalo)** | |  |
| **D5.5** |  | **In the dry season/E kinde ma piny liet** | **How long does one bottle last? (days) )/Chupa achiel mar gas kaawo ndalo aadi? (Ndalo)** | |  |

|  | **KEROSENE USERS ONLY/JOMA TIYO GI MAFUTA KENDE** | | | | |
| --- | --- | --- | --- | --- | --- |
|  | ***Find out how it is bought, and work out number of mls of kerosene (eg if it is in a bottle, find out how much liquid can be contained in the bottle and find out the number of bottles used each week)Non kaka inyiewe, kendo igo kwan lita mar mafuta(ka en e chupa, non mafuta manyalo room e chupani kendo inon kar chupni ma itiyogo e juma ka juma)*** | | | | |
| **D6.1** | **KEROSENE USERS/JOMA TIYO GI MAFUTA**  **Complete this if interviewee answered ‘2’ to any of these questions D1.1, D1.2**  **D2.1, D2.2/Ndik ma ka jaduok penjo ne oduoko ni ‘2’ ewi achiel kuom penjo ma pinygi D1.1 D1.2**  **D2.1 D2.2** | **Confirm: does the respondent use any Kerosene for cooking? /Non:bende jaduok penjo tiyo gi mafuta e tedo?** | | **1 = Yes/Ee**  **2 = No/Ooyo** |  |
| **D6.2** |  | **In the wet season/E kinde ngich** | **How much kerosene do you buy each week? (mls)/Inyiewo mafuta marom nade e juma?** | | **ml** |
| **D6.3** |  |  | **About how much do you spend each week on kerosene/En mafuta madirom nade ma itiyogo e juma?** | |  |
| **D6.4** |  | **In the dry season/E kinde ma piny liet** | **How much kerosene do you buy each week? (mls)/En mafuta marom nade ma inyiwo juima ka juma?** | | **ml** |
| **D6.5** |  |  | **About how much do you spend each week on kerosene?En pesa madirom nade ma itiyogo e juma ka juma?** | |  |

|  | **ELECTRICITY USERS ONLY/JOMA TIYO GI SITIMA KENDE** | | | | |
| --- | --- | --- | --- | --- | --- |
| **D7.1** | **ELECTRICITY USERS/JOMA TIYO GI SITIMA**  **Complete this if interviewee answered ‘9’ or ‘10’ to any of these questions D1.1, D1.2**  **D2.1, D2.2/Ndik ma ka jaduok penjo ne oduoko ni ‘9’ kata ‘10’ ewi achiel kuom penjo ma pinygi D1.1 D1.2**  **D2.1 D2.2** | **Confirm: does the respondent use any electricity for cooking? /Non:bende jaduok penjo tiyo gi sitima e tedo?** | | **1 = Yes/Ee**  **2 = No/Ooyo** |  |
| **D7.2** |  | **How do you pay for your electricity/Ere kaka ichulo sitima mari?** | **1 = fixed price per day/nengo ma ondiki odiochieng ka odiochieng**  **2 = price per unit (eg kilowatt-hr)/Nengo kuom unit**  **3 = Other/machiel** | |  |
| **D7.3** |  | **What is the price of electricity per unit? *Insert units for both price and type of unit (eg Ar / kw-hour)/Nengo sitima kuom unit en adi? (Ndik unit ne nengo togi kit unit duto)*** | | |  |
| **D7.4** |  | **In the wet season/E kinde ngich** | **How much do you pay for your electricity per week/En pesa adi ma ichulo kuom sitima juma ka juma?** | |  |
| **D7.5** |  | **In the dry season/E kinde ma piny liet** | **How much do you pay for your electricity per week/ En pesa adi ma ichulo kuom sitima juma ka juma?** | |  |

| **E: FUEL PREFERENCES/KIT GIK MA KELO MACH MAITEDOGO MA OYIER** | | | | |
| --- | --- | --- | --- | --- |
| ***Please ask the following questions starting with:/Yie ipenj ma oluoregi ka ichako gi:***  ***‘I would like to ask about what you like, and do not like, about the fuels you use for cooking’/Daher penjikuom gima ihero gi maok ihero kuom gi tedo ma itiyogo*** | | | | |
|  | | | | **English translation in this column please** |
| **E1.1** | **First, what do you like about the fuel(s) – main and secondary? /Mokuongo, ango ma ihero kuom mach ma ijatiyogo ahinya togi mamoko ma itiyogo?**  ***Check for fuels for both wet and dry seasons/Ngi gi tedo duto e kinde ngich gi kinde ma piny otwo*** | **Main fuel(s) likes/Kit gik makelo mach madongo ma oher** | |  |
| **E1.2** |  | **Secondary fuel(s) likes/Kit gik makelo mach mamoko ma oher** | |  |
| **E2.1** | **What do you not like about the fuel(s) – main and secondary? /Mokuongo, ango ma ihero kuom mach ma ijatiyogo ahinya togi mamoko ma itiyogo?**  ***Check for fuels for both wet and dry seasons/Ngi kit mach duto e kinde ngich gi kinde ma piny otwo*** | **Main fuel(s) dislikes/Kit gik makelo mach madongo ma oher** | |  |
| **E2.2** |  | **Secondary fuel(s)dislikes/Kit gik makelo mach mamoko ma oher** | |  |
| ***Please ask the following questions starting with: /Yie ipenj ma oluoregi ka ichako gi:***  ***‘If you had the opportunity to use other types of fuel, which types would you choose and why?’/’Kane ne in gi thuolo mar tiyo kit mach mamoko, gin mage ma inyalo yiero to nango?’***  ***Fill in as many of these boxes as required/Ndik mageny kaka nyalore*** | | | | |
|  |  | | | **English translations in this column please** |
| **E3.1** | **Type of fuel you would like to use/Kit gik makelo mach madiher tiyogo** | |  |  |
| **E3.2** | **Why is (*insert fuel name from E3.1 here*) better?/Ango ma omiyo(Ndik nying gir tedo ka) ber moloyo?** | |  |  |
| **E3.3** | **What is the reason you not use it already?/Ango ma omiyo pod ok iti kode?** | |  |  |
|  | ***Ask a few times to ensure the respondent has told you everything she wishes to, and write in the replies in these boxes if needed./Penj kendo mondo mi ine ni jaduok penjo onyisi duto madoher,kendo indik duoko e buchego ka nyalore*** | | | |
| **E3.4** | **Is there any other fuel you would like to use?/Bende nitie gir kit kendo machielo madiher tiyogo?** | |  |  |
| **E3.5** | **Why is (*insert fuel name from E3.5 here*) better? /Ango ma omiyo(Ndik nying gir tedo ka owuok e penjo mar E3.5 ka) ber moloyo?** | |  |  |
| **E3.6** | **What is the reason you not use it already? /Ango ma omiyo pod ok iti kode?** | |  |  |
| **E3.7** | **Is there any other fuel you would like to use?/ Bende nitie gir kit kendo machielo madiher tiyogo?** | |  |  |
| **E3.8** | **Why is (*insert fuel name from E3.8 here*) better? /Ango ma omiyo(Ndik nying gir tedo ka owuok e penjo mar E3.8 ka) ber moloyo?** | |  |  |
| **E3.9** | **What is the reason you not use it already? /Ango ma omiyo pod ok iti kode?** | |  |  |

| **F: FUELS USED FOR LIGHTING/GIK MA ITIYOGO E MENYO OT** | | | | | | |
| --- | --- | --- | --- | --- | --- | --- |
|  | **1** | **Ethanol** | **2** | **Kerosene/Mafuta** | **3** | **Bottled gas (LPG)/Gas** |
|  | **4** | **Wood/Yien** | **5** | **Agri-Residues/Yugi mar puodho** | **6** | **Charcoal/Makaa** |
|  | **7** | **Sawdust/Randa** | **8** | **Animal Dung/Owuoyo** | **9** | **Mains electricity/Sitima** |
|  | **10** | **Local generator** | **11** | **Battery** | **12** | **Candles/Misuma** |
|  | **13** | **Other/machielo** | **14** | **None /Onge** |  |  |
| **F1** | **What is your main lighting fuel? *(Use list above)/Gir menyo ot ma ijatiyogo ahinya en mane?(Tiigi listi man malo)*** | | | | |  |
| **F2** | **What is your secondary lighting fuel? [If none = 14]/ *Gir menyo ot mari machielo en mane?[ka onge=14]*** | | | | |  |
| **F3** | **About how much, in total, do you spend on lighting per week?/En kar pesa adi ma itiyogo e menyo ot juma ka juma?**  ***If the same fuel is used for cooking and lighting, it may not be possible to split out the lighting cost. In which case enter zero?ka gima itiyogo emabende itedogo, ok nyal bedo mayot e pogo kar pesa. Ka en kamano to ndik nono*** | | | | |  |

| **STOVES** | | | | |
| --- | --- | --- | --- | --- |
|  | **1** | **Traditional (3-stone) fire/Mach mar kendo (kendo mar kite 3)** | **2** | **Improved biomass stove**  **Specify:/Kendo matiyogi owuoyo** |
|  | **3** | **Traditional metal charcoal stove/Jiko** | **4** | **Improved charcoal stove with ceramic liner/Jiko ma ochue iye** |
|  | **5** | **Ethanol stove/ Kendo matiyogi ethanol** | **6** | **LPG stove/ Kendo mar gas** |
|  | **7** | **Kerosene wick stove/ Kendo mar kitambi** | **8** | **Kerosene pressure stove/ Kendo ma ikudho** |
|  | **9** | **Electric stove/ Kendo mar sitima** | **10** | **Other/Machielo** |
|  | **11** | **None/Onge** |  |  |

| **G: Stove use/Tiyo gi stove** | | | | | |
| --- | --- | --- | --- | --- | --- |
| **G1** | **How many different types of stove do you use most days?/Gin kit stove adi ma ija tiyogo?** | | | |  |
| ***Ask this question and fill in as many of these as required/Penj penjoni kendo indik mangeny kaka nyalore***  **‘For what purpose is each stove used?’/’ Kendo ka stove itiyogo kuom ango?’** | | | | | |
|  | | **Stove type – *use numbers/Kit* Kendo *e-ti gi namba*** | **The stove is used for….**  **Kendo itiyogo kuom….** | **English translations in this column please** | |
| **G2.1** | **Stove 1/ Kendo mokuongo** |  |  |  | |
| **G2.2** | **Stove 2/ Kendo mar ariyo** |  |  |  | |
| **G2.3** | **Stove 3/ Kendo mar adek** |  |  |  | |
| **G2.4** | **Stove 4/ Kendo mar angwen** |  |  |  | |

| **STOVES USED FOR COOKING/KENDO MA ITIYOGO E TEDO** | | | | | | | | | |
| --- | --- | --- | --- | --- | --- | --- | --- | --- | --- |
| **MAIN COOKING STOVE/KENDO MAOJATIIGO AHINYA** | | | | | | | | | |
| **G3.1** | **What type is your main cooking stove? [use codes as listed above]/ Kendo ma ijatiyogo ahinya en mane[tiigi nembni kaka ondik malo]** | | | | |  | | | |
| **G3.2** | **If non-listed stove, please describe/ka en maok ondik malo to yie iler** | | **English translations here please** | | | | | | |
| **G3.3** | **How many pots can be used on this stove at any one time?/Gin gauche adi ma inyalo tiigo e Kendoni dichiel?** | | | | |  | | | |
| **G3.4** | **If you paid for this stove, about how much did it cost? (Enter ‘0’ (zero) if three-stone fire or home-made) /Kane ichulo Kendoni, ne en pesa adi? (ndik nono ka en kendo mar kite adek ka tama olos nyaluo)** | | | | |  | | | |
| **G3.5** | **If you paid for this stove, about how long (months) do you think it will be before you have to replace the stove or the liner?/kane ichulo stove ni, iparo ni onyalo bedo kuom dweche adi kapok iloko machielo?**  ***If people tell you in years, multiply by 12 and insert the answer/ ka ji onyisi kuom higa, go kwan gi 12 aeto iket duoko.*** | | | | | | | |  |
| **G3.6** | **Has this stove needed repair?/Bende Kendoni osedwaro loso?** | | | **1 = Yes/Ee**  **2 = No/Ooyo (go to G3.9/Dhi e penjo mar G3.9)** | | | | |  |
| **G3.7** | **How many times have you had to have it repaired? (put ‘0’ if not repaired)/En didi ma isetere mondo olose? (Ket nono ka ok olose)** | | | | | | | |  |
| **G3.8** | **About how much (total) did repair cost?/Loso ne okawo pesa adi duto?**  ***[Enter ‘0’ (zero) if no cost and add up the costs if more than one repair][Ndik ‘0’ (Nono) ka onge nengo to iriw nengo duto ka okalo loso dichiel]*** | | | | | | | |  |
| **G3.9** | **Condition of stove/Chal mar Kendo**  ***Discuss with interviewee and examine the stove, and decide what on how you would classify stove/Wuo gi jaduok penjo kendo ingii* kendo *mondo ine kaka inyalo keto* kendo** | **1 = good condition/Ober**  **2 = Fair condition (Stove works properly but there are: Bent, loose, or broken parts; Some corrosion, Damaged grate, Pot or stove wobbles)/Ok orach ahinya (Kendo tiyo maber to nitie: kuone ma obam kata otur, nyal matin, agulu kata kendo yiengni)**  **3 = Poor condition (Stove is broken, does not work properly, bad corrosion, leakage of fuel, damaged grate, multiple cracks in lining, pieces missing)/Orach(Kendo otur, ok ti maber, nyal marach, mafuta chwer, buche mangeny e rachungi, gige moko onge)** | | | | | | |  |
| **G4.1** | **What times of day do you usually have this main stove alight?/En seche mage e odiochieng ma kendoni ojamoki?**  ***[Use 24 hour clock: hh:mm]/[Tiigi seche 24]*** | **First time lit/Saa ma omoke mokuongo** | | | | |  | | |
| **G4.2** |  | **Time goes out/turned off after first period used/Seche ma otho/onege bang seche ma otii kode ma okuongo** | | | | |  | | |
| **G4.3** |  | **Second time lit/Saa mar ariyo ma omoke** | | | | |  | | |
| **G4.4** |  | **Time goes out/turned off after second period used/Seche ma otho/onege bang seche ma otii kode ma okuongo** | | | | |  | | |
| **G4.5** |  | **Third time lit/Saa mar adek ma omoke** | | | | |  | | |
| **G4.6** |  | **Time goes out/turned off after third period used/Seche ma otho/onege bang seche ma otii kode ma okuongo** | | | | |  | | |
| **SECONDARY COOKING STOVE/KENDO MACHIELO MA ITEDOGO**  ***Only ask these questions if the person has more than one stove – otherwise go to G6.1/Penj penjoni mana ka ngani nigi* kendo *mokalo achiel-ka ok kamano dhi e penjo mar G6.1*** | | | | | | | | | |
| **G5.1** | **If you have a secondary cooking stove, what type is it? [Enter code = 11 if no secondary stove, and move to question G6.1]/Ka in gi gir tedo machielo, to en mane? [Ndik namba =11 ka onge gi kendo machielo, aeto idhi e penjo mar G6.1]** | | | | | | | **NUM** | |
| **G5.2** | **If non-listed secondary stove, please describe/ka en maok ondik malo to yie iler** | | | | **English translations here please** | | | | |
| **G5.3** | **How many pots can be used on this stove at any one time?/ Gin gauche adi ma inyalo tiigo e kendoni dichiel?** | | | | | | |  | |
| **G5.4** | **If you paid for this stove, about how much did it cost? (Enter ‘0’ (zero) if three-stone fire or home-made) /Kane ichulo kendon i, ne en pesa adi? (Ndik ‘0’ nono ka en kendo mar kite adek ka tama olos nyaluo)** | | | | | | |  | |
| **G5.5** | **If you paid for this stove, about how long (months) do you think it will be before you have to replace the stove or the liner? /kane ichulo kendoni, iparo ni onyalo bedo kuom dweche adi kapok iloko machielo?**  ***If people tell you in years, multiply by 12 and insert the answer/ ka ji onyisi kuom higa, go kwan gi 12 aeto iket duoko.*** | | | | | | |  | |
| **G5.6** | **Has this stove needed repair? /Bende kendoni osedwaro loso?** | | | **1 = Yes/Ee**  **2 = No/Ooyo (go to G5.9/Dhi e penjo mar G5.9)** | | | |  | |
| **G5.7** | **How many times have you had to having it repaired? /En didi ma isetere mondo olose? (Ket nono ka ok olose)** | | | | | | |  | |
| **G5.8** | **About how much (total) did repair cost? /Loso ne okawo pesa adi duto?**    ***[Enter ‘0’ (zero) if no cost and add up the costs if more than one repair] [Ndik ‘0’ (Nono) ka onge nengo to iriw nengo duto ka okalo loso dichiel]*** | | | | | | |  | |
| **G5.9** | **Condition of stove/Chal mar kendo** | **1 = good condition/Ober**  **2 = Fair condition (Stove works properly but there are: Bent, loose, or broken parts; Some corrosion, Damaged grate, Pot or stove wobbles) )/Ok orach ahinya (Kendo tiyo maber to nitie: kuone ma obam kata otur, nyal matin, agulu kata kendo yiengni)**    **3 = Poor condition (Stove is broken, does not work properly, bad corrosion, leakage of fuel, damaged grate, multiple cracks in lining, pieces missing) )/Orach(Kendo otur, ok ti maber, nyal marach, mafuta chwer, buche mangeny e rachungi, gige moko onge)** | | | | | |  | |

| **LOCATION OF CHILDREN WHEN COOKING IS TAKING PLACE/KAMA NYITHINDO NITIE SECHE MA ITEDO** | | | | | | |
| --- | --- | --- | --- | --- | --- | --- |
| **G6.1** | **When you are cooking, where usually is your youngest child?/Seche ma itedo, nyithinni matin ja bedo kanye?**  ***[this is the child about whom information is being collected in this study][ma e nyathi ma weche ikawo kuome e nonro]*** | | **1 = With you in the kitchen carried on your back/Koda e jikon ka tweye e toka**  **2 = With you in the kitchen but not on your back/koda e jikon to ok e toka**  **3 = In another room of the house/E rum machielo e ot**  **4 = Elsewhere/kamachielo** | | |  |
|  | | | | | **English translation here please** | |
| **G6.2** | **If the child is elsewhere, please describe and with whom:/ka nyathi ni kama chielo, yie iler togi ngama en go:** | **Where does the child stay?/Nyathi odak kanye?** | |  |  | |
| **G6.3** |  | **With whom does the child stay?/Nyathi odak gi nga?** | |  |  | |

| **H: USE OF STOVE FOR SPACE HEATING (if required)/TIYO GI KENDO E LIETO OT** | | | | | | |
| --- | --- | --- | --- | --- | --- | --- |
| **1 = Traditional (3-stone) fire/Mach mar kendo (kendo mar kite 3) (Kendo mar kite 3)** | | | **2 = Improved biomass stove**  **Specify:/ Kendo matiyo gi owuoyo Ler:** | | | |
| **3 = Traditional metal charcoal stove/Jiko** | | | **4 = Improved charcoal stove with ceramic liner/Jiko ma ochue iye** | | | |
| **5 = Ethanol stove /Kendo matiyo gi ethanol** | | | **6 = LPG stove/Stove mar gas** | | | |
| **7 = Kerosene wick stove/ Kendo mar kitambi** | | | **8 = Kerosene pressure stove/ Kendo ma ikudho** | | | |
| **9 = Electric stove/ Kendo mar sitima** | | | **10 = Other/Machielo** | | | |
| **11 = None/Onge** | | | **12 = Room heater (not used for cooking)/Raliet mar ot (Ok tedgo)** | | | |
| **H1** | **Do you ever use your stove for warmth in your house? /Bende ijatiyo gi kendo ni ne liet e odi?** | | | | **1 = Yes/Ee**  **2 = No/Ooyo (go to Question J1/Dhi e penjo mar J1)** |  |
| **H2.1** | **If Yes, which stove type do you use for warmth? [Use codes from table above]/ka ee, en kit kendo mane ma itiyogo e lieto ot? [Tiigi nambni man e listi man malo]** | | | | |  |
| **H2.2** | **If it is a non-listed stove, please describe/ka en maok ondik malo to yie iler** | | | **English translation in here please** | | |
| **H3** | **If Yes, how do you use the stove for heating/Ke ee, ere kaka itiyogi kendo ni ne liet?** | **1 = only while cooking/Mana e seche ma atedo**  **2 = during day time additional to cooking times/Odiochieng ka oriwore gi seche tedo**  **3 = during night time additional to cooking times/Otieno ka oriwore gi seche tedo**  **4 = during day time and night time additional to cooking times/Odiochieng gi otieno ka oriwore gi seche tedo** | | | |  |
| **H4.1** | **For about how many months of the year do you use the stove for warming the house in this way?/Gin kuom dweche adi e higa ma itiyogi stove ni e lieto ot gi yorni?** | | | | **During wet season (months)/E kinde ngich (Dweche)** |  |
| **H4.2** |  |  |  |  | **During dry season (months) )/E kinde ma piny liet (Dweche)** |  |

| **J: USE OF FUEL FOR ENTERPRISE AND SMALL BUSINESS/TIYO GI MACH NE KAMBI KATA OHALA MATINDO** | | | | | | | | | | | | |
| --- | --- | --- | --- | --- | --- | --- | --- | --- | --- | --- | --- | --- |
|  | | **1** | **Ethanol** | **2** | **Kerosene/Mafuta** | | | | **3** | **Bottled gas (LPG)/Gas** | |  |
|  |  | **4** | **Wood/Yien** | **5** | **Agri-Residues/Yugi mar puodho** | | | | **6** | **Charcoal/makaa** | |  |
|  |  | **7** | **Sawdust/Randa** | **8** | **Animal Dung/Owuoyo** | | | | **9** | **Mains electricity/Sitima** | |  |
|  |  | **10** | **Local generator** | **11** | **Battery** | | | | **12** | **Candles/Misuma** | |  |
|  |  | **13** | **Other/machielo** | **14** | **None/Onge** | | | |  |  | |  |
| **J1** | **Do you use fuel in your own house for an enterprise or small business?/Bende itiyo gi mach e odi ne kambi kata ohala matindo?** | | | | | | | **1 = Yes/Ee**  **2 = No/Ooyo (go to Question K1/Dhi e penjo mar K1)** | | |  |  |
| **J2.1** | **If Yes, which fuel do you use for enterprise/small business? [Use codes from table above]/[Ka ee. En mach mane ma itiyogo ne kambi kata ohala matindo? [Tiigi nambni man e listi man malo]** | | | | | | |  | | | |  |
| **J2.2** | **If non-listed fuel, please describe/ka en maok ondik malo to yie iler** | | | | | **English translation in here please** | | | | | |  |
| **J3** | **If Yes, for how many days each week do you usually use fuel in this way?/ Ka ee, en ndalo adi e juma ma itiyogi majni e yorni?** | | | | | | | **Days per week/Ndalo e juma** | | |  |  |
| **J4** | **About what fraction of all the fuel you use at home is for enterprise or small business?/Mach ma itiyogo marom nade e odi en mar kambi kata ohala matindo?** | | | | | | | **1 = None or very little/Onge kata matin ahinya**  **2 = Quarter/Robo**  **3 = Half /Nus**  **4 = Three-quarters/Robo adek**  **5 = Almost all/Chiegni te** | | |  |  |
| **J5** | **What is your enterprise or business?/Kambi kata ohandi en mane?** | | | | | | | **1 = Food for sale/Chiemb ohala**  **2 = Drink for sale/Amadha mar ohala**  **3 = Other (describe below)/machielo 9Ler)** | | |  |  |
| **J6** | **If it is a non-listed enterprise, please describe/ka en maok ondik malo to yie iler** | | | | | | **English translation in here please** | | | | |  |

| **K: USUAL FOOD COOKED and NUMBER OF PEOPLE FOR WHOM FOOD IS COOKED/KAR CHIEMO MA ITEDO togi KWAN JOMA ITEDO NEGI** | | | | | | | |
| --- | --- | --- | --- | --- | --- | --- | --- |
| **1 = Ugali/Kuon** | | **2 = Fish or shellfish/Rech** | | | | **3 = Meat/Ringo** | |
| **4 = Other meat/Ringo machielo** | | **5 = Vegetables/Alode** | | | | **6 = Beans/Oganda** | |
| **7 = Greens/Alode** | | **8 = Potatoes (Yams, Irish)/ Rabuon** | | | | **9 = Maize/Oduma** | |
| **10 = Chipatis/Chapat** | | **11 = Eggs/Tong** | | | | **12 = Porridge./Nyuka** | |
| **13 = Rice/mchele** | | **14 = Beverages (tea, coffee etc.)/Amadha(Chai, kahawa)** | | | | **15 = Other/Machielo** | |
| **K1.1** | **What foods /drinks do you usually cook each day in the wet season?/Gin kit chiemo mage ma ijatedo pile e kinde ngich**  **[Use code number from table above][Tiigi namba e listi mamalo]** | | | | **Food 1/Chiemo mokuongo** | |  |
| **K1.2** |  |  |  |  | **Food 2/Chiemo mar ariyo** | |  |
| **K1.3** |  |  |  |  | **Food 3/Chiemo mar adek** | |  |
| **K1.4** |  |  |  |  | **Food 4/Chiemo mar angwwen** | |  |
| **K2.1** | **What foods / drinks do you usually cook each day in the dry season? /Gin kit chiemo mage ma ijatedo pile e kinde ngich**  **[Use code number from table above] [Tiigi namba e listi mamalo]** | | | | **Food 1/Chiemo mokuongo** | |  |
| **K2.2** |  |  |  |  | **Food 2/Chiemo mar ariyo** | |  |
| **K2.3** |  |  |  |  | **Food 3/Chiemo mar adek** | |  |
| **K2.4** |  |  |  |  | **Food 4/Chiemo mar angwen** | |  |
| **K2.5** | **If it is a non-listed food, please describe/Ka en maok ondik malo to yie iler** | | | **English translation in here please** | | | |
| **K3.1** | **How many people do you usually cook for in the household – including yourself?/Gin ji adi ma ijatedonegi e ot ka oriwore kodi?**  ***[Do not include cooking done for other people as a business activity][Kik iriw tedo ne jomoko kaka ohala]*** | | **Male children less than 15 years/Nyithindo machwo man e bwo higni 15** | | | |  |
| **K3.2** |  |  | **Female children less than 15 years/Nyithindo manyiri man e bwo higni 15** | | | |  |
| **K3.3** |  |  | **Male adults 15 years and over/Joma chwo madongo higni 15 ka dhi malo** | | | |  |
| **K3.4** |  |  | **Female adults 15 years and over/Joma nyiri madongo higni 15 ka dhi malo** | | | |  |

**Thank you for answering those questions about your household. Now, I would like to talk to you a bit more about how you cook and what do you think about your current stove. We really appreciate your time. Your thoughts and ideas about cooking with your current stove are very important to us. The people who design the new stoves may be able to improve these stoves with your suggestions. There are no right or wrong answers to any of these questions, so please feel comfortable to speak freely. Erokamano kuom duoko penjogo kuom odi. Koro daher dhi nyime gi wuoyoni matin kuom kaka itedo togi gima iparo kuom kendoni. Waruako thulo mari ahinya. Pachi gi nendi kuom tedo gi kendoni masani en gima duongnwa ahinya. Joma loso kendo manyien nyalo medo loso kendogi gi pachi. Onge duoko maber kata marach kuom moro amora kuom penjogi, omiyo bed thuolo e wuoyo.**

| **L. QUALITATIVE INTERVIEW/PENJO** |
| --- |
| 1. **I would like for you to tell me about how you normally cook. Please tell me what you think about the stove that you currently use for cooking. *Probe: if they don’t talk about these issues, probe with the questions below./*Daher mondo inyisa kaka ijatedo. Yie inyisa gima ineno kuom kendo ma itiyogo sani. *Non: ka ok giwuoyo kuom wechegi, non gi penjo man e bwoye*** |
| - **How long have you been cooking on this stove?/Isetedo gi kendoni maron nade?** - **How long does it normally take you to cook a dish like ugali on your stove?/Kawiga thuolo marom nade mondo ited kuon e kendoni?** - **How easy or hard is it for you to use your current stove? Why?/Yot kata tek marom nade mondo itigi kendoni masani? Namgo?** - **How much time does it take you to light your stove? How easy or hard is it for you to light your stove? Why?/Kawi saa marom nade mondo imok kendoni** - **How easy or hard is it for you to keep the fire going in your stove? Why? ?/Yot kata tek marom nade mondo mach odhi nyime ka liel e kendoni? Nango?** - **Are you able to adjust the flame in your stove for cooking? How easy or hard is it for you to adjust the flame for cooking? Tell me more./Bende inyalo duoko mach chien kata tero nyime e kendoni? Yot kata tekni marom nade mondo iduoko mach chien kata tero nyime e kendoni?** - **Are you able to add fuel to the stove while cooking? How easy or hard is it to deal with the fuel while cooking? Tell me more./Bende inyalo medo mafuta/mach ka itedo? Yot kata tek marom nade medo mafuta/mach ka itedo? Med nyisa?** - **Does anyone else in your house cook on your stove?/Bende ngama chielo e odi tiyigi kendoni?** - **Have you ever taught anyone else how to cook on your stove? Could you tell me about how you taught them to use your stove?/Bende isepuonjo ngama chielo tedo gi kendoni? Bende inyalo nyisa kaka ne ipuojogi tedo gi kendoni?** - **Do you know anyone else that uses a different type of stove to cook than the stove that you have? What do you know about their stove? What do you think of their stove? /bende inge ngama chielo matiyogi kit kendo ma opogore gi mari? Ango ma ingeyo kuom kendogi? Ango ma iparo kuom kendogi?** - **Have you ever tried to use another type of stove than the one that you have?/Bende ise temo tiyo gi kendo ma opogore gi ma in go?** - **Is there another stove that you use for cooking? *If so, repeat question and probes for alternate stove*./Bende nitie kendo machielo ma itiyogg e tedo? *Ka ee, now penjo gi nono ne kendo mamoko*** |
| 1. **I would like to talk about the style, look, and shape of the cookstove. Please tell me what you think about when you look at your cookstove *(This is about physical aspects of the stove)*/Adwaro wuyo kuom kido gi chal mar kendo. Yie inyisa gima iparo ka ingiyo kendoni *(Ma en chal maoko mar kendoni)***   ***Probe: If they don’t talk about these issues, probe with the questions below. Non: ka ok giwuoyo kuom wechegi, non gi penjo man e bwoye*** |
| - **Did you like or dislike the shape/style of your current stove? Why?/Bende ne ihero kata ok ihero chal kata kido mar kendoni masani? Nango?** - **How does this shape/style of cookstove fit in your house?/Ere kaka chal kata kido mar kendoni donjo gi odi?** - **Please tell me about any concerns you have about the style, material or the different parts of this cookstove?Yie inyisa gima ineno kuom kido, gik ma otiigo e lose, kata gi kendoni mamoko?**   **-How about the height, size, shape? How about the space where you have to put the fuel?/ To borne, lachne, kata chwechne? To kama onego imedgo mafutakata mach?**   - **What would you change about this stove?/Ango ma inyalo loko kuom kendoni?** - **What do your family and friends think about your cookstove?/Ango ma joodi kata osiepe neon kuom kendoni?** |
| 1. **I want you to think about some of the health issues you may have experienced with your stove. Did you experience any of the following symptoms? (*Ask the checklist*) Did you experience any of the following symptoms with the new stove? (*Ask current stove first and check all the answers. Once you have completed the checklist ask the following question.)*/Adwaro ni ipar kuom weche korka ngima ma isebedogo gi kendoni. Bende ne ibedo gi achiel kuom ranyisigi? (*Penj gik man e listi*) Bende ne ibedo gi achiel kuom ranyisigi gi kendo manyien? (*Penj kendo manyien mokuongo kendo indik duoko duto. Ka isetieko gi listi, penj penjoni)*** |
| \| **Current Cookstove /Kendo manyien** \| **yes/ee** \| **no/ooyo** \| \| --- \| --- \| --- \| \| **Eyes water, burn, cry tears, Pi wangi chuer, wangi lungi, iyuak** \|  \|  \| \| **Cough, phlegm/Ahonda/okego** \|  \|  \| \| **Runny nose/Um mamol** \|  \|  \| \| **Chest tightness, pain/Kor maothung,malit** \|  \|  \| \| **Breathing problems/Chandruok kuom yueyo** \|  \|  \| \| **Likely to get burned/Inyalo wang** \|  \|  \| |
| 1. **What do you think about the smoke from your current stove?/Ango ma iparo kuom iro mawuok e kendoni masani?**   ***Probe: (if they don’t talk about these issues, probe with the questions below.) Non: ka ok giwuoyo kuom wechegi, non gi penjo man e bwoye*** |
| - **Tell me about the smoke from your stove. Do you notice any changes in your health when you are cooking? Nyisa kuom iro mawuok e kendoni. Bende ineno pogruok e ngimani ka itedo?** - **How do you feel that the amount of fuel this stove uses/ineno ango kuom kar yien ma kendoni tiyogo?** - **What are your concerns about smoke in your household/in gi weche mage kuom iro e odi?** |
| 1. **Money is hard to come by for many people. How much would you be willing to pay for this cookstove/Pesa tek yudo kuom ji mageny. Inyalo yie chulo pesa adi ne kendoni?**   ***Probe: (if they don’t talk about these issues, probe with the questions below.)/Non: ka ok giwuo kuom wechegi, non gi penjo man pinygi*** |
| - **How would you and your husband make the decision to spend money on a new cookstove/Ere kaka in kata jaodi nyalo ngado paro e tiyo gi pesa e kendo manyien?** - **What would be important for you to know when convincing your husband to spend money on a new cookstove? (Money saved on fuel? Time saved? Better or easier cooking for you? etc.)/Ango manyalo bedoni maduong ngeyo ka ikwayo jaodi mondo oti gi pesa e nyiewo kendo manyienni? (Pesa ma okan kuom yien? Seche ma ikano? Yor tedo maber kata mayot ne in?)** - **Where would the money come from to purchase a new cookstove/Pesa mar nyiewo kendo manyienni nyalo wuok kanye?** - **What would you have to give up in order to purchase a new cookstove/Ango ma inyalo weyo mondo mi inyiew kendo manyienni?** |
| 1. **Overall, how acceptable is the community to cooking on this type of stove? Is this a stove that your community would spend money on and cook with? *Probe: if they don’t talk about these issues, probe with the questions below./*Chutho, ere kaka jogweng oyie gi tedo gi kendoni? *Non: ka ok giwuo kuom wechegi, non gi penjo man pinygi*** |
| - **What would be the barriers to your community purchasing and cooking on this stove/Ango manyalo tamo jogweng’u nyiewo kendoni?** - **What would be the motivators to get people in your community to purchase and cook on this stove/Ango manyalo yuayo jogweng’u mondo onyiew kendoni?** - **How would you promote this stove to your family and friends/Ere kaka inyalo lando kendoni ne joodi kata osiepe?** |
| 1. **Is there anything else you would like to tell us about your experience with this cookstove/Bende nitie gima chielo madinyiswa kuom tiyoni gi kendoni?** |
|  |

PLEASE ADMINISTER CONJOINT QUESTIONNAIRE NOW.

| **M: Completion of Day 1 interview/Penj bang tieko ndalo 1** | | | | | |
| --- | --- | --- | --- | --- | --- |
| **M1** | **Interview end time/Seche ma penjo orume (use 24-hr clock/Tt gi seche 24)** | | |  | |
| **SET UP PERSONAL MONITOR FOR WOMAN AND INFANT AND ENSURE THAT IT IS COMFORTABLE TO WEAR AND THE WOMAN IS CLEAR ABOUT IT/KET RAMENY NE MIYO GI NYATHI KENDO INE NI RUAKE NI THUOLO KENDO OLERORE NE MIYO** | | | | | |
| **M2** | **Start time for setting up personal monitors/Saa mar chakruok keto ramaki** | | | |  |
| **M3** | **Confirm arrangement to return the following day at approximately the same time/non kendo chenro mar dok limbe kiny e seche machal gi ma** | **1 = Yes/Ee**  **2 = Woman does not wish to continue in the study/Miyo ok diher dhi nyime gi nonro**  **3 = Happy to take part, but unavailable at time requested/Mor gi bedo, to onge e seche ma okwa (describe plan for return in K3/ler chenro mar dok limbe e K3)** | | |  |
| **M4** | **If woman is not available for the return visit at the time requested, plan other arrangements/ka miyo onge e dok limbe e seche ma okwa, pang yore mamoko**  ***Discuss with supervisor if needed/Wuoye gi jatelo kanyalore.*** | **When is she available and what has been arranged/Oyudore kar ango to ango ma opangi?** | **English translation in here please** | | |
| **Thank you very much for your time and assistance/Erokamano kuom thuolo gi kony mari.** | | | | | |

**WECHE NE JACHIW PENJO:**

**kapok iwuok, chak iler ne miyo ni nyathi ok onego pogre gi badge gi mpira. Penj ka ne owinjo maber gima onego tim gi ma gotieno, ka oluokore kata oluoko nyathi, togi ka odhi oko mar ot.**

**jiwe ni odhi nyime gi tije mapile, kendo kik otim gima opogore gi pile.**

Household Focus Group Discussion Topic Guide

**Introduction:**

We really appreciate your time and thank you for testing the stove. We would like to ask you as a group some questions about the stove. Your thoughts and ideas about cooking with the stove are really important to us. The people who design the stove may be able to make changes and improve the stove with your suggestions. Remember that you are all here today to share your opinions, so please be comfortable telling us what you think and how you feel. It is okay to have a different opinion than someone else in the group. Everyone’s opinion is equally important.

1. **What did you think of cooking on this stove?**
2. **How does the stove you were cooking on compare to your old stove?**

**2A) Now that you have had a chance to cook on another stove how does it compare with the other stoves you have tested? Which features are most important to you? Which stove do you prefer and why?**

**2B) Please rank the stoves you have cooked on according to the following:**

- 1. Comfort and ease of use
  2. Cooks traditional foods well
  3. Heat is even and no flame ups
  4. Speeds up cooking
  5. Pots fit nicely
  6. Cost
  7. Fuel efficacy
  8. Smoke reduction
  9. Style

1. **When did you not cook on the new stove? Why? When did you cook on both stoves? Why?**
2. **What would you be willing to pay for a new stove?**
3. **What else do you want to tell us?**

We really appreciate all of your time today.

The information you have provided us is very important

Thank you so much!

Household Focus Group Discussion Topic Guide Households at Follow-up [English version]

**Introduction:**

We really appreciate your time and thank you for testing the stove. You have had your stove now for a longer period of time, so you have had more time to use the stove on a daily basis. We would like to ask you as a group some questions about the stove. Your thoughts and ideas about cooking with the stove are really important to us. Remember that you are all here today to share your opinions, so please be comfortable telling us what you think and how you feel. It is okay to have a different opinion than someone else in the group. Everyone’s opinion is equally important. You have had a stove for about

**What do you think of cooking on this stove?**

**How does the stove you were cooking on compare to your old stove? Have you noticed any changes in the amount of time you spend cooking? Have you noticed any changes in the amount of fuel that you use? Has anything else changed about the way that you cook or use this stove?**

**2A) Now that you have had a chance to cook on another stove how does it compare with the other stoves you have tested? Which features are most important to you? Which stove do you prefer and why?**

**When did you not cook on the new stove? Why? When did you cook on both stoves? Why?**

**Has anyone else ever asked you any questions about your stove? What kinds of questions did they ask you? What do people in your community think about this type of stove? Has anyone else ever used your stove? What did they**

**What else do you want to tell us?**

We really appreciate all of your time today.

The information you have provided us is very important

Thank you so much!

Ventilation Assessment Form

Household ID: **________________** Respondent Name: **______________________** Enumerator/Supervisor Initials: **______**

Children

Are children under five years of age usually present in the room while cooking is occurring? □ Yes □ No

If yes, what are their ages: 1. _____________ 2. _____________ 3. _____________ 4. _____________ 5. _____________

Housing

Is the cooking area in the same location as the living area? □ Yes □ No

How is the ventilation of your cooking area? (Please circle one) Good Moderate Poor

Is there a gap between roof and walls? □ Yes □ No

Can air pass through the kitchen or cooking area walls? □ Yes □ No

Material of roof _________________________ Material of walls _________________________

Number of doors __________ Number of windows ___________ Number of eaves spaces _________

Do you have a special vent? □ Yes □ No If yes, what kind of special vent do you have? __________________________

At the time of cooking, were your windows open? □ Yes □ No

At the time of cooking, were your doors open? □ Yes □ No

***Drawing Instructions***: *The box below represents a bird’s eye view of the structure where [clean burning stove] is located and cooking occurs most often. This may be either a separate kitchen structure or a cooking area within a larger structure that includes other rooms. Using the symbols below, indicate the locations of the stove, existing windows, doors, indoor walls, ventilation added with [clean burning stove] installation, children’s beds, and the particle monitoring device. Indicate children’s age (in years) within the circles of their symbol, using <1 for infants.*

Traditional Stove Upesi Jiko Window Door Indoor Wall Added Ventilation Male Child’s Bed Female Child’s Bed Particle Monitor

Dimensions: _______________ (height) _________________ (length) ________________ (width)

Stove-related air pollution exposure assessment [English version]

Questionnaire

(To be administered at the end of the 48-hr air sampling)

**Instructions:** This form is to be completed by the household interviewer or note taker at the end of each new cookstove two week session.

| **A: Reinforcing of the identification** | | |
| --- | --- | --- |
| **A1.2** | Study household ID number |  |
| **A2.2** | Neighbourhood and house location |  |
| **A3.2** | Interviewer’s name [ID] (not interviewee) |  |
| **A4.2** | Date of interview (Day/Month /Year) | __ __/ __ __ /__ __ __ __ |
| **A5.2** | Starting time of interview (within24hours) | __ __:__ __ |

| **N: Collecting the measure equipment** | | | | | |
| --- | --- | --- | --- | --- | --- |
| **N1** | Time when the **equipment was switched off** (within 24 hours) | | | __ __ / __ __ | |
| **N2** | Look and ask about the place of the **monitor** when you arrived | 1 = Woman was wearing it  2 = Woman was holding it  3 = Not with the woman  *(describe below where it was found)* | | |  |
| **N3** | If answer-3 , where was the **monitor?** | | **Please translate into English in here** | | |
| **N4** | Ask her if she had managed to keep the monitor with her … | 1 = Yes, all the time  2 = most of the time  3 = about half the time or less | | |  |
| **Ask the mother about the CO tube with the child: taking off, covering, and place where she put it** | | | | | |
| **N5** | Look and ask about the place of the **CO tube** when you arrived | 1 = Child was wearing it (go to N7)  2 = not with the child | | |  |
| **N6** | If N5 answer = 2, describe location of tube | **Please translate into English in here** | | | |
| **N7** | Ask the mother if she managed to keep the **CO tube** with the child… | 1 = Yes, all the time  2 = most of the time  3 = about half the time or less | | |  |

| **P: CONDITION OF FUEL** | | | |
| --- | --- | --- | --- |
| **P1** | Was the main fuel you used whilst the monitoring was taking place completely dead (fully dried) or 'green' (freshly cut)?  *[Green wood is wood that has just been cut down from a living tree and* ***still contains sap****]* | 1 = Did not use biomass  2 = Completely dead  3 = ‘Green’ |  |
| **P2** | How dry was the fuel you used while the monitoring was taking place?  *[This question is to do with* ***wetness*** *caused by* ***rain****, not whether the wood* ***still contains sap****]* | 1 = Wet  2 = Moderately dry  3 = Very dry  **4 = Did not use biomass or charcoal** |  |
| **P3** | If you gathered any fuel, how difficult was it to collect? | 1 = Did not gather any  2 = Very difficult  3 = Difficult  4 = Neither difficult nor easy  5 = Easy  6 = Very easy |  |

| **Q: ABOUT FUELS AND STOVES** | | | | | | | | | | |
| --- | --- | --- | --- | --- | --- | --- | --- | --- | --- | --- |
| **STOVES** | | | | | | | | | | |
|  | **1** | | Three-stone fire | | | | **2** | Improved biomass stove  Specify: | | |
|  | **3** | | Metal charcoal stove | | | | **4** | Improved charcoal stove | | |
|  | **5** | | “Ethanol” stove | | | | **6** | Gas stove | | |
|  | **7** | | Kerosene **wick** stove | | | | **8** | Kerosene **pressure** stove | | |
|  | **9** | | Electric stove | | | | **10** | Other | | |
|  | **11** | | None | | | |  |  | | |
| **FUELS** | | | | | | | | | | |
|  | | **1** | | “Ethanol” | **2** | Kerosene | | | **3** | Bottled gas |
|  |  | **4** | | wood | **5** | Agri-residues | | | **6** | Charcoal |
|  |  | **7** | | Saw-dust | **8** | Animal dung | | | **9** | Electricity |
|  |  | **10** | | Local Generator | **11** | Battery | | | **12** | Candles |
|  |  | **13** | | Other |  |  | | |  |  |

| Q: Cooking sessions ***– (please fill in*** *as many meals as required* ***– as in Q1 below )*** | | | | | |
| --- | --- | --- | --- | --- | --- |
| **Q1.1** | How many meals did you cook while the monitors were running? | | | |  |
| **Q1.2** | **How much of the time did you have the stove alight?** | | | **1=Just for cooking**  **2= More than half the daytime**  **3= All day but not whilst asleep**  **4 = All day and all night** |  |
| **Q2** | About what time did you light **(or rekindle)** the stove for the **first cooking session** after the monitors were started ?(24hr) | | | | _ _ / _ _ |
| **Q3** | About how long did you keep the stove alight for the first cooking session? | | | | ….hr. …min |
| **Q4** | What **stove** did you use for the first cooking session? [**Use stoves list**] | | | |  |
| **Q5** | What **fuel** did you use for the first cooking session? [**Use fuels list**] | | | |  |
| **Q6.1** | **How many people did you cook for in the first cooking session?** | Male children (less than 15 years) | | |  |
| **Q6.2** |  | Female children (less than 15 years) | | |  |
| **Q6.3** |  | Male adults (15 years and over) | | |  |
| **Q6.4** |  | Female adults (15 years and over) | | |  |
| **Q7** | About what time did you light **(or rekindle)** stove for the **second cooking session**? (24hr) | | | | _ _ / _ _ |
| **Q8** | About how long did you keep the stove alight for the **second cooking session**? | | | | ….hr. …min |
| **Q9** | What **stove** did you use for the **second cooking session**? | | | |  |
| **Q10** | What **fuel** did you use for the **second cooking session**? | | | |  |
| **Q11.1** | **How many people did you cook for in the second cooking session?** | Male children (less than 15 years) | | |  |
| **Q11.2** |  | Female children (less than 15 years) | | |  |
| **Q11.3** |  | Male adults (15 years and over) | | |  |
| **Q11.4** |  | Female adults (15 years and over) | | |  |
| **Q12** | About what time did you light **(or rekindle)** the stove for the **third cooking session**? (24hr) | | | | _ _ / _ _ |
| **Q13** | About how long did you keep the stove alight for the **third cooking session**? | | | | ….hr. …min |
| **Q14** | What **stove** did you use for the **third** **cooking session**? | | | |  |
| **Q15** | What **fuel** did you use for the **third cooking session**? | | | |  |
| **Q16.1** | **How many people did you cook for in the third cooking session?** | Male children (less than 15 years) | | |  |
| **Q16.2** |  | Female children (less than 15 years) | | |  |
| **Q16.3** |  | Male adults (15 years and over) | | |  |
| **Q16.4** |  | Female adults (15 years and over) | | |  |
| **Q17** | About what time did you light **(or rekindle)** the stove for the **fourth cooking** **session?** | | | | _ _ / _ _ |
| **Q18** | How long did you keep the stove alight for the **fourth cooking session**? | | | | ….hr. …min |
| **Q19** | What **stove** did you use for the **fourth cooking session**? | | | |  |
| **Q20** | What **fuel** did you use for the **fourth cooking session**? | | | |  |
| **Q21.1** | **How many people did you cook for in the fourth cooking session?** | | Male children (less than 15 years) | |  |
| **Q21.2** |  |  | Female children (less than 15 years) | |  |
| **Q21.3** |  |  | Male adults (15 years and over) | |  |
| **Q21.4** |  |  | Female adults (15 years and over) | |  |

| **FUELS** | | | | | | | | | | | |
| --- | --- | --- | --- | --- | --- | --- | --- | --- | --- | --- | --- |
|  | **1** | | “Ethanol” | 2 | Kerosene | | | 3 | | Bottled gas | |
|  | **4** | | wood | 5 | Agri-residues | | | 6 | | Charcoal | |
|  | **7** | | Saw-dust | 8 | Animal dung | | | 9 | | Electricity | |
|  | **10** | | Local Generator | 11 | Battery | | | 12 | | Candles | |
|  | **13** | | Other |  |  | | |  | |  | |
| **R: LIGHTING** | | | | | | | | | | | |
| **R1** | | What was the main fuel you used as lighting during the study period? [use the number in the list above] | | | | | | |  | | |
| **R2** | | If lighting fuel is not listed, please describe | | | | **Please translate into English in here** | | | | | |
| **S: Employment and other uses** | | | | | | | | | | | |
| **S1** | | Did you use fuel for your enterprise or small business during the study time? | | | | | 1 = Yes  2 = No *(Go to T1)* | | | |  |
| **S2** | | If *Yes,* what fraction of the fuel you use at home did you use for your enterprise? | | | | | 1 = None or very little  2 = Quarter  3 = Half  4 = Three-quarters  5 = Almost all | | | |  |

| **T: Remarks from the interviewee** | | | | | | | |
| --- | --- | --- | --- | --- | --- | --- | --- |
| **T1** | Have you noticed any differences between your day since my visit and the other days without monitoring? | | | | | 1 = Yes  2 = No (go to T3) |  |
|  | | | | | | **English translations in this column please** | |
| **T2** | Please describe the differences | | | | |  | |
| **T3** | Was the monitor disturbed during the study period (e.g.: handled by children or moved away ) | | | | |  | |
| **T4** | Are there any comments you would like to make?  *Please do not prompt – continue on reverse if needed* | | | | |  | |
| **U: About the weather and the interview** | | | | | | | |
| **U1** | | The weather during the study period | Hot | | 1 = Hot  2 = Warm  3 = Cool | |  |
|  |  |  | Humidity | | 1 = Very humid  2 = Moderately humid  3 = Dry | |  |
|  |  |  | Rain | | 1 = Heavy rain  2 = Light rain  3 = No rain | |  |
|  |  |  | Wind | | 1 = Strong wind  2 = Light wind (breeze)  3 = No wind | |  |
| **End of the study period and remarks from the interviewer** | | | | | | | |
| **U2** | | Observations from surveyor on anything which you may feel affect the results: | | **Please translate into English in here** | | | |
|  | | | | | | | |

**Thank you for answering those questions about your household. Now, I would like to talk to you a bit more about your individual experience with using this stove. We really appreciate your time, and thank you for testing the stove. Your thoughts and ideas about cooking with this stove are very important to us. The people who designed the stove may be able to improve the stove with your suggestions. There are no right or wrong answers to any of these questions, so please feel comfortable to speak freely**

**You have now cooked food on the cookstove. Please tell me what you think about cooking on this cookstove. (***This is about the experience of cooking on the stove)*

*Probe: (If they don’t talk about these issues, probe with the questions below.)*

What did you think of the instructions that we gave you on how to use the stove? Was there anything that was unclear?

What differences did you experience when cooking on this cookstove as compared to your old cookstove?

What things were similar between this cookstove and your current cookstove when you were cooking?

What were features of this cookstove that you really valued when cooking? Why?

What were features of the cookstove that you did not like when cooking? Why?

Are there other things that you liked/disliked about the stove?

How much time did it take you to light this stove? How easy or hard was it for you to light this cookstove? Why?

How easy or hard was it for you to keep the fire going in the this cookstove? Why?

How easy or hard was it for you to cook this cookstove? Why?

Describe for me anything you noticed about the speed of your cooking? Were you able to prepare your dishes faster or slower than when you cook on your current stove? Why?

Were you able to adjust the flame for cooking? How easy or hard was it to adjust the flame for cooking? Tell me more.

Were you able to add fuel to the stove while cooking? How easy or hard was it to deal with the fuel while cooking? Tell me more.

Did you have to change how you cooked when using this new cookstove? Tell me more.

Did you use your three-stone stove to cook at all over the past two weeks? When did you use another stove? What influenced your decision to use another stove?

Does anyone else in your house cook on your current cookstove? If yes, tell me how you taught them to use this stove. How easy or hard it was for you to teach them. Was there anything about the stove that they did not understand?

**I would like to talk about the style, look, and shape of the cookstove. Please tell me what you first thought when you looked at the cookstove?** *(This is about physical aspects of the stove)*

*Probe: (If they don’t talk about these issues, probe with the questions below.)*

How similar or different does the new stove look to your old stove?

Did you like or dislike the shape/style of the stove when you first saw it? Why?

Now that you have cooked on the cookstove what do you think about the shape/style and look of the stove?

How does this shape/style of cookstove fit in your house?

Please tell me about any concerns you have about the style, material or the different parts of this cookstove?

-How about the height, size, shape? How about the space where you have to put the fuel?

What would you change about this stove if you were to keep it?

What do your family and friends think about this style of cookstove?

**I want you to think about some of the health issues you may have experienced with your current stove. Did you experience any of the following symptoms? (***Ask the checklist***) Did you experience any of the following symptoms with the new stove? (***Ask current stove first and check all the answers. Then ask the same of the new stove. Once you have completed the checklist ask the following question.)*

| **Current Cookstove** | **yes** | **no** | **New Cookstove** | **Yes** | **no** |
| --- | --- | --- | --- | --- | --- |
| Eyes water, burn, cry tears |  |  | Eyes water, burn, cry tears |  |  |
| Cough, phlegm |  |  | Cough, phlegm |  |  |
| Runny nose |  |  | Runny nose |  |  |
| Chest tightness, pain |  |  | Chest tightness, pain |  |  |
| Breathing problems |  |  | Breathing problems |  |  |
| Likely to get burned |  |  | Likely to get burned |  |  |

**What do you think about the smoke from the new stove as compared to your current stove?**

*Probe: (Ask specifically the questions below after the woman has answered you question.) Ensure that respondent knows you are asking them to compare the trial stove with the stove they had traditionally used, not with a previous trial stove that had been previously tried.*

Tell me about the smoke from the new stove? Did you notice any symptoms when cooking on the new stove? Tell me about them.

How does it compare to your current stove?

How do you feel that the amount of fuel this stove uses compares to your current stove?

What are your concerns about smoke in your household?

**Money is hard to come by for many people. How much would you be wiling to pay for this cookstove?**

*Probe: (Ask specifically the questions below after the woman has answered you question.)*

How would you and your husband make the decision to spend money on a new cookstove?

What would be important for you to know when convincing your husband to spend money on a new cookstove? (Money saved on fuel? Time saved? Better or easier cooking for you? etc.)

Where would the money come from to purchase a new cookstove?

What would you have to give up in order to purchase a new cookstove?

**Overall, how acceptable would the community be to purchasing and cooking on this stove? Is this a stove that your community would spend money on and cook with?**

What would be the barriers to your community purchasing and cooking on this stove?

What would be the motivators to get people in your community to purchase and cook on this stove?

What would you tell your family and friends about this stove? How would you describe it to them? How would you promote this stove to your family and friends?

**Is there anything else you would like to tell us about your experience with this cookstove?**

**We really appreciate your time. The information you have given us is very important. Thank you very much!**

**MENTION NEXT VISIT**
