# Supplementary material for: Effectiveness of Six Improved Cookstoves in Reducing Household Air Pollution and Their Acceptability in Rural Western Kenya
Source: PLoS One. 2016 Nov 15;11(11):e0165529. doi: 10.1371/journal.pone.0165529 (PMC5112915; doi:10.1371/journal.pone.0165529)
Supplement: S3 File — (PDF) [file pone.0165529.s003.pdf]

**Respondent name:** **Respondent ID:** **Village name:**

**DAY 1**

**Date:**

| Cooking period/**kinde mag tedo** | Start Time/**saa ma tedo ochakore** | End time/**saa ma otieke tedo** | Stove(s) used for cooking/**kende motigo etedo** | What was cooked during the cooking period?/**ango mane otedi ekinde mag tedo** | # of people cooked for/**kwan joma notednegi** |
|---|---|---|---|---|---|
| Breakfast/**chai mokinyi** | | | o 3-stone/**kite adek**<br>o Study stove/**kendo mar nonro**<br>o Other/**machielo** specify/**yang ka machielo_______** | | |
| Between breakfast and lunch/**ekind chai mokinyi gi saa lanch** | | | o 3-stone/**kite adek**<br>o Study stove/**kendo mar nonro**<br>o Other/**machielo** specify/**yang ka machielo_______** | | |
| Lunch/**lanch** | | | o 3-stone/**kite adek**<br>o Study stove/**kendo mar nonro**<br>o Other/**machielo** specify/**yang ka machielo_______** | | |
| Between Lunch and supper/**ekind lanch gi sapa** | | | o 3-stone/**kite adek**<br>o Study stove/**kendo mar nonro**<br>o Other/**machielo** specify/**yang ka machielo_______** | | |
| Supper/**sapa** | | | o 3-stone/**kite adek**<br>o Study stove/**kendo mar nonro**<br>o Other/**machielo** specify/**yang ka machielo_______** | | |
| Other/**mamoko** | | | o 3-stone/**kite adek**<br>o Study stove/**kendo mar nonro**<br>o Other specify/**yang ka machielo_______** | | |

**Lamp use: taya motigo**

| **Start Time/saa ma omoke** | **End time/saa ma osime** |
|---|---|
| | |
| | |
| | |

**Respondent name:** **Respondent ID:** **Village name:**

**DAY 2**
**Date:**

| Cooking period/**kinde mag tedo** | Start Time/**saa ma tedo ochakore** | End time/**saa ma otieke tedo** | Stove(s) used for cooking/**kende motigo etedo** | What was cooked during the cooking period?/**ango ma ne otedi ekinde mag tedo?** | # of people cooked for/**kwan joma ne otednegi** |
|---|---|---|---|---|---|
| Breakfast/**chai mokinyi** | | | o 3-stone/**kite adek**<br>o Study stove/**kendo mar nonro**<br>o Other/**machielo** specify/**yang ka machielo**_______ | | |
| Between breakfast and lunch/**ekind chai mokinyi gi saa lanch** | | | o 3-stone/**kite adek**<br>o Study stove/**kendo mar nonro**<br>o Other/**machielo** specify/**yang ka machielo**_______ | | |
| Lunch/**lanch** | | | o 3-stone/**kite adek**<br>o Study stove/**kendo mar nonro**<br>o Other/**machielo** specify/**yang ka machielo**_______ | | |
| Between Lunch and supper/**ekind** lanch gi sapa | | | o 3-stone/**kite adek**<br>o Study stove/**kendo mar nonro**<br>o Other/**machielo** specify/**yang ka machielo**_______ | | |
| Supper/**sapa** | | | o 3-stone/**kite adek**<br>o Study stove/**kendo mar nonro**<br>o Other/**machielo** specify/**yang ka machielo**_______ | | |
| Other/**mamoko** | | | o 3-stone/**kite adek**<br>o Study stove/**kendo mar nonro**<br>o Other/**machielo** specify/**yang ka machielo**_______ | | |

**Lamp use:**

| Start Time/**saa ma omoke** | End time/**saa ma osime** |
|---|---|
| | |
| | |
| | |

**Respondent name:** **Respondent ID:** **Village name:**

**DAY 3**

**Date:**

| Cooking period/**kinde mag tedo** | Start Time/**saa ma tedo ochakore** | End time/**saa ma otieke tedo** | Stove(s) used for cooking/**kende motigo etedo** | What was cooked during the cooking period?/**ango mane otedi ekinde mag tedo** | Number of people cooked for/**kwan joma notednegi** |
|---|---|---|---|---|---|
| Breakfast/**chai mokinyi** | | | o 3-stone/**kite adek**<br>o Study stove/**kendo mar nonro**<br>o Other/**machielo** specify/**yang ka machielo_______** | | |
| Between breakfast and lunch/**ekind chai mokinyi gi saa lanch** | | | o 3-stone/**kite adek**<br>o Study stove/**kendo mar nonro**<br>o Other/**machielo** specify/**yang ka machielo_______** | | |
| Lunch/**lanch** | | | o 3-stone/**kite adek**<br>o Study stove/**kendo mar nonro**<br>o Other/**machielo** specify/**yang ka machielo_______** | | |
