# Supplementary material for: Effectiveness of Six Improved Cookstoves in Reducing Household Air Pollution and Their Acceptability in Rural Western Kenya
Source: PLoS One. 2016 Nov 15;11(11):e0165529. doi: 10.1371/journal.pone.0165529 (PMC5112915; doi:10.1371/journal.pone.0165529)
Supplement: S4 File — (PDF) [file pone.0165529.s004.pdf]

VillageID	id	age	latitude	longitude	altitude	round	stove_type	PM_mg_m3	mean_cok
33	4330101	26	-0.208091667	34.99321833	1161.8	0	STO	0.945	10.9
33	4330101		-0.208273333	34.99348333	1171.1	1	EZO	0.396	3.5
33	4330101		-0.208765	34.994175	1165.7	2	PRA	0.491	4.3
33	4330101		-0.208266667	34.99356167	1137.9	3	ENV	0.869	10.7
33	4330101		-0.208265	34.99356167	1162.7	4	ECH	0.361	3.4
33	4330101		-0.210501667	34.99418333	1163	5	RKT	0.237	2.9
33	4330101		0	0	0	6	PHI	0.995	10.3
33	4330201	42	0	0	0	0	STO	1.963	13.9
33	4330201		-0.203975	34.997535	1173.5	1	ENV	0.528	6
33	4330201		-0.203933333	34.99751167	1166.1	2	EZO	1.047	6.8
33	4330201		-0.203938333	34.99747333	1167.6	3	PRA	0.58	3.8
33	4330201		-0.204051667	34.99715333	1256.9	4	ECH	0.553	5.6
33	4330201		-0.203813333	34.99746333	1169.1	5	RKT	1.027	5.9
33	4330201		-0.203885	34.99746667	1171.9	6	PHI	0.206	2.7
33	4330301	24	-0.2147	34.99390667	1177	0	STO	1.492	25.7
33	4330301		-0.214746667	34.99394333	1161.1	1	PRA	0.999	47.4
33	4330301		-0.214786667	34.99381	1167.1	2	PHI	0.848	11.1
33	4330301		0	0	0	3	ENV	4.034	41.7
33	4330301		-0.214748333	34.99383833	1155.3	4	ECH	1.597	33.2
33	4330301		-0.214731667	34.993825	1150.8	5	EZO	2.851	51.2
33	4330401	32	-0.218718333	35.000475	1143.2	0	STO	0.165	0.8
33	4330401		-0.21933	35.000275	1187.2	1	PHI	0.416	6.4
33	4330401		-0.217961667	34.99984667	1157.2	2	ENV	0.228	
33	4330401		-0.218736667	35.00048	1159.4	3	EZO	0.652	4.5
33	4330401		-0.217503333	34.99977333	1167.1	4	PRA	0.13	2
33	4330401		-0.218693333	35.000455	1161.8	5	ECH	0.116	1.6
33	4330401		-0.218636667	35.00049167	1165.4	6	RKT	0.183	2.2
33	4330501	23	-0.210896667	34.989195	1162.5	0	STO	0.195	3.9
33	4330501		-0.18052	34.99328	8350.8	1	PRA	0.103	1.7
33	4330501		-0.210938333	34.98919	1160	2	PHI	0.283	5.1
33	4330501		-0.210926667	34.98915667	1185.3	3	EZO	0.3	4.2
33	4330501		-0.21093	34.98921667	1157.7	4	RKT	0.371	
33	4330501		0	0	0	5	ECH	0.438	3.5
33	4330601	21	-0.212635	35.00046333	1153.2	0	STO	0.9	16.1
33	4330601		-0.212326667	35.00072833	1166.2	1	PRA	0.603	13.2
33	4330601		-0.212525	35.00066833	1161.3	2	PHI	1.167	32.4
33	4330601		-0.212498333	35.000505	1158.5	3	EZO	0.732	18.3
33	4330601		-0.212601667	35.00052333	1165.9	4	RKT	0.274	4
33	4330601		-0.21254	35.00051167	1154.5	5	ECH	0.487	5.9
33	4330701	34				0	STO	1.189	8.6
33	4330701		-0.209706667	34.98639167	1184.1	1	EZO	1.365	14.9
33	4330701		-0.209978333	34.98584333	1150.8	2	PRA	0.412	4.1
33	4330701		-0.209946667	34.98594167	1157.8	3	PHI	0.26	2.6
33	4330701		-0.20977	34.98613167	1150.4	4	ECH	0.505	6.1
33	4330701		-0.209975	34.98582	1154.8	5	ENV	0.232	2.7
33	4330701		-0.209988333	34.985785	1159.1	6	RKT	0.538	6.9

33	4330801	29	-0.209006667	34.98201167	1165.4	0	STO	1.885	35.2
33	4330801		-0.20905	34.98193	1162.5	1	EZO	0.933	16.6
33	4330801		-0.20903	34.98200167	1166.2	2	PRA	3.426	35
33	4330801					3	PHI	0.649	
33	4330801		-0.20904	34.98207	1154.5	4	ECH	1.416	16
33	4330801		-0.208968333	34.98204667	1153.8	5	ENV	0.479	7.9
33	4330801		-0.208878333	34.98226167	1153.5	6	RKT	0.235	3.8
33	4330901	23	-0.218373333	34.98929333	1148.8	0	STO	1.332	12.9
33	4330901		-0.218388333	34.98941333	1130.1	1	PRA	1.052	14
33	4330901		-0.21841	34.98930167	1152.9	2	PHI	0.264	3
33	4330901		-0.21852	34.98931833	1169	3	EZO	0.724	9.6
33	4330901		-0.218256667	34.98932	1161.9	4	RKT	1.408	16
33	4330901		-0.21844	34.98930333	1161.6	5	ECH	1.15	11.6
33	4331001	18				0	STO	0.134	1
33	4331001		-0.21403	34.99970167	1163.7	1	PRA	0.188	2.9
33	4331001		-0.214138333	35.00001333	1164.1	2	PHI	0.237	3.6
33	4331001		-0.214048333	34.99973	1164	3	EZO	0.197	2
33	4331001		-0.214101667	35.00001833	1165.5	4	RKT	0.074	0.9
33	4331001		-0.213995	34.99969667	1164.5	5	ECH	0.235	2.6
33	4331101	26				0	STO	0.542	3.2
33	4331101		-0.213021667	34.987715	1164.6	1	EZO	0.41	2.9
33	4331101					2	PRA	0.191	3.2
33	4331101		-0.212853333	34.98774333	1159.4	3	PHI	0.213	1.5
33	4331101		-0.212826667	34.98772167	1171.3	4	ECH	0.314	2.4
33	4331101		-0.2128	34.98777833	1157	5	RKT	0.329	2.9
33	4331201	39	-0.206723333	34.994715	1161.7	0	STO	0.185	2
33	4331201		-0.206745	34.99464167	1174.3	3	ENV	0.911	7.3
33	4331201		-0.2067	34.99465	1159.2	4	PRA	0.118	
33	4331201		-0.206655	34.99464833	1163.7	5	RKT	0.287	3.6
33	4331201		-0.206798333	34.99468667	1166.7	6	PHI	0.08	2
33	4331301	17	0	0	0	0	STO	0.27	1.9
33	4331301		-0.209623333	34.99043333	1163.3	1	EZO	0.121	4
33	4331301		0	0	0	2	PRA	0.538	5.6
33	4331401	25	-0.208136667	34.98845667	1148.1	0	STO	0.513	4
33	4331401		-0.209565	34.95862	1145.8	1	ENV	0.412	4.9
33	4331401		-0.208148333	34.98833	1160.7	2	EZO	0.225	2.3
33	4331401		-0.208091667	34.98838	1153.5	3	PRA	0.201	1.8
33	4331401		-0.208001667	34.98872667	1164.8	4	PHI	0.219	1.5
33	4331401		-0.208106667	34.98843167	1161.3	5	RKT	0.099	1
33	4331501	35	-0.213793333	34.98681167	1156.6	0	STO	0.524	8.3
33	4331501		-0.213775	34.98682667	1161.6	1	ENV	0.162	5.2
33	4331501		-0.21378	34.98682333	1156	2	EZO	0.381	4.6
33	4331501		-0.213746667	34.98684333	1154.3	3	PRA	0.088	1.4
33	4331501		-0.21374	34.98693167	1156.2	4	PHI	0.204	2.6
33	4331501		-0.213705	34.98682333	1165.4	5	RKT	0.242	2.5
33	4331601	32				0	STO	0.324	2.7
33	4331601		-0.219705	34.98616833	1160.5	1	PRA	0.199	1.6

33	4331601		-0.219686667	34.98612333	1166.2	2	PHI	0.184	1.1
33	4331601		-0.219671667	34.98623167	1157	3	EZO	0.16	1.7
33	4331601		-0.219918333	34.98607	1157	4	RKT	0.216	2.3
33	4331601		-0.219625	34.98621	1154.3	5	ECH	0.474	4.4
33	4331701	23	-0.210925	34.99476	1170.7	0	STO	1.111	9.6
33	4331701		-0.210986667	34.99479167	1175.9	1	PHI	0.194	2.5
33	4331701		-0.21094	34.994815	1164.2	2	ENV	0.22	2.8
33	4331701		-0.210585	34.99452833	1160.5	3	EZO	0.45	7
33	4331701		-0.210906667	34.99484	1148.9	4	RKT	0.191	2.3
33	4331701		-0.210965	34.99484667	1150.5	5	ECH	0.705	8.2
33	4331801	23	-0.20948	34.983525	1150.2	0	STO	0.633	6.5
33	4331801		-0.209473333	34.98352833	1156.1	1	ENV	0.531	6.5
33	4331801		-0.20955	34.98355	1156.8	2	EZO	2.491	14.7
33	4331801		-0.209451667	34.98348	1153	3	PRA	2.516	22.1
33	4331801		-0.20942	34.98352	1156.6	4	ECH	5.147	48.7
33	4331801		-0.209441667	34.983195	1158.9	5	PHI	3.291	34.6
33	4331901	20	-0.211423333	34.986945	1160.8	0	STO	0.682	7.9
33	4331901		-0.211476667	34.98696333	1147.7	1	EZO	0.271	36.4
33	4331901		-0.21145	34.98696667	1147.3	2	PRA	0.195	2.3
33	4331901		-0.211425	9.86922E+12	1154.1	3	ENV	0.197	2
33	4331901		-0.211475	34.986915	1149.4	4	PHI	0.12	1.2
33	4331901		0	0	0	5	RKT	0.312	2.4
33	4331901		-0.211428333	34.98710167	1168.6	6	ECH	0.221	2
33	4332001	30	-0.211258333	34.98614167	1161.8	0	STO	0.766	7.3
33	4332001		-0.211278333	34.98614167	1139.9	1	PHI	0.713	7.2
33	4332001		-0.21126	34.986125	1161.8	2	ENV	0.435	6.7
33	4332001		-0.21133	34.98619333	1151.1	3	EZO	0.518	5.1
33	4332001		-0.211281667	34.98617333	1158.8	4	RKT	0.715	8.2
33	4332001		-0.211251667	34.98613333	1152.9	5	ECH	0.461	3.3
33	4332101	32	-0.212481667	34.98574	1159.8	0	STO	3.674	33.6
33	4332101		-0.212526667	34.98571833	1161.5	1	ENV	0.731	6.7
33	4332101		-0.212528333	34.98573667	1156.9	2	EZO	1.964	23.3
33	4332101		-0.212526667	34.98573	1158.3	3	PRA	3.25	37.1
33	4332101		-0.21246	34.98581167	1161	4	PHI	0.913	14
33	4332101		-0.212466667	34.98571333	1157.6	5	ECH	1.076	9.2
33	4332201	37	34.983835	2.27167E+12	1140	0	STO	0.755	7.6
33	4332201		-0.212223333	34.983815	1154.8	4	ECH	0.139	2.1
33	4332201		-0.21223	34.98382167	1156.4	5	ENV	0.838	4.4
33	4332201		-0.21227	34.983915	1194.6	6	PHI	0.386	4.6
37	14370101	33	-0.196645	34.98704667	1174.3	0	STO	0.828	8.7
37	14370101		-0.196855	34.98701333	1172.7	1	PRA	0.454	5.9
37	14370101		-0.196901667	34.986935	1156.5	2	EZO	0.501	7.6
37	14370101		-0.196953333	34.98705667	1150.2	3	ENV	0.215	4.1
37	14370101		-0.196883333	34.98702833	1158	4	ECH	0.874	10.3
37	14370101		-0.196886667	34.98698667	1155.8	5	PHI	0.073	3.7
37	14370101		-0.196898333	34.98693833	1169.9	6	RKT	0.204	3.7
37	14370201	32	-0.195395	34.99364	1178.5	0	STO	1.263	25.1

37	14370201					1	PHI	0.647	9.5
37	14370201		-0.195438333	34.993675	1167.7	2	EZO	0.635	11.9
37	14370301	35	-0.194501667	34.993885	1158.7	0	STO	0.321	3.3
37	14370301					1	ENV	0.478	3.5
37	14370301		-0.194423333	34.99385	1173.6	2	PRA	0.732	7.4
37	14370301		-0.194458333	34.99383167	1172	3	RKT	0.295	3.6
37	14370301		-0.194403333	34.993925	1169.7	4	ECH	0.305	4.1
37	14370301		-0.194263333	34.993905	1192.1	5	PHI	0.201	3.3
37	14370401	32	-0.196201667	34.98767333	1163.7	0	STO	0.347	5.9
37	14370401		-0.196191667	34.98766667	1163.1	1	PRA	0.041	0.2
37	14370401		-0.196183333	34.98766	1165.5	2	EZO	0.028	1.4
37	14370401		-0.196186667	34.987675	1174.9	3	ENV	0.161	2.6
37	14370401		-0.196175	34.98770167	1159	4	PHI	0.073	0.7
37	14370401		-0.196126667	34.987715	1172.2	5	RKT	0.17	3
37	14370501	34	0	0	0	0	STO	0.778	7
37	14370501		-0.195268333	34.99022833	1182.7	1	PRA	0.355	3.1
37	14370501		-0.195178333	34.99022667	1165.8	2	EZO	1.1	21.1
37	14370501		-0.195211667	34.99021167	1167.7	3	ENV	0.649	7.7
37	14370501		-0.195166667	34.99022167	1160.6	4	PHI	0.391	4.7
37	14370501		-0.195195	34.990255	1174.4	5	RKT	0.377	5.9
37	14370601	45	-0.18905	34.99194667	1182.3	0	STO	0.896	5.2
37	14370601					1	PRA	0.516	4.9
37	14370601		-0.189056667	34.99190833	1169.2	2	EZO	1.155	5
37	14370601		-0.18902	34.99192167	1175.7	3	ENV	0.33	4.1
37	14370601		-0.189128333	34.991935	1150.2	4	ECH	0.651	4.9
37	14370601		-0.189161667	34.99191167	1184.1	5	RKT	0.235	3.6
37	14370701	24	-0.181098333	34.99318333	1151.2	0	STO	1.096	15.5
37	14370701					1	EZO	0.844	14.1
37	14370701		-0.181165	34.993185	1171.2	2	ENV	0.492	9.2
37	14370701		-0.181091667	34.99320167	1151.9	3	RKT	0.686	11.6
37	14370701		-0.181148333	34.993175	1169.2	4	PRA	0.72	11.3
37	14370701		-0.181073333	34.99315333	1153	5	ECH	0.81	15.5
37	14370801	27	0	0	0	0	STO	0.131	1.1
37	14370801		0	0	0	1	PHI	0.226	3
37	14370801		-0.184841667	35.00087	1171.1	2	ENV	0.755	7.1
37	14370801		-0.184755	35.00107667	1166.8	3	EZO	1.04	8.6
37	14370801		-0.184458333	35.00080167	1150.4	4	RKT	2.892	21.4
37	14370801		-0.184826667	35.00105	1169.4	5	ECH	0.796	6.4
37	14370901	26	-0.18694	34.99778167	1172.8	0	STO	0.337	6.4
37	14370901		-0.187221667	34.99778167	1179.4	1	EZO	0.787	7.1
37	14370901		-0.186916667	34.99785	1184.8	2	ENV	0.666	5.5
37	14370901		-0.186188333	34.997765	1152	3	RKT	0.563	8.3
37	14370901		-0.186931667	34.99781	1167.1	4	PRA	0.119	1.8
37	14370901		-0.186948333	34.99785333	1172.2	5	ECH	1.547	15.7
37	14371001	25	0	0	0	0	STO	1.432	16.1
37	14371001		-0.18148	34.99195167	1175.1	1	ENV	0.788	8.8
37	14371001		-0.181628333	34.991645	1160.1	2	PRA	0.481	6.4

37	14371001		-0.181916667	34.99177	1159	3	RKT	0.362	6.8
37	14371001		-0.181476667	34.99196333	1164.4	4	ECH	0.74	11.8
37	14371001		-0.181426667	34.99195833	1167.2	5	PHI	1.009	11.6
37	14371101	39	-0.185551667	34.99511333	1174.1	0	STO	0.21	2.6
37	14371101		-0.185561667	34.99506833	1166.7	1	EZO	0.186	1.9
37	14371101		-0.185581667	34.99509333	1169.4	2	ENV	0.278	2.2
37	14371101		-0.185593333	34.99513	1164	3	RKT	0.903	11.1
37	14371101		-0.185515	34.99511167	1159.6	4	PRA	0.962	4.2
37	14371101		-0.185695	34.99509667	1170.6	5	ECH	0.242	2
37	14371201	23	-0.183163333	35.00013167	1157.3	0	STO	0.342	4.5
37	14371201		-0.183023333	35.00022667	1167.3	1	ENV	0.178	3.5
37	14371201		-0.183028333	35.00022	1167.6	2	PRA	0.1	0.8
37	14371201		-0.183033333	35.00021667	1166.6	3	PHI	0.18	1.7
37	14371201		-0.183025	35.00021333	1168.9	4	ECH	0.41	
37	14371301	30	0.197318333	35.24499667	1173.8	0	STO	0.333	6.7
37	14371301		-0.18251	34.99843833	1166.3	1	ENV	0.199	3.2
37	14371301		-0.182473333	34.998375	1171.8	2	PRA	0.25	4.2
37	14371301		-0.182518333	34.99839833	1166.6	4	ECH	1.255	12.7
37	14371301		-0.182506667	34.99841167	1171.1	5	EZO	0.251	4
37	14371401	27	-0.18338	34.993485	1170.1	0	STO	0.547	7.4
37	14371401					1	ENV	0.291	3.3
37	14371401		-0.183321667	34.99350333	1165.7	2	PRA	0.169	2.8
37	14371401		-0.183316667	34.99342333	1167.2	3	RKT	0.334	4
37	14371401		-0.183271667	34.993445	1160	4	ECH	0.155	1.9
37	14371401		-0.183403333	34.99344167	1161.7	5	PHI	0.405	2.4
37	14371501	40	-0.181633333	34.99047333	1158.7	0	STO	0.428	3.7
37	14371501		-0.181023333	34.99035667	1174.6	1	PRA	0.307	2
37	14371501		-0.181473333	34.99020167	1165.4	2	EZO	0.141	1.5
37	14371501		-0.18107	34.99036	1165.2	3	ENV	0.155	3
37	14371501		-0.181011667	34.990425	1143	4	ECH	0.549	3.9
37	14371501		-0.181555	34.99019833	1177.4	5	RKT	0.292	2.8
37	14371601	22	-0.188316667	34.98656	1157	0	STO	0.37	5
37	14371601		-0.188326667	34.98657333	1161.5	1	PRA	0.141	2.7
37	14371601		-0.18834	34.98650833	1155.3	2	ENV	0.357	6.7
37	14371601		-0.188305	34.98654333	1168.1	3	EZO	0.357	5.8
37	14371601		-0.188365	34.986505	1153.4	4	ECH	0.453	2.7
37	14371601		-0.18832	34.98651333	1171	5	PHI	0.206	2.5
37	14371701	38	-0.193395	34.985755	1167.1	0	STO	1.693	15.5
37	14371701		-0.193483333	34.98577333	1169.3	1	EZO	0.603	8.3
37	14371701		0	0	0	2	ENV	1.192	4.9
37	14371701		-0.193538333	34.98571333	1168.9	3	PRA	3.682	43.5
37	14371701		-0.193483333	34.98582	1158.2	4	RKT	0.38	3.8
37	14371701		-0.193436667	34.98582167	1164.9	5	ECH	0.376	3.3
37	14371801	22	-0.18488	34.99840667	1164.4	0	STO	0.168	2.2
37	14371801		-0.184951667	34.99839	1174	1	EZO	0.176	2.2
37	14371801		-0.184903333	34.99839167	1170.7	2	ENV	0.134	1.5
37	14371801		-0.184825	34.99852333	1149.4	3	PRA	0.753	5.4

37	14371801		-0.184895	34.998415	1163.7	4	PHI	0.275	4.1
37	14371801		-0.184993333	34.99833833	1175	5	RKT	0.172	1.9
37	14371901	23	-0.181753333	34.99173667	1180.8	0	STO	1.318	14.4
37	14371901		-0.181675	34.99169833	1165.9	1	EZO	1.34	11.9
37	14371901		-0.181675	34.99168333	1165.5	2	PHI	0.555	6.4
37	14371901		-0.181675	34.99171	1164.7	3	PRA	0.139	3.2
37	14371901		-0.181658333	34.99172	1158.9	4	RKT	2.261	18.8
37	14372001	25	-0.19952	34.99556167	1186.3	0	STO	0.337	2.8
37	14372001		-0.199386667	34.99541167	1172.8	1	PHI	0.219	1.4
37	14372001		-0.199433333	34.99537167	1176.7	2	ENV	0.157	2.4
37	14372001		-0.199476667	34.99551667	1171.9	3	EZO	0.53	9.5
37	14372001		-0.19909	34.99488167	1167.4	4	RKT	0.175	3
37	14372001		-0.199036667	34.99549333	1173.9	5	ECH	0.256	3.2
37	14372101	20	-0.186353333	34.99634667	1168.9	0	STO	0.909	8.7
37	14372101		-0.186261667	34.99628	1170.4	1	ENV	0.218	2.8
37	14372101		-0.18619	34.99645	1161.2	2	PRA	0.197	2
37	14372101		-0.186276667	34.99627833	1171.6	3	RKT	0.482	4.9
37	14372101		-0.186333333	34.99632167	1174.1	4	ECH	0.239	2.1
37	14372101		-0.186265	34.99634667	1171.9	5	PHI	0.211	1.2
37	14372201	23	0	0	0	0	STO	1.139	17
37	14372201		-0.181701667	34.98873333	1135.4	1	EZO	1.016	11.3
37	14372201		0	0	0	2	ENV	0.916	10.1
37	14372201		-0.181815	34.98880333	1161.9	3	PRA	0.96	13
37	14372201		-0.191271667	34.98879333	2741	4	RKT	1.23	15.8
37	14372201		-0.181893333	34.98888	1166.5	5	ECH	0.658	6.6
37	14372301	42	-0.194381667	34.99471667	1172.5	0	STO	0.309	
37	14372301		-0.194328333	34.99479833	1193.8	4	PRA	0.372	4.6
37	14372301		-0.194308333	34.994765	1170.9	5	ECH	0.455	4.8

mean_com	mass_fuel	taltotaldur	talavgdur	taltotallampdur	totcookevent	lampuse	talstovestack
2		16:43:00	1:31:11	10:14:00	11	1	0
1.3	28.97	17:30:00	1:20:46	8:00:00	13	1	0
0.6	4.28	13:30:00	2:15:00	8:29:00	6	1	1
0.7	12.28	12:22:00	1:32:45	6:44:00	8	1	0
1.3		9:05:00	1:30:50	5:17:00	6	1	0
2.3	3.66	21:52:00	3:07:26	6:23:00	7	1	1
	6.86	12:44:00	2:07:20	3:55:00	6	1	0
3.3	26.56	14:07:00	1:45:53	10:34:00	8	1	0
1.8	16.88	7:50:00	0:58:45	8:00:00	8	1	0
2.1	12.78	7:50:00	1:18:20	6:00:00	6	1	0
2.1	10.40	8:03:00	1:20:30	8:18:00	6	1	1
1.3	11.66	5:33:00	0:55:30	9:07:00	6	1	0
0.8	12.00	5:08:00	1:01:36	7:58:00	5	1	0
1.2	7.94	10:17:00	1:42:50	9:00:00	6	1	0
0.7	6.74	1:51:00	0:27:45	2:30:00	4	1	0
0.1	5.85	4:55:00	0:36:52	4:30:00	8	1	1
1.6	3.48	4:24:00	0:37:43	4:20:00	7	1	0
0.4	6.74	4:45:00	0:35:37	5:20:00	8	1	1
0.3	4.36	4:00:00	0:40:00	4:40:00	6	1	1
0.4	13.76	4:00:00	0:40:00	4:05:00	6	1	1
	6.06	5:03:00	1:15:45	2:38:00	4	1	0
0.2	11.94	5:40:00	0:42:30	5:00:00	8	1	1
1.4	1.89	6:49:00	1:08:10	3:21:00	6	1	0
1.2	5.84	4:38:00	0:39:43	2:41:00	7	1	1
0.9	8.44	7:11:00	1:11:50	6:07:00	6	1	1
0.6	20.52	3:56:00	0:39:20	3:30:00	6	1	1
3.2	6.52	6:27:00	1:04:30	4:53:00	6	1	0
1.8	5.84	5:42:00	1:08:24	5:25:00	5	1	0
0.6	5.84	6:41:00	1:40:15	4:46:00	4	1	0
1.4	5.28	5:57:00	0:59:30	6:22:00	6	1	0
0.7	4.30	5:48:00	0:58:00	4:26:00	6	1	0
	4.74	5:21:00	0:53:30	6:06:00	6	1	0
1.2	3.44	5:47:00	0:57:50	6:15:00	6	1	0
8.9	15.67	23:40:00	2:09:05	7:00:00	11	1	0
9.3	10.75	21:20:00	3:01:20	8:00:00	15	1	1
26.2	7.00	16:25:00	1:49:27	6:15:00	9	1	1
21.7	7.50	11:10:00	1:51:40	6:00:00	6	1	1
2.1	19.92	10:36:00	1:46:00	5:00:00	6	1	0
5.1	13.64	12:45:00	2:07:30	5:29:00	6	1	0
6.5	10.46	7:40:00	1:32:00	5:00:00	5	1	0
6.8	10.30	10:20:00	0:56:22	5:20:00	11	1	0
5	9.70	7:58:00	1:19:40	5:00:00	6	1	0
1	4.70	4:20:00	0:43:20	6:00:00	6	1	0
2.2	6.42	7:00:00	1:00:00	4:30:00	7	1	0
2.9	9.44	4:30:00	1:07:30	4:30:00	4	1	0
6.1	7.94	11:20:00	1:53:20	6:00:00	6	1	0

	12.20	7:05:00	0:42:30	5:00:00	10	1	1
0.2	7.04	8:30:00	0:51:00	7:00:00	10	1	0
	27.22	11:45:00	0:58:45	6:00:00	12	1	1
0	7.08	13:30:00	1:02:18	9:20:00	13	1	0
0.3	7.30	8:30:00	0:42:30	4:50:00	12	1	0
0.1	7.30	9:25:00	0:40:21	6:45:00	14	1	0
2.6	9.36	4:55:00	0:59:00	5:00:00	5	1	1
0.8	16.22	4:46:00	0:47:40	5:00:00	6	1	0
2.9	6.34	7:50:00	1:18:20	5:15:00	6	1	1
4.4	3.20	4:40:00	0:46:40	5:15:00	6	1	1
	6.10	2:14:00	0:33:30	3:10:00	4	1	0
9.8	9.14	6:40:00	1:06:40	6:45:00	6	1	1
0.2	9.54	5:45:00	0:57:30	5:15:00	6	1	1
0.9	14.50	3:21:00	0:50:15	3:29:00	4	1	0
0.9	6.44	6:07:00	1:01:10	3:00:00	6	1	0
	3.70	6:25:00	1:04:10	2:28:00	6	1	0
0.9	3.32	5:18:00	1:03:36	2:51:00	5	1	0
0.5	3.40	5:32:00	0:55:20	2:10:00	6	1	1
0.4	4.88	4:28:00	0:53:36	2:00:00	5	1	0
3.3	15.46	3:45:00	1:52:30	3:15:00	2	1	0
0.9	7.34	13:25:00	1:40:37	6:00:00	8	1	1
	25.18	15:15:00	1:54:22	10:45:00	8	1	1
1.2	9.72	16:08:00	2:01:00	9:32:00	8	1	1
1.5	12.36	5:38:00	0:48:17	6:00:00	7	1	1
1.4	12.14	14:07:00	1:45:53	12:13:00	8	1	1
1	7.82	7:45:00	0:46:30	4:30:00	10	1	0
1.8	13.24	6:45:00	1:07:30	4:00:00	6	1	0
0.1	4.54	8:30:00	2:07:30	5:00:00	4	1	0
5.8	8.66	12:40:00	1:48:34	3:00:00	7	1	0
0.9	2.70	5:30:00	1:06:00	3:00:00	5	1	0
0.9	9.34	2:25:00	0:48:20	2:00:00	3	1	0
0.3	8.26	19:00:00	1:27:42	6:00:00	13	1	1
0.5	10.28	4:35:00	0:45:50	4:00:00	6	1	1
4	7.42	8:00:00	1:20:00	7:00:00	6	1	0
4.3	10.10	0:00:00	2:40:00	15:30:00	9	1	0
1.6	5.04	6:00:00	1:00:00	6:00:00	6	1	0
0.3	11.50	10:05:00	1:26:26	6:00:00	7	1	0
1	4.54	8:15:00	1:22:30	4:00:00	6	1	0
1	10.68	8:30:00	1:12:51	6:00:00	7	1	0
3.8	19.00	16:51:00	2:24:26	7:00:00	7	1	0
4.1	16.94	16:30:00	2:45:00	8:00:00	6	1	1
2.3	14.38	14:37:00	2:05:17	8:00:00	7	1	0
	10.78	12:54:00	1:36:45	8:20:00	8	1	0
2.2	8.30	12:20:00	1:32:30	8:30:00	8	1	0
3.4	6.64	9:20:00	1:10:00	7:35:00	8	1	0
1.9	16.72	7:51:00	1:07:17	5:53:00	7	1	0
2.1	10.02	9:35:00	1:11:53	3:30:00	8	1	0

1.5	5.44	5:08:00	0:44:00	3:02:00	7	1	0
0.8	7.14	6:28:00	0:55:26	3:49:00	7	1	0
1.5	10.12	5:44:00	0:57:20	3:37:00	6	1	0
1.4	5.98	8:27:00	1:24:30	2:28:00	6	1	0
7.5	13.19	8:02:00	1:08:51	6:54:00	7	1	0
1	7.40	7:04:00	1:00:34	6:19:00	7	1	0
1.4	13.96	4:28:00	0:38:17	5:14:00	7	1	0
2	5.54	5:32:00	1:06:24	5:25:00	5	1	0
2.8	7.62	7:00:00	1:00:00	4:25:00	7	1	0
1.6	6.48	8:44:00	1:27:20	6:33:00	6	1	0
5.6	16.76	11:52:00	1:11:12	8:44:00	10	1	0
3.5	14.98	12:30:00	1:08:11	8:50:00	11	1	0
5.1	7.50	18:40:00	1:33:20	7:30:00	12	1	1
3.5	5.92	5:30:00	1:06:00	5:00:00	5	1	1
7.2	9.56	1:10:00	1:10:00		1	0	0
		8:00:00	2:00:00	6:00:00	4	1	0
2.1	10.46	5:49:00	1:27:15	3:57:00	4	1	0
1.3	9.48	6:03:00	1:12:36	7:28:00	5	1	0
2.6	11.52	4:43:00	0:56:36	5:45:00	5	1	0
0.9	5.64	8:31:00	1:25:10	6:48:00	6	1	0
0.7	1.76	3:15:00	0:39:00	7:32:00	5	1	0
1.2	8.24	16:53:00	2:48:50	8:28:00	6	1	0
1.2	5.58	6:33:00	1:18:36	6:33:00	5	1	0
4	11.69	12:03:00	1:12:18	10:00:00	10	1	0
1.6	7.50	6:56:00	0:34:40	8:22:00	12	1	0
2.6	9.94	11:53:00	0:54:51	9:58:00	13	1	0
2.5	6.10	8:10:00	1:01:15	6:00:00	8	1	0
3.1	12.44	8:47:00	0:47:55	4:00:00	11	1	0
1	7.90	7:05:00	1:10:50	4:12:00	6	1	0
	16.78	9:42:00	1:37:00	10:00:00	6	1	0
0.7	16.00	3:39:00	0:36:30	3:54:00	6	1	0
1.9	11.44	4:36:00	0:27:36	6:44:00	10	1	0
2.8	15.28	8:38:00	1:26:20	8:35:00	6	1	1
1.2	6.52	2:25:00	0:20:43	8:19:00	7	1	1
1.9	13.24	5:13:00	0:52:10	10:30:00	6	1	0
2.9	17.92	7:15:00	1:02:09	7:15:00	7	1	0
1.2	4.64	9:30:00	1:21:26	7:15:00	7	1	0
0.4	8.00	8:15:00	1:22:30	6:00:00	6	1	0
1.3	9.68	4:25:00	0:44:10	6:00:00	6	1	1
0.5	10.21	17:00:00	2:07:30	7:00:00	8	1	0
0.1	6.88	9:50:00	1:13:45	8:00:00	8	1	0
0.5	9.42	7:20:00	1:13:20	6:00:00	6	1	0
0.5	7.14	7:00:00	1:00:00	4:00:00	7	1	1
0.1	9.00	5:10:00	0:44:17	8:00:00	7	1	0
0.1	8.19	7:40:00	1:16:40	5:00:00	6	1	0
0	7.66	9:10:00	1:18:34	4:00:00	7	1	1
1.2	10.12	6:59:00	1:23:48	6:45:00	5	1	1

0.5	4.80	5:30:00	0:55:00	6:08:00	6	1	1
1.3	11.38	3:37:00	0:36:10	6:28:00	6	1	0
2.1	7.96	6:00:00	0:45:00	5:25:00	8	1	0
2.5	7.02	4:00:00	0:34:17	6:30:00	7	1	0
4.2	6.82	6:45:00	1:21:00	6:50:00	5	1	0
2.2	7.50	6:40:00	1:06:40	5:50:00	6	1	1
1	9.35	5:00:00	1:00:00	5:00:00	5	1	0
1.5	4.46	3:10:00	0:31:40	6:00:00	6	1	1
1.8	11.60	9:00:00	1:30:00	5:00:00	6	1	0
0	3.24	8:05:00	1:20:50	3:20:00	6	1	0
0.6	5.94	8:00:00	1:20:00	4:30:00	6	1	0
0.4	4.16	4:45:00	0:47:30	5:30:00	6	1	0
0.5	2.18	2:30:00	0:37:30	5:00:00	4	1	0
3	8.20	15:00:00	2:08:34	4:30:00	7	1	0
1	11.92	5:44:00	1:08:48	5:05:00	5	1	1
0.7	14.48	11:15:00	2:15:00	6:15:00	5	1	0
	19.54	7:48:00	1:06:51	4:52:00	7	1	0
1.1	10.92	8:31:00	1:13:00	5:05:00	7	1	0
1.4	4.64	5:37:00	0:56:10	4:40:00	6	1	0
1.5	7.58	4:52:00	0:48:40	4:16:00	6	1	0
	28.82	7:15:00	1:12:30	10:00:00	6	1	0
0.5	16.46	10:00:00	2:00:00	8:30:00	5	1	1
0	9.53	5:36:00	0:56:00	9:00:00	6	1	1
3.5	17.88	10:24:00	1:29:09	12:00:00	7	1	1
1.3	12.78	10:39:00	1:31:17	12:00:00	7	1	1
0.1	11.42	3:30:00	0:42:00	7:00:00	5	1	1
5.5	9.72	9:45:00	0:58:30	7:30:00	10	1	0
0.2	9.50	10:43:00	0:58:27	12:00:00	11	1	1
0.2	2.10	7:25:00	1:14:10	7:00:00	6	1	1
0.9	5.94	7:50:00	1:34:00	8:00:00	5	1	1
0	4.56	7:00:00	1:24:00	8:00:00	5	1	1
0.2	2.60	10:00:00	1:40:00	8:00:00	6	1	1
0	5.84	5:35:00	0:47:51	6:10:00	7	1	0
1	1.76	3:35:00	0:30:43	7:53:00	7	1	0
	6.00	4:59:00	0:42:43	5:30:00	7	1	0
2	5.92	5:10:00	0:44:17	5:53:00	7	1	0
2.2	6.36	6:10:00	1:01:40	4:00:00	6	1	0
0.3	2.38	6:05:00	1:00:50	6:30:00	6	1	0
1.5	7.80	10:22:00	1:09:07	7:10:00	9	1	0
1.1	5.34	8:50:00	1:06:15	7:50:00	8	1	0
0.3	5.26	8:38:00	1:26:20	4:50:00	6	1	1
0.7	4.94	8:35:00	1:13:34	5:00:00	7	1	0
0.1	4.00	5:50:00	1:10:00	5:05:00	5	1	0
0	4.50	6:35:00	1:19:00	5:55:00	5	1	1
1.5	15.52	19:00:00	3:10:00	6:00:00	6	1	0
0.4	16.50	8:20:00	1:40:00	4:30:00	5	1	0
2.5	2.40	7:29:00	1:14:50	5:00:00	6	1	1

0.3	12.34	7:00:00	1:24:00	4:00:00	5	1	1
0	7.32	5:39:00	1:24:45	4:00:00	4	1	1
0	0.58	7:00:00	1:24:00	4:30:00	5	1	1
1.6	6.30	9:30:00	1:21:26	6:00:00	7	1	0
1.2	10.40	12:30:00	1:33:45	4:30:00	8	1	0
1.6	10.22	8:00:00	1:08:34	6:30:00	7	1	1
1.8	8.02	8:30:00	1:25:00	5:30:00	6	1	0
0.7	5.18	9:30:00	1:21:26	3:30:00	7	1	1
1	3.22	5:30:00	0:55:00	6:00:00	6	1	1
2.2	6.22	3:00:00	1:55:43	4:00:00	14	1	0
0.8	5.24	13:00:00	1:05:00	4:00:00	12	1	0
0.4	5.00	7:30:00	1:15:00	4:30:00	6	1	0
0.7	3.30	4:52:00	0:36:30	4:30:00	8	1	0
1.8	4.24	5:00:00	1:15:00	4:30:00	4	1	0
2.2	8.94	20:30:00	2:33:45	7:30:00	8	1	0
2.3	8.06	16:00:00	1:36:00	5:47:00	10	1	0
2	6.14	7:30:00	1:52:30	5:00:00	4	1	0
2.6	4.50	6:00:00	1:30:00	6:00:00	4	1	0
1.6	2.46	8:30:00	1:42:00	5:00:00	5	1	0
3.5	10.32	5:30:00	0:55:00	4:00:00	6	1	0
1.5	8.00	4:15:00	0:36:26	3:30:00	7	1	0
0.8	5.88	6:00:00	1:30:00	4:30:00	4	1	0
2.8	5.36	7:30:00	1:52:30	7:30:00	4	1	0
0.9	4.14	3:30:00	0:42:00	4:30:00	5	1	0
1	6.48	5:00:00	0:50:00	8:45:00	6	1	1
2.6	13.72	3:28:00	1:09:20	6:56:00	3	1	0
0.1	12.56	9:52:00	1:24:34	5:30:00	7	1	0
0.6	8.63	5:24:00	0:54:00	9:00:00	6	1	0
2.2	8.40	5:52:00	0:58:40	9:14:00	6	1	0
1.4	10.86	7:11:00	1:01:34	9:07:00	7	1	1
0.3	4.44	6:50:00	1:22:00	6:00:00	5	1	0
2.4	8.77	5:00:00	0:37:30	3:30:00	8	1	0
2.1	2.96	7:00:00	1:00:00	5:00:00	7	1	1
1.8	3.89	2:45:00	0:27:30	3:30:00	6	1	1
2.1	4.96	4:00:00	0:34:17	5:00:00	7	1	0
0.9	7.92	4:30:00	0:38:34	6:00:00	7	1	1
1.5	2.72	3:55:00	0:33:34	4:00:00	7	1	1
9.4	23.88	12:00:00	2:00:00	7:00:00	6	1	0
3.2	11.70	13:00:00	1:51:26	7:00:00	7	1	0
4.9	14.00	11:30:00	1:16:40	13:10:00	9	1	1
6.7	15.30	11:00:00	1:22:30	14:30:00	8	1	1
2.3	11.46	11:10:00	1:23:45	7:00:00	8	1	1
2.1	8.17	14:45:00	1:38:20	10:00:00	9	1	1
0.7	5.30	12:41:00	1:03:25	6:00:00	12	1	0
1.4	9.70	12:50:00	1:10:00	5:30:00	11	1	0
1.1	5.32	7:09:00	1:25:48	7:00:00	5	1	0
1.1	4.54	8:40:00	1:26:40	5:00:00	6	1	0

1.4	4.56	6:36:00	1:19:12	4:45:00	5	1	0
4	5.92	6:00:00	1:12:00	5:35:00	5	1	0
1.1	7.56	5:50:00	1:10:00	4:40:00	5	1	0
0.8	1.98	7:12:00	1:26:24	6:15:00	5	1	1
0.2	3.72	3:15:00	0:32:30	5:59:00	6	1	0
0.5	6.18	7:47:00	1:17:50	6:21:00	6	1	0
0.2	5.22	4:43:00	1:10:45	4:55:00	4	1	1
3.8	3.10	13:00:00	1:51:26	8:00:00	7	1	0
0.5	2.40	8:30:00	1:12:51	7:00:00	7	1	0
1.3	5.88	5:23:00	0:53:50	7:00:00	6	1	0
3.1	3.14	9:30:00	1:54:00	9:00:00	5	1	0
0.6	5.20	4:34:00	0:45:40	7:00:00	6	1	0
2.5	5.94	3:18:00	0:39:36	9:00:00	5	1	0
3	10.80	8:45:00	1:27:30	6:02:00	6	1	0
0.6	6.76	4:43:00	0:40:26	5:04:00	7	1	0
1.7	5.48	4:53:00	0:41:51	6:47:00	7	1	0
1.9	6.10	4:40:00	0:46:40	5:44:00	6	1	0
1.6	5.52	2:54:00	0:34:48	4:20:00	5	1	0
2	4.76	3:04:00	0:36:48	4:17:00	5	1	1
1.3	12.14	14:00:00	1:33:20	6:00:00	9	1	0
0.6	2.70	11:09:00	1:06:54	6:30:00	10	1	1
1.3	8.88	10:20:00	0:56:22	6:00:00	11	1	1
0.8	2.14	8:00:00	1:00:00	6:30:00	8	1	1
0.3	9.50	9:00:00	1:07:30	6:00:00	8	1	1
0.2	6.96	7:10:00	0:53:45	6:00:00	8	1	0
	14.30	8:10:00	1:21:40	5:30:00	6	1	0
	41.10	8:10:00	0:54:27	6:45:00	9	1	0
0.6	7.18	7:25:00	0:55:38	8:00:00	8	1	1
