# Supplementary material for: Effectiveness of Six Improved Cookstoves in Reducing Household Air Pollution and Their Acceptability in Rural Western Kenya
Source: PLoS One. 2016 Nov 15;11(11):e0165529. doi: 10.1371/journal.pone.0165529 (PMC5112915; doi:10.1371/journal.pone.0165529)
Supplement: S5 File — (PDF) [file pone.0165529.s005.pdf]

CAL RESEAR[illegible]TUTE
[illegible] GLOBAL HEALTH RESEARCH
12 JAN 2015
[illegible] KISUMU

KENYA MEDICAL RESEARCH INSTITUTE

P.O. Box 54840-00200, NAIROBI, Kenya
Tel (254) (020) 2722541, 2713349, 0722-205901, 0733-400003; Fax: (254) (020) 2720030
E-mail: director@kemri.org info@kemri.org Website:www.kemri.org

KEMRI/RES/7/3/1 **December 16, 2014**

TO: **TAMARA PILISHVILI**
PRINCIPAL INVESTIGATOR

THROUGH: **DR. STEPHEN MUNGA,**
THE DIRECTOR, CGHR,
KISUMU

FORWARDED
14/1/2015
DIRECTOR
CENTRE FOR GLOBAL HEALTH RESEARCH

Dear Madam,

RE: SSC PROTOCOL No. 2075 (*REQUEST FOR ANNUAL RENEWAL*): EVALUATION OF ACCEPTABILTY AND SUSTAINABILITY OF IMPROVED STOVES AND THEIR IMPACT ON INDOOR AIR QUALITY AND CHILD HELATH IN RURAL WESTERN KENYA

Thank you for the continuing review report for the period **January 2012** to **November 2014.**

This is to inform that during the 234th meeting of the KEMRI/Scientific and Ethics Review Committee (SERU) held on **16th December, 2014**, the Committee **conducted the annual review and approved** the above referenced application for another year.

This approval is valid from **17th February, 2015** through to **16th February, 2016**. Please note that authorization to conduct this study will automatically expire on **February 16, 2016**. If you plan to continue with data collection or analysis beyond this date please submit an application for continuing approval to the **SERU** secretariat by **5th January, 2016.**

You are required to submit any amendments to this protocol and any other information pertinent to human participation in this study to SERU for review prior to initiation.

Yours faithfully,

EAB

PROF. ELIZABETH BUKUSI,
ACTING SECRETARY,
KEMRI ETHICS REVIEW COMMITTEE
