# Supplementary material for: Effectiveness of Six Improved Cookstoves in Reducing Household Air Pollution and Their Acceptability in Rural Western Kenya
Source: PLoS One. 2016 Nov 15;11(11):e0165529. doi: 10.1371/journal.pone.0165529 (PMC5112915; doi:10.1371/journal.pone.0165529)
Supplement: S6 File — (DOCX) [file pone.0165529.s006.docx]

#
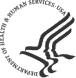
Memorandum

September 11, 2015September 4th, 2012

Date

d

From

Felecia Peterson

IRB-G Administrator, Human Research Protection Office

Subject

Site-Restricted CDC IRB Approval of Continuation #4 of Protocol #61556155.0, “EVALUATION OF ACCEPTABILITY AND SUSTAINABILITY OF IMPROVED STOVES AND THEIR IMPACT ON INDOOR AIR QUALITY AND CHILD HEALTHIN RURAL WESTERN KENYAEVALUATION OF ACCEPTABILITY AND SUSTAINABILITY OF IMPROVED STOVES AND THEIR IMPACT ON INDOOR AIR QUALITY AND CHILD HEALTHIN RURAL WESTERN KENYA” (Expedited)

To

Tamara Pilishvili, MPH

NCIRD/DBD

CDC's IRB "G” has reviewed and approved the request to continue protocol #6155.0, “EVALUATION OF ACCEPTABILITY AND SUSTAINABILITY OF IMPROVED STOVES AND THEIR IMPACT ON INDOOR AIR QUALITY AND CHILD HEALTHIN RURAL WESTERN KENYA”, for the maximum allowable period of one year. CDC IRB approval will expire on **9/12/20139/12/2016**. The continuation action was reviewed in accordance with the expedited review process outlined in 45 CFR 46.110(b)(1), categories 2, 3, 4, 6, & 7.

**COLLABORATOR/SITE RESTRICTION: Institutions that receive federal support who are engaged in human subjects’ research are required to obtain and provide documentation of IRB approval and maintain a valid Federal wide Assurance (FWA). CDC investigators who interact with institutions that have failed to meet these requirements are collaborating with noncompliant institutions. Study activities may not begin with the collaborators listed below until documentation indicating current IRB approval has been received by CDC’s Human Research Protection Office (HRPO) and the PI has been notified by HRPO that this restriction has been lifted and study activities may begin:**

1. **Need current Local IRB Approval documentation for World Health Organization**

If other institutions involved in this protocol are being awarded CDC funds through the CDC Procurement and Grants Office (PGO), you are required to send a copy of this IRB approval to the CDC PGO award specialist handling the award. You are also required to verify with the award specialist that the awardee has provided PGO with the required documentation and has approval to begin or continue research involving human subjects as described in this protocol.

As a reminder, the IRB must review and approve all human subjects research protocols at intervals appropriate to the degree of risk, but not less than once per year. There is no grace period beyond one year from the last IRB approval date. It is ultimately your responsibility to submit your research protocol for continuation review and approval by the IRB along with available IRB approvals from all collaborators. Please keep this approval in your protocol file as

proof of IRB approval and as a reminder of the expiration date. **To avoid lapses in approval of your research and the possible suspension of subject enrollment and/or termination of the protocol, please submit your continuation request along with all completed supporting documentation at least six weeks before the protocol's expiration date of 9/12/2016.**

**Any problems of a serious nature must be brought to the immediate attention of the CDC IRB, and any proposed changes to the protocol are required to be submitted as an amendment to the protocol for CDC IRB approval before they are implemented.**

If you have any questions, please contact your National Center Human Subjects Contact or the CDC Human Research Protection Office (404) 639-7570 or e-mail: [huma@cdc.gov](mailto:huma@cdc.gov).

cc:

NCIRD Human Studies Review
